# Co-circulation of genetically distinct highly pathogenic avian influenza A clade 2.3.4.4 (H5N6) viruses in wild waterfowl and poultry in Europe and East Asia, 2017–18

**DOI:** 10.1093/ve/vez004

**Published:** 2019-04-22

**Authors:** Marjolein J Poen, Divya Venkatesh, Theo M Bestebroer, Oanh Vuong, Rachel D Scheuer, Bas B Oude Munnink, Dennis de Meulder, Mathilde Richard, Thijs Kuiken, Marion P G Koopmans, Leon Kelder, Yong-Joo Kim, Youn-Jeong Lee, Mieke Steensels, Benedicte Lambrecht, Adam Dan, Anne Pohlmann, Martin Beer, Vladimir Savic, Ian H Brown, Ron A M Fouchier, Nicola S Lewis

**Affiliations:** 1 Department of Viroscience, Erasmus MC, Rotterdam, the Netherlands; 2 Department of Zoology, University of Cambridge, Cambridge CB2 3EJ, UK; 3 Staatsbosbeheer, Amersfoort, the Netherlands; 4 Avian Influenza Research and Diagnostic Division, Animal and Plant Quarantine Agency, Republic of Korea; 5 Avian Virology and Immunology, Sciensano, Brussels, Belgium; 6 Veterinary Diagnostics Directorate, Budapest, Hungary; 7 Institute of Diagnostic Virology, Friedrich-Loeffler-Institut, Insel Riems, Germany; 8 Croatian Veterinary Institute, Zagreb, Croatia; 9 OIE/FAO/EURL International Reference Laboratory for Avian Influenza, Swine Influenza and Newcastle Disease, Animal and Plant Health Agency (APHA)—Weybridge, Addlestone, Surrey, UK; 10 Department of Pathobiology and Population Sciences, Royal Veterinary College, Hawkshead Lane, North Mymms, Hatfield, Hertfordshire, AL9 7TA, UK

**Keywords:** virology, H5N6, phylogeny, avian influenza, emerging diseases, highly pathogenic avian influenza

## Abstract

Highly pathogenic avian influenza (HPAI) H5 clade 2.3.4.4 viruses were first introduced into Europe in late 2014 and re-introduced in late 2016, following detections in Asia and Russia. In contrast to the 2014–15 H5N8 wave, there was substantial local virus amplification in wild birds in Europe in 2016–17 and associated wild bird mortality, with evidence for occasional gene exchange with low pathogenic avian influenza (LPAI) viruses. Since December 2017, several European countries have again reported events or outbreaks with HPAI H5N6 reassortant viruses in both wild birds and poultry, respectively. Previous phylogenetic studies have shown that the two earliest incursions of HPAI H5N8 viruses originated in Southeast Asia and subsequently spread to Europe. In contrast, this study indicates that recent HPAI H5N6 viruses evolved from the H5N8 2016–17 viruses during 2017 by reassortment of a European HPAI H5N8 virus and wild host reservoir LPAI viruses. The genetic and phenotypic differences between these outbreaks and the continuing detections of HPAI viruses in Europe are a cause of concern for both animal and human health. The current co-circulation of potentially zoonotic HPAI and LPAI virus strains in Asia warrants the determination of drivers responsible for the global spread of Asian lineage viruses and the potential threat they pose to public health.

## 1. Introduction

**Figure 1.**
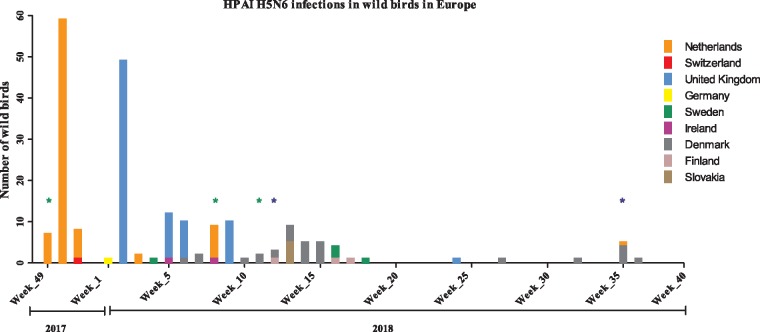
Overview of the number of wild birds reported to be infected with HPAI H5N6 based on the OIE update on avian influenza in animals (types H5 and H7) list 2017/2018 (OIE 2017; OIE 2018) per week starting from the first detection on 7 December 2017. The colours represent the country of detection. The asterisks (*) indicate the detections of HPAI H5N6 viruses in commercial (green) and backyard (blue) poultry.

Highly pathogenic avian influenza (HPAI) viruses cause outbreaks of disease, often resulting in mortality in poultry and wild bird species. Since 2003, HPAI H5 viruses of the A/Goose/Guangdong/1/1996 (GsGd) lineage have been circulating enzootically in poultry in several countries in South and Southeast Asia, and Africa. Periodically, these HPAI H5 viruses have been introduced into wild birds with subsequent spread to other geographic areas, likely through bird migration (Verhagen, Herfst and Fouchier 2015; Lycett et al. 2016). Since late 2013, HPAI viruses with an H5 heamagglutinin (HA) from clade 2.3.4.4 viruses with different neuraminidase (NA) subtypes (e.g. H5N8, H5N6, H5N1) have been circulating in Southeast Asia. Clade 2.3.4.4 (H5N8) viruses of two distinct groups, commonly referred to as Group A (Buan-like) and Group B (Gochang-like), were first detected in China and South Korea in late 2013/early 2014 (Ku et al. 2014; Wu et al. 2014; Zhou et al. 2016). Viruses belonging to Group A emerged in late 2014 and spread to North America and Europe almost simultaneously. After their initial detection, clade 2.3.4.4 Group B (H5N8) viruses were not detected in Southeast Asia until May 2016, when they were detected at Lake Uvs-Nur, Russia (Lee et al. 2017a) and Qinghai lake, China (Li et al. 2017). From May 2016, the clade 2.3.4.4 Group B H5N8 viruses subsequently spread to most European countries causing numerous outbreaks in poultry (OIE 2016) and massive die-offs in wild birds (Beerens et al. 2017; Kleyheeg et al. 2017; Pohlmann et al. 2017), and infected both poultry and wild birds in multiple African countries (OIE 2016, 2017; Twabela et al. 2018). Several reassortment events also occurred, leading to the emergence and detection of HPAI H5N5 in several European countries, Georgia and Israel between November 2016 and June 2017 (Alarcon et al. 2018) and HPAI H5N6 in Greece in February 2017 (OIE 2017). HPAI H5N6 first emerged in poultry in early 2014 having previously been detected in an environmental sample in late 2013 and in a live duck, sampled in early 2014 in China. Genetic analysis showed this HPAI H5N6 virus to be a reassortant consisting of a clade 2.3.4.4 HA, an NA related to Chinese low pathogenic avian influenza (LPAI) H6N6 duck viruses, and an internal gene cassette closely related to 2011 HPAI H5 clade 2.3.2.1 viruses (FAO 2014; Bi et al. 2016a,b). The first viruses were later assigned to Groups C and D. Currently, clade 2.3.4.4 Group C (H5N6 viruses from China in 2013 (Qi et al. 2014), Laos and Vietnam in 2014 and Hong Kong in 2015, and H5N1 viruses from China and Vietnam in 2014) and Group D (H5N6 viruses from China and Vietnam 2013–14, including human strains) viruses have also been classified as sub-groups (Lee et al. 2016, 2017b) although this nomenclature has not been formally adopted. HPAI H5N6 viruses belonging to clade 2.3.4.4 Group B were first detected in late 2017 in Japan, South Korea (Kim et al. 2018; Lee et al. 2018a), and the Netherlands (Beerens et al. 2018). From mid-December 2017, HPAI H5N6 Group B viruses were also detected in wild birds in Switzerland, UK, Germany, Sweden, Ireland, Denmark, Iran, Slovakia, and Finland (Fig. 1). In the Netherlands and Germany, the virus was also detected in poultry. In the same period, HPAI H5N6 viruses of clade 2.3.4.4 Group B, along with clade 2.3.4.4 Groups C and D have been detected in East Asian countries (Lee et al. 2018a).

HPAI H5 viruses pose a significant threat to not only animal health, particularly to poultry, but also to human health owing to their zoonotic potential (Adlhoch et al. 2016, 2018). In April 2014, the first fatal human case of HPAI H5N6 virus infection was identified in China (Pan et al. 2016), with these viruses phylogenetically clustering in unofficially-defined clade 2.3.4.4 Group C and 2.3.4.4 Group D (Lee et al. 2016), and one case in Group B (WHO 2018a). To date (5 October 2018), a total of 21 H5N6 human cases have been reported in China with a high case fatality rate in diagnosed individuals (F.I.C. 2018; WHO 2018b). Ferret studies showed that although Asian ‘zoonotic’ HPAI H5N6 viruses replicated to high titres in the respiratory tract, there was no evidence for airborne transmissibility of these viruses (Herfst et al. 2018).

In this study, we attempt to determine the source of all eight influenza virus gene segments, and the estimates for the time to the most recent common ancestors (TMRCA) for the H5 HA and N6 NA gene segments to investigate when and where these viruses have evolved. We report the genetic relationships between the latest HPAI H5N6 viruses isolated from both European and Asian wild birds and poultry by using whole genome sequencing and characterize the emerging strains relative to other circulating viruses in the region.

## 2. Materials and methods

### 2.1 Wild bird surveillance and sequencing

Active surveillance programmes in wild birds, i.e. sampling of living healthy birds, are limited and often project-based, and resulting data are often not publicly available. Here, continuous active surveillance activities of influenza virus circulation in wild birds are reported for the Netherlands and the Republic of Georgia. Wild birds of various species were caught and sampled for virus detection as described previously (Munster and Fouchier 2009; Lewis et al. 2015; Venkatesh et al. 2018). Briefly, samples were tested for the presence of avian influenza A H5 viruses using a matrix gene specific and H5 HA gene specific real-time RT-PCR analysis followed by either Sanger sequencing, as described before (Munster and Fouchier 2009) (primer sequences are available upon request), or by MinION sequencing (Oxford Nanopore technologies). For MinION sequencing, RNA was extracted using the QIAamp Viral RNA Mini Kit (52904, Qiagen, UK) and a multi segment RT-PCR amplification was performed using the Superscript III high-fidelity RT-PCR Kit (12574-035, Invitrogen, USA) according to manufacturer’s instructions using the Opti1 primer set with influenza-specific universal primers complementary to the conserved 12–3 nucleotides at the end of all eight genomic segments: Opti1-F1 5′-GTTACGCGCCAGCAAAAGCAGG, Opti1-F2 5′ GTTACGCGCCAGCGAAAGCAGG, and Opti1-R1 5′ GTTACGCGCCAGTAGAAACAAGG. MinION sequencing was performed according to manufacturer’s instructions using the ID Native barcoding genomic DNA Kit (EXP-NBD103 and SQK-LSK108, Oxford Nanopore, UK). Raw sequence data were demultiplexed using Porechop (https://github.com/rrwick/Porechop), and a reference-based alignment was performed using CLC Genomics software, workbench 8 (CLC Bio), and the full genome Sanger sequence of A/Black-headed_Gull/Netherlands/29/2017 served as a reference (EPI_ISL_289714). Primers and adaptors were trimmed from the raw sequence data and the Phred score for alignment was set to eight and minimum require coverage was set to 100 reads per position. Discrepancies in the sequences (insertions or deletions) compared to close reference strains occurred only in homopolymeric regions, and were manually checked and resolved by incorporating an ‘N’ at these positions.

### 2.2 Strains of interest

To better understand the newly emerging HPAI H5N6 viruses from the 2.3.4.4 Group B lineage, viruses of the H5N6 subtype isolated in or after October 2017 were assigned as strains of interest (SOI). Depending on the continent of isolation, they were further classified as Asia-SOI or Europe-SOI (Table 1). Whole genome sequences for these viruses were obtained from public databases (GISAID and Genbank). European countries and non-European collaborators were asked to contribute any additional HPAI H5N6 and recent HxN6 sequence data via FLU-LAB-NET (European Union) or personal communication. HxN6 viruses were not considered SOI but included in the general set to test if any SOI N6s were closely related to them. In addition, the Animal and Plant Quarantine Agency from South Korea shared three whole genomes from HPAI H5N6 detections since November 2017 (GISAID accession numbers EPI_ISL_288436, EPI_ISL_288437, EPI_ISL_292349). Sequences of NA genes from recent (2016–17) LPAI HxN6 viruses from wild birds from the Netherlands, Belgium, Hungary, and Croatia were also obtained for the general set. Further, sequences of H5N6 viruses that were isolated from humans (HUM) in China in 2015, 2016, and 2017 (Table 1) were collected from GISAID (Supplementary Table S1) to test whether the genes of any of the emerging SOI were related to those zoonotic strains.

**Table 1.** Overview of our SOI of highly pathogenic avian influenza viruses H5N6 divided in Asia-SOI, Europe-SOI, and human H5N6 viruses.

Set	Strain name	Isolation date
	A/mute_swan/Shimane/3211A001/2017	05 November 2017
Asia-SOI	A/chicken/Vietnam/QuangBinh/BoTrach1113/2017	13 November 2017
	A/mallard/Korea/Jeju-H24/2017	17 November 2017
	A/duck/Korea/HD1/2017	17 November 2017
	A/spoonbill/Taiwan/DB645/2017	01 December 2017
	A/duck/Korea/H35/2017	10 December 2017
	A/mallard/Korea/H17-1825/2017	22 December 2017
	A/mandarin_duck/Korea/K17-1815/2017	22 December 2017
	A/mandarin_duck/Korea/K17-1817/2017	22 December 2017
	A/mandarin_duck/Korea/K17-1826/2017	22 December 2017
	A/mandarin_duck/Korea/K17-1828/2017	22 December 2017
	A/mandarin_duck/Korea/K17-1862/2017	23 December 2017
	A/mandarin_duck/Korea/K17-1866/2017	23 December 2017
	A/mandarin_duck/Korea/K17-1869/2017	23 December 2017
	A/mandarin_duck/Korea/K17-1873/2017	23 December 2017
	A/mandarin_duck/Korea/K17-1879/2017	23 December 2017
	A/mandarin_duck/Korea/K17-1881/2017	23 December 2017
	A/mandarin_duck/Korea/K17-1885/2017	23 December 2017
	A/mandarin_duck/Korea/K17-1887/2017	23 December 2017
	A/mandarin_duck/Korea/K17-1889/2017	23 December 2017
	A/mandarin_duck/Korea/K17-1891/2017	23 December 2017
	A/mandarin_duck/Korea/K17-1893/2017	23 December 2017
	A/mandarin_duck/Korea/K17-1894/2017	23 December 2017
	A/mandarin_duck/Korea/K17-1896/2017	23 December 2017
	A/Armenian_gull/Republic_of_Georgia/4/2017*	27 December 2017
	A/mandarin_duck/Korea/K18-3/2018	18 January 2018
	A/mallard/Republic of Georgia/1/2018*	28 January 2018
	A/duck/Vietnam/QuangBinh/QN530206/2018	06 February 2018
	A/jungle_crow/Hyogo/2803E011/2018	03 March 2018
	A/jungle_crow/Hyogo/2803E022/2018	06 March 2018
Europe-SOI	A/duck/Netherlands/17017236-001005/2017	07 December 2017
	A/duck/Netherlands/17017237-001-005/2017	07 December 2017
	A/tufted_duck/Netherlands/17017367-007/2017	09 December 2017
	A/mute_swan/Netherlands/17017367-012/2017	09 December 2017
	A/mute_swan/Netherlands/17017377-001/2017	09 December 2017
	A/great_black-backed_gull/Netherlands/1/2017*	18 December 2017
	A/black-headed_gull/Netherlands/29/2017*	18 December 2017
	A/common_pochard/Germany-BY/AR09-L02421/2017	28 December 2017
	A/mute_swan/England/SA21_180652/2018	02 January 2018
	A/Canada_goose/England/AV58_18OPpoolEP1/2018	05 January 2018
	A/pochard_duck/England/AVP_18_003254/2018	10 January 2018
	A/great_black-backed_gull/Netherlands/1/2018*	23 January 2018
	A/Eurasian wigeon/Netherlands/1/2018*	07 February 2018
	A/white-tailed_eagle/Denmark/3073-1w/2018	13 February 2018
	A/chicken/Netherlands/EMC-1/2018	26 February 2018
	A/domestic_duck/Netherlands/EMC-6/2018	13 March 2018
Human	A/Guangdong/ZQ874/2015	31 December 2015
	A/Shenzhen/1/2016	07 January 2016
	A/Anhui/33162/2016	28 April 2016
	A/Anhui/33163/2016	29 April 2016
	A/Hunan/55555/2016	18 November 2016
	A/Guangxi/55726/2016	24 November 2016
	A/Fujian-Sanyuan/21099/2017	25 December 2017

Viruses marked with an asterisk (*) were obtained and sequenced within the surveillance activities described in this manuscript.

### 2.3 Reassortment analyses—visualization of phylogenetic incongruence

To study the evolution and gene reassortment events of HPAI H5N6 viruses, a phylogenetic incongruence analysis was performed by aligning maximum likelihood (ML) trees for all influenza virus gene segments, except NA. Publicly available sequences of all eight segments of avian influenza viruses isolated between 1 January 2007 and 30 September 2017 were downloaded from Genbank. This formed a set of 61,842 sequences including all segments from all strains namely—HA: 8278, MP: 6464, NA: 7779, NP: 7382, NS: 6933, PA: 8346, PB1: 8410, PB2: 8250. This set of sequences was supplemented with the additional H5N6 and HxN6 sequences contributed by collaborators.

Sequences were first subjected to a quality control step where all duplicate sequences and sequences bearing duplicate IDs were removed. They were then separated into individual sequence datasets for each segment (PB2, PB1, PA, HA, NP, NA, MP, and NS). Python scripts obtained from https://github.com/ballesterus/Utensils.git were used to concatenate all segments from each strain. Only those viral strains which had sequences from all eight gene segments (4,000 strains) were selected. This concatenated sequence data was down-sampled using Cluster Database at High Identity with Tolerance (Li and Godzik 2006; Fu et al. 2012) to remove sequences with >95, 90, and 80 per cent sequence identity across the whole genome (all eight segments). An 80 per cent threshold was chosen as it provided the best compromise between retaining maximum possible diversity of sequences while retaining the ability to visualize. The resulting list of 141 strain names was used to extract sequences from those strains from each individual gene segment separately—resulting in the reduced whole-genome (WG) dataset for each segment.

Sequences of all segments from the SOI were also subjected to the same down-sampling procedure separately before being added to their respective WG-segment dataset. However, they were down-sampled to 99.5 per cent sequence identity (thirty-nine strains) to retain as much of the current diversity as possible. Sequences of all segments from all three strains that were isolated from humans (HUM) were also added, giving a total of 183 strains.

Final datasets were aligned using MAFFT v7.305b (Katoh and Standley 2013), and trimmed to only retain nucleotides from the startcodon ATG until the final STOP codon. We inferred ML phylogenetic trees for each gene segment using IQ-TREE, 1.5.5 (Nguyen et al. 2015) and ModelFinder (Kalyaanamoorthy et al. 2017) and obtained branch supports with Shimodaira-Hasegawa-like approximate Likelihood Ratio Test (1,000 replicates) and standard non-parametric bootstrap (100 replicates).

Backronymed adaptable lightweight tree import code (BALTIC) was used to compare the phylogenetic structure of the internal genes of the SOI. The phylogenetic position of each strain was traced, coloured according to the HA (Asia-SOI, Europe-SOI, H5N8 2016-17 clade, H5N6 Chinese, human H5N6 (HUM), other H5Nx, and LPAI) across unrooted ML trees for HA and all internal gene segments. Figures were generated by modifying scripts from a similar analysis (Bell and Bedford 2017) and editing in Adobe Illustrator. We selected a qualitative palette of colours using http://colorbrewer2.org/. The modified version of all scripts is available in a github repository (https://github.com/delfinut/phylogenetic-incongruence).

### 2.4 Analysis of the H5 and N6 genes

For the H5 analysis, 1,251 HA sequences of strains isolated between January 2016 and March 2018 were downloaded from GISAID (Supplementary Table S1). For the N6 tree, NA gene sequences of 1,680 strains of LPAI HxN6 viruses from Genbank, isolated between January 2007 and January 2018, were used. The Basic Local Alignment Search Tool (BLAST) was used to find the closest-related sequences to the HA and NA sequences from the SOI in the entire influenza virus resource database in FluDB (www.fludb.org). GISAID has the most up-to-date HPAI sequences—hence this database was used to acquire HPAI H5 HA sequences; Genbank has a better representation of HPAI and LPAI strains taken together, which is why this database was used for N6 NA sequences which may or may not be associated with HPAI HAs.

The individual H5 and N6 datasets described above were first subjected to a quality control step where all duplicate sequences and sequences bearing duplicate IDs were removed. These datasets were first analyzed with the SOI and HUM strains. Sequences were aligned using MAFFT v7.305b (Katoh and Standley 2013), and used in the FastTree program (Price, Dehal, and Arkin 2009, 2010) to generate an initial tree. Since both HA and NA of SOI viruses formed two monophyletic clades each (excluding all other strains), with robust support (>0.80), the downloaded sequences were then subjected to down sampling to a cut-off of 99.5 per cent identity before further analysis. SOI HA and NA sequences were also down-sampled to 99.9 per cent identity, respectively. All sequences from taxa which were found outside of the fully supported (100%) cluster with SOI were discarded. Ten further sequences were discarded after analysis of the H5 HA ML tree with tempest v1.5, which identified them as outliers and did not conform to clock-likeness as suggested by the authors in Rambaut et al. (2016). Similarly, nine further sequences were removed as outliers after analysis of the N6 NA ML tree with tempest v1.5. The H5 HA dataset included representatives from the HPAI clade 2.3.4.4 Group B (H5N8) 2016–17 clade, European and Asian HPAI clade 2.3.4.4 Groups B–D H5N6 viruses, other HPAI H5Nx viruses, as well as the separate lineage of the Groups C and D Chinese H5N6 strains, to form a new dataset of 153 sequences from which a final H5 ML tree was inferred using IQ-TREE. The N6 NA dataset included a total of 183 sequences from the H5N6 SOI as well as the most closely related sequences that were from LPAI viruses, primarily from wild birds. The final N6 ML tree was inferred from this dataset using IQ-TREE.

Bayesian phylogenetic trees were inferred using BEAST v1.10.1 (Suchard et al. 2018) to determine the time of emergence of SOI and the viruses they likely arose from. Path sampling/stepping stone sampling (Baele et al. 2012) was used to select the appropriate site substitution and clock models. BEAST calculates log marginal likelihood estimates (MLE) for each run from which log Bayes factors (BF) (which indicate support for one model over another) were calculated as the difference between the log MLEs. SRD06, i.e., the standard HKY site model with estimated base frequencies and gamma site heterogeneity with four gamma categories and two codon partitions (1 + 2, 3) was chosen over GTR with the empirical base frequencies and gamma categories with no codon partition (>180 log BF) (Shapiro, Rambaut, and Drummond 2006). An uncorrelated relaxed lognormal clock was used to allow for rate variation along different branches with Gaussian Markov random field (GMRF) Bayesian Skyride population prior (chosen over constant prior with >20 log BF) and random starting tree. All other priors were set to default. The Markov chain Monte Carlo (MCMC) was set to 70,000,000 generations. Two separate runs were performed to ensure convergence between runs. Log files were analyzed in Tracer v1.7.1 to determine convergence, and to check that ESS values were beyond threshold (>200). Log and trees files from both runs were combined using Log Combiner v 1.10.1. Tree annotator v1.10.1 was used to generate a maximum credibility tree (MCC) using 10 per cent burn in and median node heights. The MCC tree was then annotated to include posterior probability values and time scales and plotted in R v 3.5 using the ggtree package (Yu 2017).

## 3. Results

### 3.1 Wild bird surveillance and sequencing

Continuous active surveillance activities resulted in the screening of 2,769 wild birds in the Netherlands and 2,190 in the Republic of Georgia between 9 December 2017 and 30 June 2018. HPAI H5 viruses were detected in ten Eurasian Wigeons *(Anas penelope*, February 2018*)* in the Netherlands, and in a Mallard (*Anas platyrhynchos*, 28 January 2018), and an Armenian Gull (*Larus armenicus*, 27 December 2017) in the Republic of Georgia. In addition, during opportunistic sampling of a small number of dead birds (passive surveillance) the virus was detected in two Great Black-backed Gulls *(Larus marinus*, 18 December 2018 and 23 January 2018) and a Black-headed Gull (*Chroicocephalus ridibundus*, 18 December 2017) that were found dead in the Netherlands (Table 2). Full genome sequences were obtained from one of the HPAI H5N6 infected Dutch Eurasian Wigeons and the three dead gulls, and from both Georgian birds. All obtained sequences have been uploaded to GISAID with accession numbers EPI_ISL_289713, EPI_ISL_289714, EPI_ISL_302823, EPI_ISL_302824, EPI_ISL_303520, EPI_ISL_312376. During the mentioned surveillance period no additional LPAI HxN6 viruses were detected.

**Table 2.** Wild bird species sampled for virus detection during the 2017/18 outbreaks of highly pathogenic avian influenza H5N6 virus in the Netherlands (*n* = 2,816) and the Republic of Georgia (*n* = 2,190), 9 December 2017–30 June 2018.

Order	Family	Species	Republic of Georgia				The Netherlands							
			Alive without clinical signs				Alive without clinical signs				Found dead			
			No. birds sampled	No. birds AIV positive	No. birds H5 positive	Pathotype	No. birds sampled	No. birds AIV positive	No. birds H5 positive	Pathotype	No. birds sampled	No. birds AIV positive	No. birds H5 positive	Pathotype
*Accipitriformes*	Accipitridae	Hen harrier *(Circus cyaneus)*	0	0	0	NA	1	0	0	NA	0	0	0	NA
*Anseriformes*	Ducks	Common shelduck *(Tadorna tadorna)*	0	0	0	NA	2	0	0	NA	1	0	0	NA
		Eurasian teal *(Anas crecca)*	86	43	0	NA	236	54	2	2xLPAI	0	0	0	NA
		Eurasian wigeon *(Anas penelope)*	4	2	0	NA	332	64	32	10xHPAI, 3xLPAI, 19x.n.i	0	0	0	NA
		Gadwall *(Anas strepera)*	12	6	0	NA	114	33	2	1xLPAI, 1xn.i.	0	0	0	NA
		Garganey *(Spatula querquedula)*	32	16	0	NA	0	0	0	NA	0	0	0	NA
		Mallard *(Anas platyrhynchos)*	454	227	1	1xHPAI	2021	131	10	3xLPAI, 7xn.i.	2	0	0	NA
		Northern pintail *(Anas acuta)*	22	11	0	NA	2	0	0	NA	1	1	0	NA
		Northern shoveler *(Spatula clypeata)*	0	0	0	NA	3	0	0	NA	0	0	0	NA
		Ruddy shelduck *(Tadorna ferruginea)*	2	1	0	NA	0	0	0	NA	0	0	0	NA
		Tufted duck *(Aythya fuligula)*	8	4	0	NA	12	0	0	NA	0	0	0	NA
		Duck spp. *(Anas spp.)*	0	0	0	NA	21	1	0	NA	0	0	0	NA
	Geese	Greylag goose *(Anser anser)*	0	0	0	NA	0	0	0	NA	1	0	0	NA
		Pink-footed goose *(Anser brachyrhynchus)*	0	0	0	NA	1	0	0	NA	0	0	0	NA
	Swans	Bewick's swan *(Cygnus columbianus bewickii)*	0	0	0	NA	15	0	0	NA	0	0	0	NA
		Mute swan *(Cygnus olor)*	0	0	0	NA	0	0	0	NA	1	0	0	NA
*Charadriiformes*	Gulls	Armenian Gull *(Larus armenicus)*	234	117	1	1xHPAI	0	0	0	NA	0	0	0	NA
		Black-headed gull *(Chroicocephalus ridibundus)*	1036	518	0	NA	0	0	0	NA	8	2	1	1xHPAI
		Eurasian herring gull *(Larus argentatus)*	0	0	0	NA	0	0	0	NA	25	0	0	AN
		Great black-backed gull *(Larus marinus)*	0	0	0	NA	0	0	0	NA	6	2	3	2xHPAI, 1xn.i.
		Mediterranean Gull *(Ichthyaetus melanocephalus)*	2	1	0	NA	0	0	0	NA	0	0	0	NA
		Mew gull *(Larus canus)*	2	1	0	NA	1	0	0	NA	1	0	0	NA
		Slender-billed Gull *(Chroicocephalus genei)*	4	2	0	NA	0	0	0	NA	0	0	0	NA
		Yellow-legged Gull *(Larus michahellis)*	282	141	0	NA	0	0	0	NA	0	0	0	NA
	Waders	Common snipe *(Gallinago gallinago)*	0	0	0	NA	2	0	0	NA	0	0	0	NA
		Northern lapwing *(Vanellus vanellus)*	0	0	0	NA	2	0	0	NA	0	0	0	NA
*Gaviiformes*		Arctic Loon *(Gavia arctica)*	2	1	0	NA	0	0	0	NA	0	0	0	NA
*Gruiformes*	Rails	Common moorhen *(Gallinula chloropus)*	0	0	0	NA	2	0	0	NA	1	0	0	NA
		Water rail *(Rallus aquaticus)*	0	0	0	NA	2	0	0	NA	0	0	0	NA
*Podicipediformes*		Eared Grebe *(Podiceps nigricollis)*	4	2	0	NA	0	0	0	NA	0	0	0	NA
		Great crested grebe *(Podiceps cristatus)*	2	1	0	NA	0	0	0	NA	0	0	0	NA
*Procellariiformes*		Levantine Shearwater *(Puffinus yelkouan)*	2	1	0	NA	0	0	0	NA	0	0	0	NA
Total			2,190	1,095	2		2,769	283	46		47	5	4	

NA, not applicable; n.i., not identifiable.

### 3.2 Phylogenetic analyses of the HA segment

**Figure 2.**
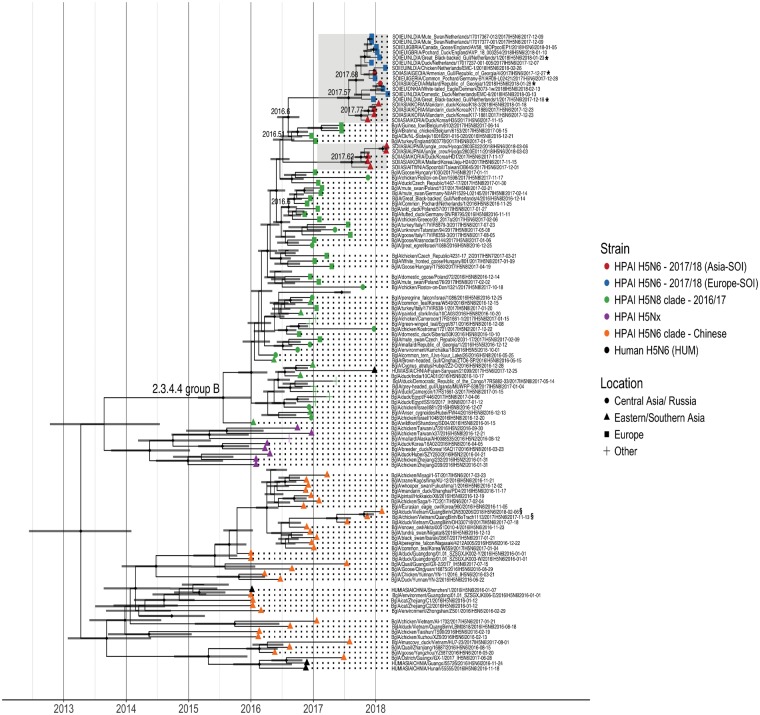
BEAST trees from viral sequences of HA (H5) gene sequences isolated from avian hosts between January 2016 and April 2018 with the addition of four H5N6 HA genes isolated from humans and seven co-circulating HPAI H5N2/H5N8 viruses (purple). Tip symbols are coloured according to the HA origin displaying our European-SOI (blue), Asian-SOI (red), the Chinese HPAI H5N6 viruses (orange) with the Chinese human-derived viruses (black), the 2016–17 HPAI H5N8 viruses (green), and other HPAI H5Nx viruses (purple). Tip symbols depict the location: Central Asia/Russia (●), Eastern/Southern Asia (▲), Europe (■), and other (+). Presence of node symbol (♦) indicates posterior *P* > 0.85. The numbers above the nodes represent TMRCA, the grey bars display the accompanying 95 per cent confidence interval. Viruses marked with an asterisk (*) were obtained and sequenced within the surveillance activities described in this manuscript.

Previously, H5 clades have been somewhat geographically restricted with only intermittent incursion of Asian lineage viruses into Europe. However, here phylogenetic clustering of the Europe-derived H5N6 SOI was observed with H5N6 strains derived from Asia, such as those isolated from ducks in South Korea, a Black-faced Spoonbill *(Platalea minor)* in Taiwan, and a Mute Swan *(Cygnus olor)* in Japan in November/December 2017 (Asia-SOI). These latest H5N6 strains were also phylogenetically similar to recent European/Russian HPAI H5N8 viruses rather than Asian-derived H5N6 viruses such as the Chinese H5N6s that have been associated with zoonotic infections (Supplementary Fig. S1A). The recent, and to date, only, human H5N6 Group B strain (WHO 2018) (A/Fujian-Sanyuan/21099/2017(H5N6)) clustered closer to, but was still distinct from the recent European H5N6 strains (Fig. 2, Supplementary Fig. S1A).

With the exception of two Vietnamese viruses (A/duck/Vietnam/QuangBinh/QN530206/2018 and A/chicken/Vietnam/QuangBinh/BoTrach1113/2017, Fig. 2 marked with §) that clustered together with the Chinese HPAI H5N6 viruses, both the European-SOI and Asian-SOI sets of H5N6 viruses had an estimated common ancestor that circulated in early July 2016 (95% confidence interval April–September 2016). The H5N6 virus isolated from a single poultry outbreak in Greece during the 2016–17 HPAI H5N8 epizootic (A/chicken/Greece/39_2017a/2017) clustered with other 2016–17 European H5N8 viruses, suggesting this strain was not ancestral to the SOI (Fig. 2, Supplementary Fig. S1A).

However, additional heterogeneity was observed within the SOI. Phylogenetically clustering within the Europe-SOI were two Asia-SOI strains; these two Asia-SOI strains were isolated in the Republic of Georgia. The Republic of Georgia is located in western Central Asia, on the eastern side of the Black Sea, and here, these viruses showed a closer phylogenetic relationship with European strains than with Asian strains. The Europe-SOI and the Georgian strains had a common ancestor that circulated in early September 2017 (95% confidence interval July–October 2017), suggesting that there were likely multiple genetically distinct H5 segments, whose subsequent diffusion within the wild bird population was not uniform across Eurasia. Similar finer grain heterogeneity was observed with the four recent Asian strains, isolated from South Korea in late 2017 and early 2018. These four South Korean strains were phylogenetically closer to the European-SOI but distinct from other recent Asian H5N6 2.3.4.4 Group B viruses, isolated from birds in Japan, South Korea, and Taiwan in late 2017/early 2018. The Korean and European viruses shared a common ancestor that circulated in July 2017 (95% confidence interval May–September 2017). This suggests that potentially two separate HA reassortment events led to heterogenous H5N6 viruses circulating within Eurasia from July 2016 and co-circulating in South Korea in late 2017 (Fig. 2).

### 3.3 Phylogenetic analyses of the NA segment

**Figure 3.**
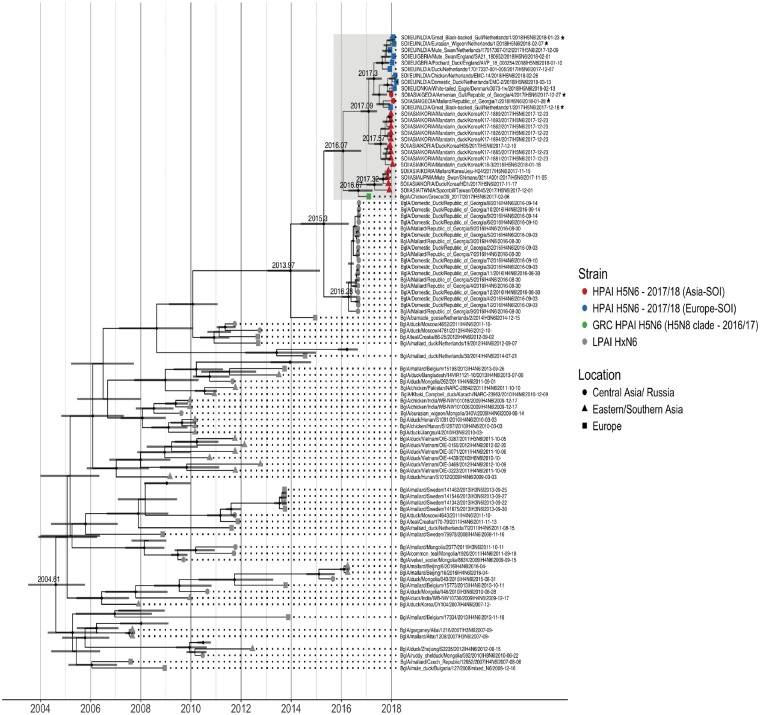
BEAST trees from viral sequences of NA (N6) gene sequences isolated from avian hosts between January 2007 and April 2018. Tip symbols are coloured according to the HA origin with recent Asian (red) and European (blue) HPAI H5N6 viruses, the early 2017 Greek (GRC) HPAI H5N6 (green), and (non-H5) HxN6 (grey) viruses. Symbols depict the location: Central Asia/Russia (●), Eastern/Southern Asia (▲) and Europe (■). Presence of node symbol (♦) indicates posterior *P* > 0.85. The numbers above the nodes represent TMRCA, the grey bars display the accompanying 95 per cent confidence interval. Viruses marked with an asterisk (*) were obtained and sequenced within the surveillance activities described in this manuscript.

Since the H5N6 HA arose from the recent H5N8 strains, attempts were made to trace the reassortment event or events that potentially led to the emergence of these H5N6 viruses. All N6 NA segments derived from LPAI and HPAI viruses from 2007 to January 2018 were collected and combined with the SOI to trace the origin of N6. The closest genetically related N6 to all HPAI H5N6 strains appeared to be LPAI H4N6 strains from the Republic of Georgia in 2016 (Fig. 3, Supplementary Fig. S1B).

BEAST analysis estimated that a common ancestor for all N6 genes related to HPAI H5 viruses that circulated in early January 2016 (albeit with a large 95% confidence interval between April 2015 and October 2016). The N6 segments of the Korean and European/Georgian viruses seemed to have diverged right at the beginning of 2017. The most recent common ancestor for the N6 related to only the European/Georgian HPAI H5N6 viruses that circulated in April 2017 (95% confidence interval December 2016–August 2017). The N6 from the Greek H5N6 strain from February 2017 phylogenetically clustered alone and diverged from its closest relatives, some of the Asia-SOI, in late summer 2016, indicating that this Greek virus was an unrelated reassortment event that did not continue to circulate and was not ancestral to the currently circulating N6 genes related to the HPAI H5 viruses (Fig. 3).

### 3.4 Full genome phylogeny

**Figure 4.**
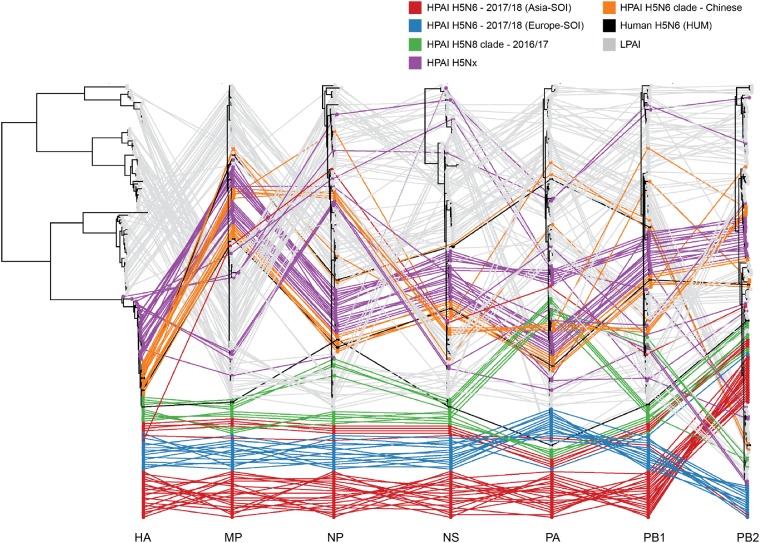
Phylogenetic incongruence analysis. Maximum likelihood trees for the HA segment and all internal genes MP, NP, NS, PA, PB1, and PB2 from equivalent strains were connected across the trees. Tips and connecting lines are coloured according to HA clade.

BALTIC was used to compare the phylogenetic structure of the internal genes of the SOI compared to other HPAI H5 and LPAI viruses. Supplementary Fig. S1A–H shows the ML trees for all eight gene segments of the SOI together with a down-sampled set of all avian viruses isolated between 2007 and 2018. To visualize incongruence, the phylogenetic position of each sequence (coloured according to the origin of its HA) was traced across all seven trees (Fig. 4). The eighth gene segment, NA, is excluded from this because not all viruses were N6 viruses, hence this tree would rather show the obvious genetic differences between different NA subtypes, than tracing the N6.

The HA of the recent European/Asian SOI clustered within the 2016/17 HPAI H5N8 cluster, and the NA segments were most closely related to LPAI N6s, as described above (Figs 2 and 3, Supplementary Fig. S1A and B). A/Mallard/Republic_of_Georgia/1/2018 (H5N6) showed a closer relationship to recent European viruses than to Asian viruses for all eight gene segments (Fig. 4, Supplementary Fig. S1). The MP, NP, NS, and PB1 segments of recent Asian- and European-SOI were related to respective internal genes from 2016 to 2017 clade 2.3.4.4 (H5N8) strains (Supplementary Fig. S1). In contrast, although A/Armenian_Gull/Republic_of_Georgia/4/2017 (H5N6) was closely related to the European H5N6 viruses for the HA and NA segments, all of its internal genes clustered with those of LPAI and other HPAI H5 viruses, indicating extensive reassortment (Figs 2–4, Supplementary Fig. S1).

For PA, SOI from Europe and the Republic of Georgia (except A/Great_Black-backed_Gull/Netherlands/1/2017) phylogenetically clustered with LPAI H7, H3, and H4 viruses from Bangladesh, the Netherlands, and the Republic of Georgia from 2014 to 2016. Phylogenetically distinct from this PA group, the A/Great_Black-backed_Gull/Netherlands/1/2017 and all Asia-SOI (except the H5N6 virus from the Republic of Georgia) clustered together with 2016/17 Russian/European HPAI H5N8 viruses. These different PA clusters likely represent separate reassortment events. (Supplementary Fig. S1F).

Phylogeny based on the PB2 gene resulted in similar clustering patterns as compared with PA. However, the closest-related PB2 genes to the Europe-SOI/Republic of Georgia cluster arose from viruses that circulated in 2014–5 in domestic birds in Europe. In contrast, PB2 segments of the Asia-SOI and A/Great_Black-backed_Gull/Netherlands/1/2017 clustered with PB2 segments from 2016 to 2017 Russian/European H5N8 (Supplementary Fig. S1H). None of the H5N6 SOI gene segments were associated with respective segments found in or near the Chinese Groups C and D viruses (Fig. 4). However, the recent Group B human H5N6 virus (A/Fujian-Sanyuan/21099/2017(H5N6)) isolated in China in 2017 was more similar to HPAI H5N8 clade viruses with a PA segment that is closely related to both HPAI H5N8 clade and HPAI H5N6 European clade viruses.

## 4. Discussion

New reassortant HPAI clade 2.3.4.4 Group B H5N6 viruses have been detected in both wild birds and poultry in several European and Asian countries from December 2017 onwards. Phylogenetic analyses of these viruses showed that although they were related to the 2016–17 European HPAI H5N8 viruses, they were not the result of continued circulation of the single HPAI clade 2.3.4.4 Group B H5N6 virus that was detected in Greece in early 2017 during the 2016–17 clade 2.3.4.4 Group B H5N8 European outbreak. Based on these results, we conclude that these European and some recent Asian HPAI H5N6 viruses are new reassortant viruses of HPAI H5 clade 2.3.4.4 Group B viruses with LPAI virus segments likely derived from wild birds rather than from LPAI viruses in poultry or from introductions of the (zoonotic) Asian HPAI H5N6 viruses that have been frequently detected in Southeast Asia since 2014. The common ancestors of the HA and NA gene segments of the recent European HPAI H5N6 viruses were estimated to have circulated in early September 2017 (confidence interval: July–October) and April 2017 (confidence interval: December 2016–August 2017), respectively. Previously published time estimates for the most recent common ancestors for these HPAI H5N6 viruses detected in the Netherlands (Beerens et al. 2018) and Europe/Korea (Kwon et al. 2018) were January–September 2016 and January–October 2016 for HA, and December 2014–July 2016 and September 2015 (confidence interval: August 2014–August 2016) for NA, respectively. These estimates are different from these new estimates and with much broader confidence intervals. This can be explained by the more extensive dataset in the present study, including both a representative set of circulating HPAI H5 viruses and a large number of LPAI viruses that enabled us to narrow the time estimates of when reassortment events with other viruses may have occurred.

Although wild birds are generally not considered as a long-term maintenance reservoir for HPAI viruses, bird diversity, density, the aggregation of birds from different geographical areas, and the high density of immune-naïve birds on breeding sites can all contribute to both temporary HPAI virus maintenance and to the potential generation of novel reassortants with LPAI gene segments. Previous phylogenetic studies have shown that both HPAI clade 2.3.4.4 H5N8 viruses that caused outbreaks in Europe in 2014–15 and 2016–17 originated in Southeast Asia and subsequently spread to Europe (Lycett et al. 2016; Pohlmann et al. 2017), with diffusion mediated by wild birds (Lycett et al. 2016). However, in contrast to previous epizootics where an HPAI H5 progenitor was derived from poultry and diffused in wild birds, it is more plausible that these recent H5N6 viruses evolved during 2017 by reassortment of a European HPAI H5N8 virus and wild host reservoir LPAI viruses, within the wild bird reservoir itself, given the more restricted gene pool in poultry. We therefore hypothesize that these reassortment events occurred either during 2017 on the Palaearctic breeding grounds where birds from Europe and Asia gather when a large number of hatch-year birds entered the wild bird population, or just after breeding, when naïve hatch-year birds and adults dispersed from the breeding grounds to aggregate in large numbers, particularly to moult (Newton 2011).

Previously, differences in mortality rates were found in HPAI H5 epizootics mediated by wild birds (Alarcon et al. 2018). Here, the combination of enzootic presence of LPAI viruses, a large immunologically heterogenous population, with possible pre-existing immunity to clade 2.3.4.4 Group B viruses from the previous HPAI waves, and the start of migration might have enabled multiple novel reassortants to emerge and to disperse both east and west to the wintering grounds, resulting in co-circulation of reassortants of different genotypes in both Europe and East Asia. The timing of the first detection of the new H5N6 viruses was later (mid-December) (Beerens et al. 2018) compared with both H5N8 outbreaks (early November (Harder et al. 2015) and late October (OIE 2016), respectively) but the variation in detection was likely within normal ecological variation of wild bird movements and population dynamics. Possible ecological and population-based explanations for such outbreak timing variation include the presence of pre-existing immunity to clade 2.3.4.4 Group B viruses possibly leading to less severe infections, the potential for partial immunity to facilitate co-infection and recovery rather than mortality, with reassortment and onwards transmission resulting from these altered population factors. Additionally partial or pre-existing immunity might lead to a relative reduction in disease burden and the number of infected birds, fewer infected wild birds without obvious increased mortality, differences in climatic factors between years that influenced the dispersal of wild birds (Vandegrift et al. 2010) and thus relative prevalence, or the alteration of the structure and size of the wild bird population by the previous incursion (Kleyheeg et al. 2017) and the relative proportion of susceptible birds in the population differed across years.

Although some HPAI H5 viruses were associated with mortality in wild birds (Chen et al. 2005; Keawcharoen et al. 2008; Kleyheeg et al. 2017; Pohlmann et al. 2017), serological evidence and the detections of HPAI H5 viruses in clinically healthy birds (Verhagen et al. 2015; Poen et al. 2016, 2018) indicate that infections can be non-lethal, enabling the spread of these viruses over longer distances with flight. To the authors’ knowledge all previous reports on HPAI H5N6 detections in Europe have been from either diseased or dead birds. Here, the detection of HPAI H5N6 virus from a clinically healthy Armenian Gull (December 2017) and a Mallard (January 2018) in the Republic of Georgia, and in clinically healthy Eurasian Wigeons in February 2017 in the Netherlands are reported. Wild birds are considered to be a temporary spill-over host for HPAI viruses that originate from poultry, and HPAI viruses are generally considered to be unable to become endemic in the wild bird population. The present results and those previously published (Beerens et al. 2018; Kwon et al. 2018) indicate that the recent HPAI clade 2.3.4.4 Group B H5N6 viruses were reassortants from several wild bird viruses, and time estimates for these reassortment events suggested that reassortment happened in wild birds without the involvement of poultry. In addition, HPAI H5N6 viruses have been detected in wild birds in Europe over the course of 2018, although there were no detections in commercial poultry after March 2018, nor in backyard poultry between March and September 2018 (OIE 2018). These viruses may indeed have adapted to wild birds by causing non-lethal infections, and thereby enabled themselves to be maintained in the wild bird population. To date the precise wild bird species involved in the long-distance dispersal of these viruses are still unknown, but with expanding genetic heterogeneity in both wild birds and poultry the risk of diffusion of HPAI variants among geographic areas is an ever-present threat.

Clade 2.3.4.4 Groups A and B H5N8 viruses have not been associated with human infections, and pathogenesis and transmission studies in ferrets showed that these viruses are not of current concern for human health (Richard et al. 2015; Grund et al. 2018; Lee et al. 2018b). In contrast, clade 2.3.4.4 Groups B–D H5N6 viruses have been reported to cause human infections in China (Aveyard et al. 2016; Pan et al. 2016), and show a high genetic variability. Based on the present phylogenetic results, all eight gene segments of the currently circulating European and most Asian H5N6 viruses, with the exception of some Chinese and Vietnamese viruses, clustered with either HPAI H5N8 or LPAI viruses, and showed a clear separation from the human clade 2.3.4.4 Groups C and D viruses. Recently the WHO reported a human infection with a clade 2.3.4.4 Group B virus (A/Fujian-Sanyuan/21099/2017(H5N6)) (WHO 2018a) though this is to date, a single isolation. However, the evolutionary dynamics characterized in this study highlight the potential for rapid virus evolution, alterations in host susceptibility even within an H5 subclade and demonstrate the necessity to continually assess the risk of emerging variants to both animal and human health. It is also important to understand the underlying principles and drivers that enable global HPAI virus migration through both active and passive, ecologically-targeted and longitudinal surveillance in wild birds and poultry.

## Supplementary Material

Supplementary Data
