# Supplementary material for: Co-circulation of genetically distinct highly pathogenic avian influenza A clade 2.3.4.4 (H5N6) viruses in wild waterfowl and poultry in Europe and East Asia, 2017–18
Source: Virus Evol. 2019 Apr 22;5(1):vez004. doi: 10.1093/ve/vez004 (PMC6476160; doi:10.1093/ve/vez004)
Supplement: Supplementary Data [file vez004_supp.zip › Supplemental_Table_1.docx]

**Supplemental Table 1.** Acknowledgements of authors, and originating and submitting laboratories providing the sequences to Gisaid used for phylogenetic analysis.

| **Isolate_Id** | **Isolate_Name** | **Collection_Date** | **Originating_Lab** | **Submitting_Lab** | **Authors** |
| --- | --- | --- | --- | --- | --- |
| EPI_ISL_255211 | A/Harris_hawk/Hungary/2762b/2017 | 2017-01-24 | Central Agricultural Office Veterinary Diagnostic Directorate | Central Agricultural Office Veterinary Diagnostic Directorate | Dan,A |
| EPI_ISL_290313 | A/peregrine falcon/Gifu/2102A008/2017 | 2017-02-24 | Not listed | Other Database Import | Soda,K;Usui,T;Ito,H;Ozaki,H;Yamaguchi,T;Ito,T |
| EPI_ISL_256521 | A/peregrine falcon/Niigata/12/2017 | 2017-01-01 | Not listed | Hokkaido University | MasatoshiOkamatsu |
| EPI_ISL_255206 | A/Peregrine_falcon/Hungary/4882/2017 | 2017-02-07 | Central Agricultural Office Veterinary Diagnostic Directorate | Central Agricultural Office Veterinary Diagnostic Directorate | Dan,A |
| EPI_ISL_301049 | A/domestic_goose/Poland/270/2017 | 2017-03-06 | Not listed | National Veterinary Research Institut Poland, PIWet-PIB | Swieton,E;Smietanka,K |
| EPI_ISL_300699 | A/domestic_goose/Poland/69/2017 | 2017-01-27 | National Veterinary Research Institut Poland, PIWet-PIB | National Veterinary Research Institut Poland, PIWet-PIB | Swieton,E;Smietanka,K |
| EPI_ISL_295793 | A/goose/Vietnam/HU8-1596/2017 | 2017-08-21 | Not listed | Other Database Import | Nguyen,LT;Chu,DH;Okamatsu,M;Matsuno,K;Sakoda,Y;Jizou,M |
| EPI_ISL_285655 | A/Geese/South_Africa/S2017/08_0558_P1/2017 | 2017-08-29 | Western Cape Provincial Veterinary Laboratory | National Institute of Communicable Diseases | Treurnicht,FK |
| EPI_ISL_285648 | A/Geese/South_Africa/S2017/09_0055_P1/2017 | 2017-09-04 | Western Cape Provincial Veterinary Laboratory | National Institute of Communicable Diseases | Treurnicht,FK |
| EPI_ISL_285647 | A/Geese/South_Africa/S2017/08_0520_46/2017 | 2017-08-28 | Western Cape Provincial Veterinary Laboratory | National Institute of Communicable Diseases | Treurnicht,FK |
| EPI_ISL_285609 | A/Geese/South_Africa/S2017/08_0558_P2/2017 | 2017-08-29 | Western Cape Provincial Veterinary Laboratory | National Institute of Communicable Diseases | Treurnicht,FK |
| EPI_ISL_285602 | A/Geese/South_Africa/S2017/09_0065_P2/2017 | 2017-09-04 | Western Cape Provincial Veterinary Laboratory | National Institute of Communicable Diseases | Treurnicht,FK |
| EPI_ISL_285601 | A/Geese/South_Africa/S2017/09_0065_P1/2017 | 2017-09-04 | Western Cape Provincial Veterinary Laboratory | National Institute of Communicable Diseases | Treurnicht,FK |
| EPI_ISL_282394 | A/goose/Yangzhou/YZ587/2016 | 2016-05-20 | Not listed | Other Database Import | Sun,W |
| EPI_ISL_282143 | A/goose/Italy/17VIR6358-3/2017 | 2017-08-05 | Istituto Zooprofilattico Sperimentale Delle Venezie | Istituto Zooprofilattico Sperimentale Delle Venezie | Zecchin,B;Fusaro,A;Zamperin,G;Schivo,A;Salviato,A;Marciano,S;Ormelli,S;Terregino,C;Monne,I |
| EPI_ISL_272740 | A/goose/Guangdong/QY01/2016 | 2016-10-21 | Not listed | Other Database Import | Ren,T;Xiang,B;Xiao,C |
| EPI_ISL_271713 | A/Goose/Hungary/64909/2016 | 2016-12-14 | National Food Chain Safety Office Veterinary Diagnostic Directorate Laboratory for Molecular Biology | Central Agricultural Office Veterinary Diagnostic Directorate | Dan,A |
| EPI_ISL_271712 | A/Goose/Hungary/63743/2016 | 2016-12-11 | National Food Chain Safety Office Veterinary Diagnostic Directorate Laboratory for Molecular Biology | Central Agricultural Office Veterinary Diagnostic Directorate | Dan,A |
| EPI_ISL_271709 | A/Goose/Hungary/59763/2016 | 2016-11-27 | National Food Chain Safety Office Veterinary Diagnostic Directorate Laboratory for Molecular Biology | Central Agricultural Office Veterinary Diagnostic Directorate | Dan,A |
| EPI_ISL_271708 | A/Goose/Hungary/17985/2017 | 2017-04-20 | National Food Chain Safety Office Veterinary Diagnostic Directorate Laboratory for Molecular Biology | Central Agricultural Office Veterinary Diagnostic Directorate | Dan,A |
| EPI_ISL_268655 | A/Go/NL-Roggebotsluis/16014462-010/2016 | 2016-11-17 | Wageningen Bioveterinary Research | Wageningen Bioveterinary Research | Beerens,N;Heutink,R;Harders,F;Verschuren-Pritz,S;Bossers,A;Koch,G;Bergervoet,S |
| EPI_ISL_255398 | A/Goose/Hungary/59712/2016 | 2016-11-26 | Central Agricultural Office Veterinary Diagnostic Directorate | Central Agricultural Office Veterinary Diagnostic Directorate | Dan,A |
| EPI_ISL_249682 | A/domestic goose/Germany-BY/R677/2017 | 2017-01-25 | Not listed | Friedrich-Loeffler-Institut | Starick,E |
| EPI_ISL_240679 | A/graylag goose/Kazakhstan/KR/2016 | 2016-08-15 | Research Institute of Experimental and Clinical Medicine | Research Institute of Experimental and Clinical Medicine | Sharshov,K;Kurskaya,O;Sobolev,I;Leonov,S;Yushkov,Y;Alekseev,A;Alikina,T;Kabilov,M;Shestopalov,A |
| EPI_ISL_256514 | A/white-fronted goose/Miyagi/1/2016 | 2016-11-21 | Not listed | Hokkaido University | MasatoshiOkamatsu |
| EPI_ISL_268977 | A/goose/Czech Republic/1998-17_1/2017 (H5N8) | 2017-02-09 | State Veterinary Institute Prague | State Veterinary Institute Prague | Nagy,A |
| EPI_ISL_268976 | A/goose/Czech Republic/1954-17/2017 (H5N8) | 2017-02-08 | State Veterinary Institute Prague | State Veterinary Institute Prague | Nagy,A |
| EPI_ISL_268971 | A/bronze turkey/Czech Republic/1755-17_1/2017 (H5N8) | 2017-02-05 | State Veterinary Institute Prague | State Veterinary Institute Prague | Nagy,A |
| EPI_ISL_268932 | A/goose/Czech Republic/197-17/2017 (H5N8) | 2017-01-02 | State Veterinary Institute Prague | State Veterinary Institute Prague | Nagy,A |
| EPI_ISL_262059 | A/greylag goose/Germany-NI/AR703-L02138/2017 | 2017-01-25 | Not listed | Friedrich-Loeffler-Institut | Pohlmann,A |
| EPI_ISL_262058 | A/greylag goose/Germany-NI/AR1395-L02144/2017 | 2017-02-02 | Not listed | Friedrich-Loeffler-Institut | Pohlmann,A |
| EPI_ISL_262056 | A/greylag goose/Germany-NI/AR11353-L02142/2016 | 2016-12-27 | Not listed | Friedrich-Loeffler-Institut | Pohlmann,A |
| EPI_ISL_255212 | A/Greylag_goose/Hungary/320/2017 | 2017-01-04 | Central Agricultural Office Veterinary Diagnostic Directorate | Central Agricultural Office Veterinary Diagnostic Directorate | Dan,A |
| EPI_ISL_255196 | A/Greylag_goose/Hungary/1941/2017 | 2017-01-18 | Central Agricultural Office Veterinary Diagnostic Directorate | Central Agricultural Office Veterinary Diagnostic Directorate | Dan,A |
| EPI_ISL_247722 | A/goose/Krasnodar/3144/2017 | 2017-01-06 | Not listed | State Research Center of Virology and Biotechnology (VECTOR) | Susloparov,I;Goncharova,N;Kolosova,N;Marchenko,V;Ryzhikov,A |
| EPI_ISL_243695 | A/greylag goose/Croatia/33/2017 | 2017-01-18 | Not listed | Croatian Veterinary Institute | Savić,Vladimir |
| EPI_ISL_295492 | A/Anser cygnoides/Hubei/FW44/2016 | 2016-12-13 | Not listed | Other Database Import | Ma,L;Zhao,L;Wang,R;Chen,Q |
| EPI_ISL_224740 | A/Bar-headed Goose/Qinghai/BTY18-LU/2016 | 2016-05-15 | State Key Laboratory of Virology and Wuhan Institute of Virology, Chinese Academy of Sciences | Wuhan Institute of Virology | Chen,J |
| EPI_ISL_224739 | A/Bar-headed Goose/Qinghai/BTY18-B/2016 | 2016-05-15 | State Key Laboratory of Virology and Wuhan Institute of Virology, Chinese Academy of Sciences | Wuhan Institute of Virology | Chen,J |
| EPI_ISL_224738 | A/Bar-headed Goose/Qinghai/BTY17-LU/2016 | 2016-05-15 | State Key Laboratory of Virology and Wuhan Institute of Virology, Chinese Academy of Sciences | Wuhan Institute of Virology | Chen,J |
| EPI_ISL_224736 | A/Bar-headed Goose/Qinghai/BTY16-LU/2016 | 2016-05-14 | State Key Laboratory of Virology and Wuhan Institute of Virology, Chinese Academy of Sciences | Wuhan Institute of Virology | Chen,J |
| EPI_ISL_224734 | A/Bar-headed Goose/Qinghai/BTY15-LU/2016 | 2016-05-14 | State Key Laboratory of Virology and Wuhan Institute of Virology, Chinese Academy of Sciences | Wuhan Institute of Virology | Chen,J |
| EPI_ISL_224731 | A/Bar-headed Goose/Qinghai/BTY14-B/2016 | 2016-05-14 | State Key Laboratory of Virology and Wuhan Institute of Virology, Chinese Academy of Sciences | Wuhan Institute of Virology | Chen,J |
| EPI_ISL_224729 | A/Bar-headed Goose/Qinghai/BTY13-B/2016 | 2016-05-14 | State Key Laboratory of Virology and Wuhan Institute of Virology, Chinese Academy of Sciences | Wuhan Institute of Virology | Chen,J |
| EPI_ISL_224728 | A/Bar-headed Goose/Qinghai/BTY12-LU/2016 | 2016-05-13 | State Key Laboratory of Virology and Wuhan Institute of Virology, Chinese Academy of Sciences | Wuhan Institute of Virology | Chen,J |
| EPI_ISL_224726 | A/Bar-headed Goose/Qinghai/BTY11-LU/2016 | 2016-05-09 | State Key Laboratory of Virology and Wuhan Institute of Virology, Chinese Academy of Sciences | Wuhan Institute of Virology | Chen,J |
| EPI_ISL_224725 | A/Bar-headed Goose/Qinghai/BTY11-B/2016 | 2016-05-09 | State Key Laboratory of Virology and Wuhan Institute of Virology, Chinese Academy of Sciences | Wuhan Institute of Virology | Chen,J |
| EPI_ISL_224724 | A/Bar-headed Goose/Qinghai/BTY10-LU/2016 | 2016-05-09 | State Key Laboratory of Virology and Wuhan Institute of Virology, Chinese Academy of Sciences | Wuhan Institute of Virology | Chen,J |
| EPI_ISL_224721 | A/Bar-headed Goose/Qinghai/BTY9-B/2016 | 2016-05-09 | State Key Laboratory of Virology and Wuhan Institute of Virology, Chinese Academy of Sciences | Wuhan Institute of Virology | Chen,J |
| EPI_ISL_224720 | A/Bar-headed Goose/Qinghai/BTY8-LU/2016 | 2016-05-10 | State Key Laboratory of Virology and Wuhan Institute of Virology, Chinese Academy of Sciences | Wuhan Institute of Virology | Chen,J |
| EPI_ISL_224719 | A/Bar-headed Goose/Qinghai/BTY8-B/2016 | 2016-05-10 | State Key Laboratory of Virology and Wuhan Institute of Virology, Chinese Academy of Sciences | Wuhan Institute of Virology | Chen,J |
| EPI_ISL_224718 | A/Bar-headed Goose/Qinghai/BTY7-LU2/2016 | 2016-05-11 | State Key Laboratory of Virology and Wuhan Institute of Virology, Chinese Academy of Sciences | Wuhan Institute of Virology | Chen,J |
| EPI_ISL_224713 | A/Bar-headed Goose/Qinghai/BTY5-B/2016 | 2016-05-12 | State Key Laboratory of Virology and Wuhan Institute of Virology, Chinese Academy of Sciences | Wuhan Institute of Virology | Chen,J |
| EPI_ISL_224712 | A/Bar-headed Goose/Qinghai/BTY4-LU/2016 | 2016-05-12 | State Key Laboratory of Virology and Wuhan Institute of Virology, Chinese Academy of Sciences | Wuhan Institute of Virology | Chen,J |
| EPI_ISL_224711 | A/Bar-headed Goose/Qinghai/BTY4-B/2016 | 2016-05-12 | State Key Laboratory of Virology and Wuhan Institute of Virology, Chinese Academy of Sciences | Wuhan Institute of Virology | Chen,J |
| EPI_ISL_224710 | A/Bar-headed Goose/Qinghai/BTY3-LU/2016 | 2016-05-12 | State Key Laboratory of Virology and Wuhan Institute of Virology, Chinese Academy of Sciences | Wuhan Institute of Virology | Chen,J |
| EPI_ISL_224709 | A/Bar-headed Goose/Qinghai/BTY3-B/2016 | 2016-05-12 | State Key Laboratory of Virology and Wuhan Institute of Virology, Chinese Academy of Sciences | Wuhan Institute of Virology | Chen,J |
| EPI_ISL_224708 | A/Bar-headed Goose/Qinghai/BTY2-LU/2016 | 2016-05-12 | State Key Laboratory of Virology and Wuhan Institute of Virology, Chinese Academy of Sciences | Wuhan Institute of Virology | Chen,J |
| EPI_ISL_224707 | A/Bar-headed Goose/Qinghai/BTY2-B/2016 | 2016-05-12 | State Key Laboratory of Virology and Wuhan Institute of Virology, Chinese Academy of Sciences | Wuhan Institute of Virology | Chen,J |
| EPI_ISL_224704 | A/Bar-headed Goose/Qinghai/BTY1-B/2016 | 2016-05-09 | State Key Laboratory of Virology and Wuhan Institute of Virology, Chinese Academy of Sciences | Wuhan Institute of Virology | Chen,J |
| EPI_ISL_309198 | A/Guinea fowl/Belgium/6102/2017 | 2017-06-14 | Not listed | Other Database Import | VanBorm,S;Vandenbussche,F;Mathijs,E;Lambrecht,B;Steensels,M |
| EPI_ISL_285652 | A/Guineafowl/South_Africa/S2017/08_0243_P2/2017 | 2017-08-16 | Western Cape Provincial Veterinary Laboratory | National Institute of Communicable Diseases | Treurnicht,FK |
| EPI_ISL_285617 | A/Guineafowl/South_Africa/S2017/08_0243_P1/2017 | 2017-08-16 | Western Cape Provincial Veterinary Laboratory | National Institute of Communicable Diseases | Treurnicht,FK |
| EPI_ISL_285610 | A/Guineafowl/South_Africa/S2017/08_0274_P1/2017 | 2017-08-16 | Western Cape Provincial Veterinary Laboratory | National Institute of Communicable Diseases | Treurnicht,FK |
| EPI_ISL_268659 | A/Gull1/NL-Marker Wadden/16014466-011/2016 | 2016-11-17 | Wageningen Bioveterinary Research | Wageningen Bioveterinary Research | Beerens,N;Heutink,R;Harders,F;Verschuren-Pritz,S;Bossers,A;Koch,G;Bergervoet,S |
| EPI_ISL_268658 | A/Gull/NL-Marker Wadden/16014466-020/2016 | 2016-11-17 | Wageningen Bioveterinary Research | Wageningen Bioveterinary Research | Beerens,N;Heutink,R;Harders,F;Verschuren-Pritz,S;Bossers,A;Koch,G;Bergervoet,S |
| EPI_ISL_240613 | A/black headed gull/Ibaraki/265T/2016 | 2016-12-20 | National Institute of Animal Health | National Institute of Animal Health | Uchida,Y |
| EPI_ISL_240608 | A/black headed gull/Ibaraki/258T/2016 | 2016-12-18 | National Institute of Animal Health | National Institute of Animal Health | Uchida,Y |
| EPI_ISL_240000 | A/black headed gull/Ibaraki/253T/2016 | 2016-12-18 | National Institute of Animal Health | National Institute of Animal Health | Mine,J |
| EPI_ISL_239999 | A/black headed gull/Ibaraki/235T/2016 | 2016-12-15 | National Institute of Animal Health | National Institute of Animal Health | Mine,J |
| EPI_ISL_269601 | A/Eurasian_Herring_Gull/Netherlands/2/2016 | 2016-12-20 | Erasmus Medical Center | Erasmus Medical Center | Poen,MJ;VanDerJeugd,HP;Vuong,O;Scheuer,RD;Kleyheeg,E;Bestebroer,TM;Begeman,L;vandenBrand,JMA;Kuiken,T;Fouchier,RAM |
| EPI_ISL_224751 | A/Brown-headed Gull/Qinghai/ZTO6-MU/2016 | 2016-05-15 | State Key Laboratory of Virology and Wuhan Institute of Virology, Chinese Academy of Sciences | Wuhan Institute of Virology | Chen,J |
| EPI_ISL_224749 | A/Brown-headed Gull/Qinghai/ZTO6-B/2016 | 2016-05-15 | State Key Laboratory of Virology and Wuhan Institute of Virology, Chinese Academy of Sciences | Wuhan Institute of Virology | Chen,J |
| EPI_ISL_224748 | A/Brown-headed Gull/Qinghai/ZTO5-K/2016 | 2016-05-14 | State Key Laboratory of Virology and Wuhan Institute of Virology, Chinese Academy of Sciences | Wuhan Institute of Virology | Chen,J |
| EPI_ISL_224747 | A/Brown-headed Gull/Qinghai/ZTO5-B/2016 | 2016-05-14 | State Key Laboratory of Virology and Wuhan Institute of Virology, Chinese Academy of Sciences | Wuhan Institute of Virology | Chen,J |
| EPI_ISL_224746 | A/Brown-headed Gull/Qinghai/ZTO4-B/2016 | 2016-05-11 | State Key Laboratory of Virology and Wuhan Institute of Virology, Chinese Academy of Sciences | Wuhan Institute of Virology | Chen,J |
| EPI_ISL_224745 | A/Brown-headed Gull/Qinghai/ZTO3-LU/2016 | 2016-05-12 | State Key Laboratory of Virology and Wuhan Institute of Virology, Chinese Academy of Sciences | Wuhan Institute of Virology | Chen,J |
| EPI_ISL_255910 | A/Mew Gull/Netherlands/1/2016 | 2016-11-23 | Erasmus Medical Center | Erasmus Medical Center | Poen,MJ;VanDerJeugd,HP;Vuong,O;Scheuer,RD;Kleyheeg,E;Lexmond,P;Eggink,WD;Müskens,GJDM;Bestebroer,TM;OudeMunnink,BB;Phan,VTM;Cotten,ML;Koopmans,MPG;Kuiken,T;Fouchier,RAM |
| EPI_ISL_268661 | A/L-bl-ba-gull/NL-Sovon/16014324-014/2016 | 2016-11-16 | Wageningen Bioveterinary Research | Wageningen Bioveterinary Research | Beerens,N;Heutink,R;Harders,F;Verschuren-Pritz,S;Bossers,A;Koch,G;Bergervoet,S |
| EPI_ISL_277417 | A/glaucous-winged gull/Southcentral Alaska/16MB03648/2016 | 2016-09-26 | Not listed | Other Database Import | Pickett,B;Tan,G;Fedorova,N;Puri,V;Shrivastava,S;Amedeo,P;Isom,R;Hu,L;Christensen,J;Novotny,M;Durbin,A;Rocchi,I;Williams,T;Hill,N;Bao,Y;Sanders,R;Zhdanov,S;Kiryutin,B;Lipman,DJ;Tatusova,T;Hatcher,E;Runstadler,J |
| EPI_ISL_224752 | A/Great Black-headed Gull/Qinghai/YO1-B/2016 | 2016-05-15 | State Key Laboratory of Virology and Wuhan Institute of Virology, Chinese Academy of Sciences | Wuhan Institute of Virology | Chen,J |
| EPI_ISL_302823 | A/Great black-backed gull/Netherlands/1/2018 | 2018-01-23 | Erasmus Medical Center | Erasmus Medical Center | Poen,MJ;Bestebroer,TM;Vuong,O;Scheuer,RD;Kelder,L;Fouchier,RAM |
| EPI_ISL_289713 | A/Great Black-backed Gull/Netherlands/1/2017 | 2017-12-18 | Erasmus Medical Center | Erasmus Medical Center | Poen,MJ;Bestebroer,TM;Kelder,L;Scheuer,RD;Koopmans,MPG;VanDerJeugd,HP;Fouchier,RAM |
| EPI_ISL_269697 | A/Great_Black-backed_Gull/Netherlands/2/2016 | 2016-11-23 | Erasmus Medical Center | Erasmus Medical Center | Poen,MJ;VanDerJeugd,HP;Vuong,O;Scheuer,RD;Kleyheeg,E;Bestebroer,TM;Begeman,L;vandenBrand,JMA;Kuiken,T;Fouchier,RAM |
| EPI_ISL_269599 | A/Great_Black-backed_Gull/Netherlands/4/2016 | 2016-12-14 | Erasmus Medical Center | Erasmus Medical Center | Poen,MJ;VanDerJeugd,HP;Vuong,O;Scheuer,RD;Kleyheeg,E;Bestebroer,TM;Begeman,L;vandenBrand,JMA;Kuiken,T;Fouchier,RAM |
| EPI_ISL_269597 | A/Great_Black-backed_Gull/Netherlands/1/2016 | 2016-11-23 | Erasmus Medical Center | Erasmus Medical Center | Poen,MJ;VanDerJeugd,HP;Vuong,O;Scheuer,RD;Kleyheeg,E;Bestebroer,TM;Begeman,L;vandenBrand,JMA;Kuiken,T;Fouchier,RAM |
| EPI_ISL_295752 | A/black-headed gull/Hyogo/2801E009/2017 | 2017-01-18 | Not listed | Other Database Import | Soda,K;Usui,T;Ito,H;Ozaki,H;Yamaguchi,T;Ito,T |
| EPI_ISL_295702 | A/black-headed gull/Ibaraki/0803-194/2016 | 2016-12-05 | Not listed | Other Database Import | Soda,K;Usui,T;Ito,H;Ozaki,H;Yamaguchi,T;Ito,T |
| EPI_ISL_268656 | A/Gr_bk_bd_gull/NL-Slootdorp/16014102-005/2016 | 2016-11-11 | Wageningen Bioveterinary Research | Wageningen Bioveterinary Research | Beerens,N;Heutink,R;Harders,F;Verschuren-Pritz,S;Bossers,A;Koch,G;Bergervoet,S |
| EPI_ISL_256301 | A/environment/Kamchatka/18/2016 | 2016-10-01 | Not listed | State Research Center of Virology and Biotechnology (VECTOR) | Susloparov,I;Goncharova,N;Kolosova,N;Marchenko,V;Ryzhikov,A |
| EPI_ISL_255209 | A/Common_tern/Hungary/8187/2017 | 2017-02-28 | Central Agricultural Office Veterinary Diagnostic Directorate | Central Agricultural Office Veterinary Diagnostic Directorate | Dan,A |
| EPI_ISL_285918 | A/Ostrich/South_Africa/S2017/08_0362_P8_34/2017 | 2017-08-22 | Western Cape Provincial Veterinary Laboratory | National Institute of Communicable Diseases | Treurnicht,FK |
| EPI_ISL_285650 | A/Ostrich/South_Africa/S2017/08_0046_AF/2017 | 2017-08-03 | Western Cape Provincial Veterinary Laboratory | National Institute of Communicable Diseases | Treurnicht,FK |
| EPI_ISL_285616 | A/Ostrich/South_Africa/S2017/08_0161_P8/2017 | 2017-08-11 | Western Cape Provincial Veterinary Laboratory | National Institute of Communicable Diseases | Treurnicht,FK |
| EPI_ISL_285604 | A/Ostrich/South_Africa/S2017/08_0046_P3/2017 | 2017-08-03 | Western Cape Provincial Veterinary Laboratory | National Institute of Communicable Diseases | Treurnicht,FK |
| EPI_ISL_285488 | A/Ostrich/South_Africa/S2017/08_0047_P7/2017 | 2017-08-03 | Western Cape Provincial Veterinary Laboratory | National Institute of Communicable Diseases | Treurnicht,FK |
| EPI_ISL_285480 | A/Ostrich/South_Africa/S2017/08_0362_P10/2017 | 2017-08-22 | Western Cape Provincial Veterinary Laboratory | National Institute of Communicable Diseases | Treurnicht,FK |
| EPI_ISL_284684 | A/Ostrich/South_Africa/S2017/08_0268_P9/2017 | 2017-08-16 | Western Cape Provincial Veterinary Laboratory | National Institute of Communicable Diseases | Treurnicht,FK |
| EPI_ISL_284683 | A/Ostrich/South_Africa/S2017/08_0268_P2/2017 | 2017-08-16 | Western Cape Provincial Veterinary Laboratory | National Institute of Communicable Diseases | Treurnicht,FK |
| EPI_ISL_284197 | A/Ostrich/South_Africa/S2017/08_0046_P2/2017 | 2017-08-03 | Western Cape Provincial Veterinary Laboratory | National Institute of Communicable Diseases | Treurnicht,FK |
| EPI_ISL_266798 | A/quail/Deliserdang/01160025/2016 | 2016-01-22 | Not listed | Other Database Import | Wibawa,H;Poermadjaja,B;Mulyawan,H;Dharmawan,R;Mahawan,T;Hutagaol,NM;Miswati,Y;Srihanto,EA;Hartawan,DHW;Hendrawati,F;Riyadi,A;Deswarni;TriHarsono,A;Hartaningsih,N;Azhar,M;Stegemen,A;McGrane,J;Rasa,FST |
| EPI_ISL_266537 | A/peacock/Belgium/1017/2017 | 2017-02-08 | Not listed | Other Database Import | Steensels,M;Lambrecht,B;Vandenbussche,F;VanBorm,S |
| EPI_ISL_301078 | A/mute_swan/Poland/109/2017 | 2017-02-12 | Not listed | National Veterinary Research Institut Poland, PIWet-PIB | Swieton,E;Smietanka,K |
| EPI_ISL_301072 | A/swan/Poland/107/2017 | 2017-02-12 | Not listed | National Veterinary Research Institut Poland, PIWet-PIB | Swieton,E;Smietanka,K |
| EPI_ISL_301069 | A/swan/Poland/88/2017 | 2017-02-04 | Not listed | National Veterinary Research Institut Poland, PIWet-PIB | Swieton,E;Smietanka,K |
| EPI_ISL_301067 | A/mute_swan/Poland/76/2017 | 2017-02-02 | Not listed | National Veterinary Research Institut Poland, PIWet-PIB | Swieton,E;Smietanka,K |
| EPI_ISL_301063 | A/mute_swan/Poland/72/2017 | 2017-02-01 | Not listed | National Veterinary Research Institut Poland, PIWet-PIB | Swieton,E;Smietanka,K |
| EPI_ISL_301057 | A/mute_swan/Poland/68/2017 | 2017-01-31 | Not listed | National Veterinary Research Institut Poland, PIWet-PIB | Swieton,E;Smietanka,K |
| EPI_ISL_301056 | A/swan/Poland/56/2017 | 2017-01-27 | Not listed | National Veterinary Research Institut Poland, PIWet-PIB | Swieton,E;Smietanka,K |
| EPI_ISL_301055 | A/mute_swan/Poland/54/2017 | 2017-01-26 | Not listed | National Veterinary Research Institut Poland, PIWet-PIB | Swieton,E;Smietanka,K |
| EPI_ISL_301054 | A/swan/Poland/49/2017 | 2017-01-26 | Not listed | National Veterinary Research Institut Poland, PIWet-PIB | Swieton,E;Smietanka,K |
| EPI_ISL_301052 | A/mute_swan/Poland/30/2017 | 2017-01-19 | Not listed | National Veterinary Research Institut Poland, PIWet-PIB | Swieton,E;Smietanka,K |
| EPI_ISL_301051 | A/swan/Poland/23/2017 | 2017-01-17 | Not listed | National Veterinary Research Institut Poland, PIWet-PIB | Swieton,E;Smietanka,K |
| EPI_ISL_301050 | A/mute_swan/Poland/12/2017 | 2017-01-12 | Not listed | National Veterinary Research Institut Poland, PIWet-PIB | Swieton,E;Smietanka,K |
| EPI_ISL_300747 | A/mute_swan/Poland/137/2017 | 2017-02-21 | Not listed | National Veterinary Research Institut Poland, PIWet-PIB | Swieton,E;Smietanka,K |
| EPI_ISL_300744 | A/swan/Poland/32/2017 | 2017-01-19 | Not listed | National Veterinary Research Institut Poland, PIWet-PIB | Swieton,E;Smietanka,K |
| EPI_ISL_260798 | A/tundra swan/Niigata/11/2016 | 2016-12-19 | Not listed | Hokkaido University | MasatoshiOkamatsu |
| EPI_ISL_260794 | A/tundra swan/Niigata/5/2016 | 2016-12-11 | Not listed | Hokkaido University | MasatoshiOkamatsu |
| EPI_ISL_260793 | A/tundra swan/Niigata/3/2016 | 2016-12-10 | Not listed | Hokkaido University | MasatoshiOkamatsu |
| EPI_ISL_260792 | A/tundra swan/Niigata/2/2016 | 2016-12-09 | Not listed | Hokkaido University | MasatoshiOkamatsu |
| EPI_ISL_260791 | A/whooper swan/Aomori/8/2016 | 2016-12-19 | Not listed | Hokkaido University | MasatoshiOkamatsu |
| EPI_ISL_260790 | A/whooper swan/Aomori/7/2016 | 2016-12-13 | Not listed | Hokkaido University | MasatoshiOkamatsu |
| EPI_ISL_260787 | A/whooper swan/Aomori/3/2016 | 2016-12-02 | Not listed | Hokkaido University | MasatoshiOkamatsu |
| EPI_ISL_260784 | A/whooper swan/Hokkaido/X5/2016 | 2016-12-08 | Not listed | Hokkaido University | MasatoshiOkamatsu |
| EPI_ISL_260781 | A/whooper swan/Iwate/20/2017 | 2017-03-08 | Not listed | Hokkaido University | MasatoshiOkamatsu |
| EPI_ISL_260778 | A/whooper swan/Iwate/16/2017 | 2017-01-08 | Not listed | Hokkaido University | MasatoshiOkamatsu |
| EPI_ISL_260777 | A/swan/Iwate/15/2017 | 2017-01-05 | Not listed | Hokkaido University | MasatoshiOkamatsu |
| EPI_ISL_260775 | A/whooper swan/Iwate/12/2016 | 2016-12-22 | Not listed | Hokkaido University | MasatoshiOkamatsu |
| EPI_ISL_260772 | A/whooper swan/Iwate/6/2016 | 2016-12-20 | Not listed | Hokkaido University | MasatoshiOkamatsu |
| EPI_ISL_260770 | A/tundra swan/Iwate/3/2016 | 2016-12-13 | Not listed | Hokkaido University | MasatoshiOkamatsu |
| EPI_ISL_260767 | A/mute swan/Ibaraki/6/2016 | 2016-12-14 | Not listed | Hokkaido University | MasatoshiOkamatsu |
| EPI_ISL_260765 | A/mute swan/Ibaraki/4/2016 | 2016-12-14 | Not listed | Hokkaido University | MasatoshiOkamatsu |
| EPI_ISL_260764 | A/mute swan/Ibaraki/3/2016 | 2016-12-14 | Not listed | Hokkaido University | MasatoshiOkamatsu |
| EPI_ISL_257416 | A/black_swan/Ibaraki/28-446T/2017 | 2017-01-02 | National Institute of Animal Health | National Institute of Animal Health | Saito,T;Takemae,N |
| EPI_ISL_247405 | A/mute swan/Ibaraki/220T/2016 | 2016-12-14 | National Institute of Animal Health | National Institute of Animal Health | Takehiko,S |
| EPI_ISL_247394 | A/whooper swan/Ibaraki/188C/2016 | 2016-11-29 | National Institute of Animal Health | National Institute of Animal Health | Takehiko,S |
| EPI_ISL_247391 | A/mute swan/Ibaraki/217T/2016 | 2016-12-13 | National Institute of Animal Health | National Institute of Animal Health | Takehiko,S |
| EPI_ISL_247390 | A/mute swan/Ibaraki/218T/2016 | 2016-12-14 | National Institute of Animal Health | National Institute of Animal Health | Takehiko,S |
| EPI_ISL_247389 | A/mute swan/Ibaraki/223T/2016 | 2016-12-14 | National Institute of Animal Health | National Institute of Animal Health | Takehiko,S |
| EPI_ISL_247387 | A/mute swan/Ibaraki/202T/2016 | 2016-12-08 | National Institute of Animal Health | National Institute of Animal Health | Takehiko,S |
| EPI_ISL_247386 | A/mute swan/Ibaraki/214T/2016 | 2016-12-13 | National Institute of Animal Health | National Institute of Animal Health | Takehiko,S |
| EPI_ISL_247376 | A/mute swan/Ibaraki/208T/2016 | 2016-12-10 | National Institute of Animal Health | National Institute of Animal Health | Takehiko,S |
| EPI_ISL_247375 | A/mute swan/Ibaraki/222C/2016 | 2016-12-14 | National Institute of Animal Health | National Institute of Animal Health | Takehiko,S |
| EPI_ISL_240626 | A/black swan/Ibaraki/259T/2016 | 2016-12-19 | National Institute of Animal Health | National Institute of Animal Health | Uchida,Y |
| EPI_ISL_240619 | A/black swan/Ibaraki/272C/2016 | 2016-12-21 | National Institute of Animal Health | National Institute of Animal Health | Uchida,Y |
| EPI_ISL_240617 | A/mute swan/Kyoto/2T/2016 | 2016-12-22 | National Institute of Animal Health | National Institute of Animal Health | Uchida,Y |
| EPI_ISL_240615 | A/mute swan/Ibaraki/255C/2016 | 2016-12-18 | National Institute of Animal Health | National Institute of Animal Health | Uchida,Y |
| EPI_ISL_240611 | A/mute swan/Ibaraki/269T/2016 | 2016-12-21 | National Institute of Animal Health | National Institute of Animal Health | Uchida,Y |
| EPI_ISL_240605 | A/mute swan/Kyoto/5T/2016 | 2016-12-22 | National Institute of Animal Health | National Institute of Animal Health | Uchida,U |
| EPI_ISL_240604 | A/mute swan/Kyoto/6T/2016 | 2016-12-22 | National Institute of Animal Health | National Institute of Animal Health | Uchida,U |
| EPI_ISL_240601 | A/mute swan/Kyoto/8T/2016 | 2016-12-22 | National Institute of Animal Health | National Institute of Animal Health | Uchida,Y |
| EPI_ISL_240009 | A/mute swan/Ibaraki/242T/2016 | 2016-12-16 | National Institute of Animal Health | National Institute of Animal Health | Mine,J |
| EPI_ISL_240008 | A/mute swan/Ibaraki/249T/2016 | 2016-12-17 | National Institute of Animal Health | National Institute of Animal Health | Mine,J |
| EPI_ISL_240007 | A/mute swan/Ibaraki/240C/2016 | 2016-12-16 | National Institute of Animal Health | National Institute of Animal Health | Mine,J |
| EPI_ISL_240005 | A/mute swan/Ibaraki/232C/2016 | 2016-12-14 | National Institute of Animal Health | National Institute of Animal Health | Mine,J |
| EPI_ISL_240004 | A/mute swan/Ibaraki/245C/2016 | 2016-12-16 | National Institute of Animal Health | National Institute of Animal Health | Mine,J |
| EPI_ISL_240002 | A/mute swan/Ibaraki/236T/2016 | 2016-12-15 | National Institute of Animal Health | National Institute of Animal Health | Mine,J |
| EPI_ISL_240001 | A/mute swan/Ibaraki/239C/2016 | 2016-12-16 | National Institute of Animal Health | National Institute of Animal Health | Mine,J |
| EPI_ISL_295494 | A/Cygnus atratus/Hubei/HF-1/2016 | 2016-12-20 | Not listed | Other Database Import | Ma,L;Zhao,L;Wang,R;Chen,Q |
| EPI_ISL_294414 | A/black swan/Aichi/2312T003/2016 | 2016-12-06 | Not listed | Other Database Import | Soda,K;Usui,T;Ito,H;Ozaki,H;Yamaguchi,T;Ito,T |
| EPI_ISL_294412 | A/black swan/Aichi/2312T001/2016 | 2016-11-29 | Not listed | Other Database Import | Soda,K;Usui,T;Ito,H;Ozaki,H;Yamaguchi,T;Ito,T |
| EPI_ISL_261687 | A/black swan/Germany-BW/R1364/2017 | 2017-02-12 | Not listed | Friedrich-Loeffler-Institut | Starick,E |
| EPI_ISL_243059 | A/black swan/Akita/2/2016 | 2016-11-19 | Not listed | Other Database Import | Okamatsu,M;Hiono,T;Matsuno,K;Kida,H;Sakoda,Y |
| EPI_ISL_243058 | A/black swan/Akita/1/2016 | 2016-11-19 | Not listed | Other Database Import | Okamatsu,M;Hiono,T;Matsuno,K;Kida,H;Sakoda,Y |
| EPI_ISL_300663 | A/tundra swan/Niigata/5112007/2016 | 2016-12-06 | Not listed | Other Database Import | Soda,K;Usui,T;Ito,H;Ozaki,H;Yamaguchi,T;Ito,T |
| EPI_ISL_300662 | A/tundra swan/Niigata/5112006/2016 | 2016-12-04 | Not listed | Other Database Import | Soda,K;Usui,T;Ito,H;Ozaki,H;Yamaguchi,T;Ito,T |
| EPI_ISL_300661 | A/tundra swan/Niigata/5112004/2016 | 2016-12-02 | Not listed | Other Database Import | Soda,K;Usui,T;Ito,H;Ozaki,H;Yamaguchi,T;Ito,T |
| EPI_ISL_300660 | A/tundra swan/Niigata/1511C003/2016 | 2016-11-27 | Not listed | Other Database Import | Soda,K;Usui,T;Ito,H;Ozaki,H;Yamaguchi,T;Ito,T |
| EPI_ISL_256520 | A/tundra swan/Niigata/8/2016 | 2016-12-13 | Not listed | Hokkaido University | MasatoshiOkamatsu |
| EPI_ISL_256519 | A/tundra swan/Niigata/4/2016 | 2016-12-11 | Not listed | Hokkaido University | MasatoshiOkamatsu |
| EPI_ISL_256518 | A/tundra swan/Niigata/1/2016 | 2016-12-08 | Not listed | Hokkaido University | MasatoshiOkamatsu |
| EPI_ISL_256510 | A/tundra swan/Iwate/9/2016 | 2016-12-22 | Not listed | Hokkaido University | MasatoshiOkamatsu |
| EPI_ISL_295701 | A/whooper swan/Ibaraki/28-309/2016 | 2016-11-29 | Not listed | Other Database Import | Soda,K;Usui,T;Ito,H;Ozaki,H;Yamaguchi,T;Ito,T |
| EPI_ISL_290311 | A/whooper swan/Niigata/5112008/2016 | 2016-12-06 | Not listed | Other Database Import | Soda,K;Usui,T;Ito,H;Ozaki,H;Yamaguchi,T;Ito,T |
| EPI_ISL_256522 | A/whooper swan/Niigata/13/2017 | 2017-01-27 | Not listed | Hokkaido University | MasatoshiOkamatsu |
| EPI_ISL_256517 | A/whooper swan/Fukushima/3/2016 | 2016-12-14 | Not listed | Hokkaido University | MasatoshiOkamatsu |
| EPI_ISL_256516 | A/whooper swan/Fukushima/1/2016 | 2016-12-02 | Not listed | Hokkaido University | MasatoshiOkamatsu |
| EPI_ISL_256513 | A/whooper swan/Iwate/17/2016 | 2017-01-16 | Not listed | Hokkaido University | MasatoshiOkamatsu |
| EPI_ISL_256511 | A/whooper swan/Iwate/11/2016 | 2016-12-21 | Not listed | Hokkaido University | MasatoshiOkamatsu |
| EPI_ISL_256509 | A/whooper swan/Iwate/8/2016 | 2016-12-22 | Not listed | Hokkaido University | MasatoshiOkamatsu |
| EPI_ISL_256507 | A/whooper swan/Iwate/1/2016 | 2016-11-23 | Not listed | Hokkaido University | MasatoshiOkamatsu |
| EPI_ISL_244539 | A/whooper swan/Korea/Gangjin 49_1/2016 | 2016-11-20 | Not listed | Other Database Import | Jeong,J;Woo,C |
| EPI_ISL_244538 | A/whooper swan/Korea/Gangjin 49_2/2016 | 2016-11-20 | Not listed | Other Database Import | Jeong,J;Woo,C |
| EPI_ISL_300664 | A/mute swan/Hyogo/2801ITM015/2017 | 2017-01-23 | Not listed | Other Database Import | Soda,K;Usui,T;Ito,H;Ozaki,H;Yamaguchi,T;Ito,T |
| EPI_ISL_295705 | A/mute swan/Ibaraki/0803-214/2016 | 2016-12-13 | Not listed | Other Database Import | Soda,K;Usui,T;Ito,H;Ozaki,H;Yamaguchi,T;Ito,T |
| EPI_ISL_295703 | A/mute swan/Ibaraki/0803-201/2016 | 2016-12-08 | Not listed | Other Database Import | Soda,K;Usui,T;Ito,H;Ozaki,H;Yamaguchi,T;Ito,T |
| EPI_ISL_293504 | A/mute swan/Hyogo/2801ITM001/2017 | 2017-01-13 | Not listed | Other Database Import | Soda,K;Usui,T;Ito,H;Ozaki,H;Yamaguchi,T;Ito,T |
| EPI_ISL_292223 | A/mute_swan/England/AVP_18_001986/2017 | 2017-12-31 | Animal and Plant Health Agency (APHA) | Animal and Plant Health Agency (APHA) | Seekings,James;Ellis,Richard;Brookes,SharonM;Reid,Scott;Essen,Stephen;Lewis,Nicola;Brown,IanH |
| EPI_ISL_290309 | A/mute swan/Hyogo/2801ITM014/2017 | 2017-01-18 | Not listed | Other Database Import | Soda,K;Usui,T;Ito,H;Ozaki,H;Murase,T;Yamaguchi,T;Ito,T |
| EPI_ISL_290308 | A/mute swan/Hyogo/2801ITM013/2017 | 2017-01-18 | Not listed | Other Database Import | Soda,K;Usui,T;Ito,H;Ozaki,H;Murase,T;Yamaguchi,T;Ito,T |
| EPI_ISL_290307 | A/mute swan/Hyogo/2801ITM012/2017 | 2017-01-18 | Not listed | Other Database Import | Soda,K;Usui,T;Ito,H;Ozaki,H;Murase,T;Yamaguchi,T;Ito,T |
| EPI_ISL_290301 | A/mute swan/Hyogo/2801ITM008/2017 | 2017-01-17 | Not listed | Other Database Import | Soda,K;Usui,T;Ito,H;Ozaki,H;Murase,T;Yamaguchi,T;Ito,T |
| EPI_ISL_290297 | A/mute swan/Hyogo/2801ITM004/2017 | 2017-01-14 | Not listed | Other Database Import | Soda,K;Usui,T;Ito,H;Ozaki,H;Murase,T;Yamaguchi,T;Ito,T |
| EPI_ISL_290296 | A/mute swan/Hyogo/2801ITM003/2017 | 2017-01-14 | Not listed | Other Database Import | Soda,K;Usui,T;Ito,H;Ozaki,H;Murase,T;Yamaguchi,T;Ito,T |
| EPI_ISL_288410 | A/Mute Swan/Netherlands/17017377-001/2017 | 2017-12-09 | Wageningen Bioveterinary Research | Wageningen Bioveterinary Research | Beerens,N;Heutink,R;Harders,F;Verschuren-Pritz,S;Bossers,A;Koch,G;Bergervoet,S |
| EPI_ISL_288409 | A/Mute Swan/Netherlands/17017367-012/2017 | 2017-12-09 | Wageningen Bioveterinary Research | Wageningen Bioveterinary Research | Beerens,N;Heutink,R;Harders,F;Verschuren-Pritz,S;Bossers,A;Koch,G;Bergervoet,S |
| EPI_ISL_282141 | A/swan/Italy/17VIR7064-1/2017 | 2017-08-25 | Istituto Zooprofilattico Sperimentale Delle Venezie | Istituto Zooprofilattico Sperimentale Delle Venezie | Zecchin,B;Fusaro,A;Zamperin,G;Schivo,A;Salviato,A;Marciano,S;Ormelli,S;Terregino,C;Monne,I |
| EPI_ISL_274858 | A/mute swan/Kaliningrad/132/2017 | 2017-02-13 | Not listed | State Research Center of Virology and Biotechnology (VECTOR) | Susloparov,I;Goncharova,N;Kolosova,N;Marchenko,V;Ryzhikov,A |
| EPI_ISL_268978 | A/mute swan/Czech Republic/2008-17_1/2017 (H5N8) | 2017-02-07 | State Veterinary Institute Prague | State Veterinary Institute Prague | Nagy,A |
| EPI_ISL_268973 | A/mute swan/Czech Republic/1848-17_1/2017 (H5N8) | 2017-02-06 | State Veterinary Institute Prague | State Veterinary Institute Prague | Nagy,A |
| EPI_ISL_268970 | A/mute swan/Czech Republic/1691-17/2017 (H5N8) | 2017-02-02 | State Veterinary Institute Prague | State Veterinary Institute Prague | Nagy,A |
| EPI_ISL_268958 | A/mute swan/Czech Republic/1519-17/2017 (H5N8) | 2017-02-02 | State Veterinary Institute Prague | State Veterinary Institute Prague | Nagy,A |
| EPI_ISL_268956 | A/mute swan/Czech Republic/1461-17/2017 (H5N8) | 2017-01-29 | State Veterinary Institute Prague | State Veterinary Institute Prague | Nagy,A |
| EPI_ISL_268951 | A/mute swan/Czech Republic/1330-17_1/2017 (H5N8) | 2017-01-20 | State Veterinary Institute Prague | State Veterinary Institute Prague | Nagy,A |
| EPI_ISL_268950 | A/mute swan/Czech Republic/1296-17_1/2017 (H5N8) | 2017-01-24 | State Veterinary Institute Prague | State Veterinary Institute Prague | Nagy,A |
| EPI_ISL_268949 | A/mute swan/Czech Republic/1227-17/2017 (H5N8) | 2017-01-22 | State Veterinary Institute Prague | State Veterinary Institute Prague | Nagy,A |
| EPI_ISL_268944 | A/mute swan/Czech Republic/1170-17_2/2017 (H5N8) | 2017-01-22 | State Veterinary Institute Prague | State Veterinary Institute Prague | Nagy,A |
| EPI_ISL_268943 | A/mute swan/Czech Republic/1156-17/2017 (H5N8) | 2017-01-22 | State Veterinary Institute Prague | State Veterinary Institute Prague | Nagy,A |
| EPI_ISL_268942 | A/mute swan/Czech Republic/1155-17/2017 (H5N8) | 2017-01-22 | State Veterinary Institute Prague | State Veterinary Institute Prague | Nagy,A |
| EPI_ISL_268940 | A/mute swan/Czech Republic/1058-17/2017 (H5N8) | 2017-01-18 | State Veterinary Institute Prague | State Veterinary Institute Prague | Nagy,A |
| EPI_ISL_268936 | A/mute swan/Czech Republic/964-17/2017 (H5N8) | 2017-01-19 | State Veterinary Institute Prague | State Veterinary Institute Prague | Nagy,A |
| EPI_ISL_268935 | A/mute swan/Czech Republic/572-17_3/2017 (H5N8) | 2017-01-13 | State Veterinary Institute Prague | State Veterinary Institute Prague | Nagy,A |
| EPI_ISL_266538 | A/Cygnus olor/Belgium/1567/2017 | 2017-02-21 | Not listed | Other Database Import | Steensels,M;Lambrecht,B;Vandenbussche,F;VanBorm,S |
| EPI_ISL_262057 | A/mute swan/Germany-NI/AR1529-L02145/2017 | 2017-02-14 | Not listed | Friedrich-Loeffler-Institut | Pohlmann,A |
| EPI_ISL_260724 | A/mute swan/Ibaraki/1/2016 | 2016-12-13 | Not listed | Hokkaido University | MasatoshiOkamatsu |
| EPI_ISL_258661 | A/mute swan/Germany-TH/R1126/2017 | 2017-02-04 | Not listed | Friedrich-Loeffler-Institut | Starick,E |
| EPI_ISL_257700 | A/mute swan/Czech Republic/1813-17/2017 (H5N8) | 2017-02-05 | Not listed | State Veterinary Institute Prague | Nagy,A |
| EPI_ISL_256523 | A/mute swan/Ibaraki/7/2016 | 2016-12-14 | Not listed | Hokkaido University | MasatoshiOkamatsu |
| EPI_ISL_256304 | A/mute swan/Czech Republic/581-17/2017 (H5N8) | 2017-01-13 | Not listed | State Veterinary Institute Prague | Nagy,A |
| EPI_ISL_256302 | A/mute swan/Czech Republic/499-17/2017 (H5N8) | 2017-01-11 | Not listed | State Veterinary Institute Prague | Nagy,A |
| EPI_ISL_255917 | A/mute swan/Poland/64/2017 | 2017-01-31 | Not listed | National Veterinary Research Institut Poland, PIWet-PIB | Swieton,E;Smietanka,K |
| EPI_ISL_255203 | A/Mute swan/Hungary/3513/2017 | 2017-01-27 | Central Agricultural Office Veterinary Diagnostic Directorate | Central Agricultural Office Veterinary Diagnostic Directorate | Dan,A |
| EPI_ISL_250902 | A/mute swan/Czech Republic/879-17/2017 (H5N8) | 2017-01-18 | Not listed | State Veterinary Institute Prague | Nagy,Alexander |
| EPI_ISL_248934 | A/mute swan/Czech Republic/722-17_1/2017 (H5N8) | 2017-01-14 | State Veterinary Institute Prague | State Veterinary Institute Prague | Nagy,A |
| EPI_ISL_248657 | A/mute swan/Czech Republic/653-17/2017 (H5N8) | 2017-01-15 | State Veterinary Institute Prague | State Veterinary Institute Prague | Nagy,Alexander |
| EPI_ISL_247723 | A/mute swan/Krasnodar/25/2017 | 2017-01-06 | Not listed | State Research Center of Virology and Biotechnology (VECTOR) | Susloparov,I;Goncharova,N;Kolosova,N;Marchenko,V;Ryzhikov,A |
| EPI_ISL_243693 | A/mute swan/Croatia/30/2017 | 2017-01-22 | Not listed | Croatian Veterinary Institute | Savić,Vladimir |
| EPI_ISL_240671 | A/mute swan/Croatia/104/2016 | 2016-12-29 | Not listed | Croatian Veterinary Institute | Savić,Vladimir |
| EPI_ISL_240670 | A/mute swan/Croatia/9/2017 | 2017-01-03 | Not listed | Croatian Veterinary Institute | Savić,Vladimir |
| EPI_ISL_240106 | A/mute swan/Poland/108/2016 | 2016-12-21 | Not listed | National Veterinary Research Institut Poland, PIWet-PIB | Swieton,E;Smietanka,K |
| EPI_ISL_240101 | A/mute swan/Croatia/102/2016 | 2016-12-27 | Not listed | Croatian Veterinary Institute | Savić,Vladimir |
| EPI_ISL_238197 | A/mute swan/Croatia/78/2016 | 2016-11-12 | Not listed | Croatian Veterinary Institute | Savić,Vladimir |
| EPI_ISL_238196 | A/mute swan/Croatia/70/2016 | 2016-10-30 | Not listed | Croatian Veterinary Institute | Savić,Vladimir |
| EPI_ISL_300981 | A/turkey/Poland/15/2017 | 2017-01-05 | Not listed | National Veterinary Research Institut Poland, PIWet-PIB | Swieton,E;Smietanka,K |
| EPI_ISL_300980 | A/turkey/Poland/005/2017 | 2017-01-02 | Not listed | National Veterinary Research Institut Poland, PIWet-PIB | Swieton,E;Smietanka,K |
| EPI_ISL_300979 | A/turkey/Poland/004/2017 | 2017-01-02 | Not listed | National Veterinary Research Institut Poland, PIWet-PIB | Swieton,E;Smietanka,K |
| EPI_ISL_300898 | A/turkey/Poland/107/2016 | 2016-12-27 | Not listed | National Veterinary Research Institut Poland, PIWet-PIB | Swieton,E;Smietanka,K |
| EPI_ISL_300841 | A/turkey/Poland/100/2016 | 2016-12-26 | Not listed | National Veterinary Research Institut Poland, PIWet-PIB | Swieton,E;Smietanka,K |
| EPI_ISL_300808 | A/turkey/Poland/93/2016 | 2016-12-25 | Not listed | National Veterinary Research Institut Poland, PIWet-PIB | Swieton,E;Smietanka,K |
| EPI_ISL_300705 | A/turkey/Poland/285/2017 | 2017-03-08 | National Veterinary Research Institut Poland, PIWet-PIB | National Veterinary Research Institut Poland, PIWet-PIB | Swieton,E;Smietanka,K |
| EPI_ISL_300704 | A/turkey/Poland/192/2017 | 2017-02-20 | National Veterinary Research Institut Poland, PIWet-PIB | National Veterinary Research Institut Poland, PIWet-PIB | Swieton,E;Smietanka,K |
| EPI_ISL_300701 | A/turkey/Poland/94/2017 | 2017-02-03 | National Veterinary Research Institut Poland, PIWet-PIB | National Veterinary Research Institut Poland, PIWet-PIB | Swieton,E;Smietanka,K |
| EPI_ISL_300700 | A/turkey/Poland/72/2017 | 2017-01-27 | National Veterinary Research Institut Poland, PIWet-PIB | National Veterinary Research Institut Poland, PIWet-PIB | Swieton,E;Smietanka,K |
| EPI_ISL_300686 | A/turkey/Poland/63/2016 | 2016-12-12 | National Veterinary Research Institut Poland, PIWet-PIB | National Veterinary Research Institut Poland, PIWet-PIB | Swieton,E;Smietanka,K |
| EPI_ISL_298650 | A/turkey/Israel/1076/2016 | 2016-12-24 | Not listed | Other Database Import | Shkoda,I;Lapin,K;Simanov,L;Lublin,A |
| EPI_ISL_298640 | A/turkey/Israel/1045/2016 | 2016-12-20 | Not listed | Other Database Import | Shkoda,I;Lapin,K;Simanov,L;Lublin,A |
| EPI_ISL_282133 | A/turkey/Czech Republic/38-17_1/2017 (H5N8) | 2017-01-03 | State Veterinary Institute Prague | State Veterinary Institute Prague | Nagy,A |
| EPI_ISL_256817 | A/turkey/Germany-NI/R9807/2016 | 2016-11-22 | Not listed | Friedrich-Loeffler-Institut | Starick,E |
| EPI_ISL_256460 | A/Turkey/Hungary/53136/2016 | 2016-11-01 | National Food Chain Safety Office Veterinary Diagnostic Directorate Laboratory for Molecular Biology | Central Agricultural Office Veterinary Diagnostic Directorate | Dan,A |
| EPI_ISL_256306 | A/turkey/Italy/17VIR1574-1/2017 | 2017-02-21 | Istituto Zooprofilattico Sperimentale Delle Venezie | Istituto Zooprofilattico Sperimentale Delle Venezie | Zecchin,B;Fusaro,A;Milani,A;Schivo,A;Salviato,A;Zamperin,G;Marciano,S;Ormelli,S;Terregino,C;Monne,I |
| EPI_ISL_255187 | A/turkey/Italy/17VIR1452-22/2017 | 2017-02-16 | Istituto Zooprofilattico Sperimentale Delle Venezie | Istituto Zooprofilattico Sperimentale Delle Venezie | Zecchin,B;Fusaro,A;Milani,A;Schivo,A;Salviato,A;Zamperin,G;Marciano,S;Ormelli,S;Terregino,C;Monne,I |
| EPI_ISL_255186 | A/turkey/Italy/17VIR1338-3/2017 | 2017-02-14 | Istituto Zooprofilattico Sperimentale Delle Venezie | Istituto Zooprofilattico Sperimentale Delle Venezie | Zecchin,B;Fusaro,A;Milani,A;Schivo,A;Salviato,A;Zamperin,G;Marciano,S;Ormelli,S;Terregino,C;Monne,I |
| EPI_ISL_255182 | A/turkey/Italy/17VIR538-1/2017 | 2017-01-20 | Istituto Zooprofilattico Sperimentale Delle Venezie | Istituto Zooprofilattico Sperimentale Delle Venezie | Zecchin,B;Fusaro,A;Milani,A;Schivo,A;Salviato,A;Zamperin,G;Marciano,S;Ormelli,S;Terregino,C;Monne,I |
| EPI_ISL_244138 | A/turkey/Missouri/16-014037-7/2016 | 2016-04-29 | Not listed | Other Database Import | Killian,ML |
| EPI_ISL_243049 | A/turkey/Germany-SH/R425/2017 | 2017-01-22 | Not listed | Friedrich-Loeffler-Institut | Starick,E |
| EPI_ISL_243048 | A/turkey/Czech Republic/38-17_5/2017 (H5N8) | 2017-01-03 | Not listed | State Veterinary Institute Prague | Nagy,Alexander |
| EPI_ISL_240892 | A/turkey/Germany-NI/R10523/2016 | 2016-12-13 | Not listed | Friedrich-Loeffler-Institut | Starick,E |
| EPI_ISL_240108 | A/turkey/Poland/83/2016 | 2016-12-19 | Not listed | National Veterinary Research Institut Poland, PIWet-PIB | Swieton,E;Smietanka,K |
| EPI_ISL_240107 | A/turkey/Poland/78/2016 | 2016-12-16 | Not listed | National Veterinary Research Institut Poland, PIWet-PIB | Swieton,E;Smietanka,K |
| EPI_ISL_238037 | A/turkey/Germany-SH/R8595/2016 | 2016-11-09 | Not listed | Friedrich-Loeffler-Institut | Starick,E |
| EPI_ISL_278031 | A/Quail/Zhanjiang/16887/2016 | 2016-08-15 | South China Agricultural University | South China Agricultural University | Weixin,J |
| EPI_ISL_308779 | A/great cormorant/Croatia/18/2017 | 2017-01-11 | Not listed | Croatian Veterinary Institute | Savić,Vladimir |
| EPI_ISL_308778 | A/great cormorant/Croatia/16/2017 | 2017-01-11 | Not listed | Croatian Veterinary Institute | Savić,Vladimir |
| EPI_ISL_298651 | A/great egret/Israel/1084/2016 | 2016-12-25 | Not listed | Other Database Import | Shkoda,I;Lapin,K;Simanov,L;Lublin,A |
| EPI_ISL_298647 | A/cormorant/Israel/1035/2016 | 2016-12-19 | Not listed | Other Database Import | Shkoda,I;Lapin,K;Simanov,L;Lublin,A |
| EPI_ISL_297140 | A/pigeon/Egypt/HASHM4/2016 | 2016-01 | Not listed | Other Database Import | Ahmed,HA;Tolba,HMN;AbouElez,RMM;Elsohaby,I |
| EPI_ISL_297139 | A/pigeon/Egypt/HASHM3/2016 | 2016-01 | Not listed | Other Database Import | Ahmed,HA;Tolba,HMN;AbouElez,RMM;Elsohaby,I |
| EPI_ISL_295754 | A/cackling goose/Aichi/2312T020/2016 | 2016-12-17 | Not listed | Other Database Import | Soda,K;Usui,T;Ito,H;Ozaki,H;Yamaguchi,T;Ito,T |
| EPI_ISL_293505 | A/coot/Shiga/2501T010/2017 | 2017-01-04 | Not listed | Other Database Import | Soda,K;Usui,T;Ito,H;Ozaki,H;Yamaguchi,T;Ito,T |
| EPI_ISL_287800 | A/spoonbill/Taiwan/DB645/2017 | 2017-12-01 | Not listed | Animal Health Research Institute | Yu-Ju,Lin;Li-Hsuan,Chen;Wan-Chen,Li;Yen-Ping,Chen;Yu-Pin,Liu;Fan,Lee;Wen-Jane,Tu |
| EPI_ISL_279040 | A/crane/Kagoshima/KU-53/2016 | 2016-12-14 | Not listed | Kagoshima University | MakatoOzawa |
| EPI_ISL_279038 | A/crane/Kagoshima/KU-48/2016 | 2016-12-06 | Not listed | Kagoshima University | MakatoOzawa |
| EPI_ISL_279036 | A/crane/Kagoshima/KU-45/2016 | 2016-12-05 | Not listed | Kagoshima University | MakatoOzawa |
| EPI_ISL_279035 | A/crane/Kagoshima/KU-44/2016 | 2016-12-03 | Not listed | Kagoshima University | MakatoOzawa |
| EPI_ISL_279034 | A/crane/Kagoshima/KU-43/2016 | 2016-12-03 | Not listed | Kagoshima University | MakatoOzawa |
| EPI_ISL_279032 | A/crane/Kagoshima/KU-33/2016 | 2016-11-26 | Not listed | Kagoshima University | MakatoOzawa |
| EPI_ISL_279031 | A/crane/Kagoshima/KU-34/2016 | 2016-11-25 | Not listed | Kagoshima University | MakatoOzawa |
| EPI_ISL_279030 | A/crane/Kagoshima/KU-31/2016 | 2016-11-25 | Not listed | Kagoshima University | MakatoOzawa |
| EPI_ISL_279027 | A/crane/Kagoshima/KU-27/2016 | 2016-11-23 | Not listed | Kagoshima University | MakatoOzawa |
| EPI_ISL_279025 | A/crane/Kagoshima/KU-25/2016 | 2016-11-23 | Not listed | Kagoshima University | MakatoOzawa |
| EPI_ISL_279022 | A/crane/Kagoshima/KU-14/2016 | 2016-11-23 | Not listed | Kagoshima University | MakatoOzawa |
| EPI_ISL_279017 | A/crane/Kagoshima/KU-10/2016 | 2016-11-21 | Not listed | Kagoshima University | MakatoOzawa |
| EPI_ISL_279012 | A/crane/Kagoshima/KU-9/2016 | 2016-11-20 | Not listed | Kagoshima University | MakatoOzawa |
| EPI_ISL_279011 | A/crane/Kagoshima/KU-8/2016 | 2016-11-20 | Not listed | Kagoshima University | MakatoOzawa |
| EPI_ISL_279010 | A/crane/Kagoshima/KU-6/2016 | 2016-11-19 | Not listed | Kagoshima University | MakatoOzawa |
| EPI_ISL_269600 | A/Great_Crested_Grebe/Netherlands/2/2016 | 2016-12-21 | Erasmus Medical Center | Erasmus Medical Center | Poen,MJ;VanDerJeugd,HP;Vuong,O;Scheuer,RD;Kleyheeg,E;Bestebroer,TM;Begeman,L;vandenBrand,JMA;Kuiken,T;Fouchier,RAM |
| EPI_ISL_268964 | A/grey heron/Czech Republic/1680-17_2/2017 (H5N8) | 2017-02-02 | State Veterinary Institute Prague | State Veterinary Institute Prague | Nagy,A |
| EPI_ISL_268654 | A/G_c_grebe/NL-Monnickendam/16013865-009-010/2016 | 2016-11-08 | Wageningen Bioveterinary Research | Wageningen Bioveterinary Research | Beerens,N;Heutink,R;Harders,F;Verschuren-Pritz,S;Bossers,A;Koch,G;Bergervoet,S |
| EPI_ISL_268628 | A/Crow/NL-Oostwoud/16015372-004/2016 | 2016-12-05 | Wageningen Bioveterinary Research | Wageningen Bioveterinary Research | Beerens,N;Heutink,R;Harders,F;Verschuren-Pritz,S;Bossers,A;Koch,G;Bergervoet,S |
| EPI_ISL_256512 | A/coot/Iwate/13/2016 | 2016-12-22 | Not listed | Hokkaido University | MasatoshiOkamatsu |
| EPI_ISL_256495 | A/owl/Hokkaido/X6/2016 | 2016-12-12 | Graduate School of Veterinary Medicine, Hokkaido University | Hokkaido University | MasatoshiOkamatsu |
| EPI_ISL_256305 | A/shelduck/Italy/17VIR1572-24/2017 | 2017-01-03 | Istituto Zooprofilattico Sperimentale Delle Venezie | Istituto Zooprofilattico Sperimentale Delle Venezie | Zecchin,B;Fusaro,A;Milani,A;Schivo,A;Salviato,A;Zamperin,G;Marciano,S;Ormelli,S;Terregino,C;Monne,I |
| EPI_ISL_255189 | A/gadwall/Italy/17VIR133-2/2017 | 2017-01-10 | Istituto Zooprofilattico Sperimentale Delle Venezie | Istituto Zooprofilattico Sperimentale Delle Venezie | Zecchin,B;Fusaro,A;Milani,A;Schivo,A;Salviato,A;Zamperin,G;Marciano,S;Ormelli,S;Terregino,C;Monne,I |
| EPI_ISL_247715 | A/long-eared owl/Voronezh/15/2017 | 2017-01-06 | Not listed | State Research Center of Virology and Biotechnology (VECTOR) | Susloparov,I;Goncharova,N;Kolosova,N;Marchenko,V;Ryzhikov,A |
| EPI_ISL_237554 | A/painted stork/India/10CA03/2016 | 2016-10-20 | ICAR-National Institute of High Security Animal Diseases | ICAR-National Institute of High Security Animal Diseases | Nagarajan,Shanmugasundaram;Shukla,Shweta;Kumar,Manoj;Murugkar,HV;Tosh,Chakradhar;Singh,VijendraPal |
| EPI_ISL_230820 | A/great crested grebe/Tyva/34/2016 | 2016-05-25 | State Research Center of Virology and Biotechnology (VECTOR) | WHO National Influenza Centre Russian Federation | Fadeev,A;Komissarov,A;Egorova,A;Sintsova,K;Musaeva,T;Susloparov,I;Marchenko,V;Ryzhikov,A |
| EPI_ISL_205825 | A/great_egret/Hong_Kong/00032/2016 | 2016-01-02 | Agriculture, Fisheries and Conservation Department | Agriculture, Fisheries and Conservation Department | LUK,GeraldineSM;LEE,FrancesE;IP,Sin-Ming;WAI,ChrisKL |
| EPI_ISL_278205 | A/cat/Zhejiang/C2/2016 | 2016-01-12 | Not listed | Other Database Import | Cao,X;Yang,F;Wu,H;Xu,L |
| EPI_ISL_278204 | A/cat/Zhejiang/C1/2016 | 2016-01-12 | Not listed | Other Database Import | Cao,X;Yang,F;Wu,H;Xu,L |
| EPI_ISL_262935 | A/swine/Nigeria/75/2016 | 2016-01-30 | National Veterinary Research Institute | Friedrich-Loeffler-Institut | Starick,E |
| EPI_ISL_262934 | A/swine/Nigeria/73/2016 | 2016-01-30 | National Veterinary Research Institute | Friedrich-Loeffler-Institut | Meseko,Clement;Starick,Elke |
| EPI_ISL_262933 | A/swine/Nigeria/49/2016 | 2016-01-09 | National Veterinary Research Institute | Friedrich-Loeffler-Institut | Meseko,Clement;Starick,Elke |
| EPI_ISL_309423 | A/environment/South Carolina/UGAI17-2513/2017 | 2017-05-12 | Not listed | Other Database Import | Tan,G;Pickett,B;Fedorova,N;Amedeo,P;Isom,R;Hu,L;Christensen,J;Durbin,A;Williams,T;Arumemi,F;Poulson,R;Bao,Y;Sanders,R;Zhdanov,S;Kiryutin,B;Lipman,DJ;Tatusova,T;Hatcher,E;Stallknecht,D |
| EPI_ISL_309421 | A/environment/South Carolina/UGAI17-2485/2017 | 2017-05-11 | Not listed | Other Database Import | Tan,G;Pickett,B;Fedorova,N;Amedeo,P;Isom,R;Hu,L;Christensen,J;Durbin,A;Williams,T;Arumemi,F;Poulson,R;Bao,Y;Sanders,R;Zhdanov,S;Kiryutin,B;Lipman,DJ;Tatusova,T;Hatcher,E;Stallknecht,D |
| EPI_ISL_309420 | A/environment/South Carolina/UGAI17-2484/2017 | 2017-05-11 | Not listed | Other Database Import | Tan,G;Pickett,B;Fedorova,N;Amedeo,P;Isom,R;Hu,L;Christensen,J;Durbin,A;Williams,T;Arumemi,F;Poulson,R;Bao,Y;Sanders,R;Zhdanov,S;Kiryutin,B;Lipman,DJ;Tatusova,T;Hatcher,E;Stallknecht,D |
| EPI_ISL_307699 | A/environment/South Carolina/UGAI17-2360/2017 | 2017-05-10 | Not listed | Other Database Import | Tan,G;Pickett,B;Fedorova,N;Amedeo,P;Isom,R;Hu,L;Christensen,J;Durbin,A;Williams,T;Arumemi,F;Poulson,R;Bao,Y;Sanders,R;Zhdanov,S;Kiryutin,B;Lipman,DJ;Tatusova,T;Hatcher,E;Stallknecht,D |
| EPI_ISL_307698 | A/environment/South Carolina/UGAI17-2441/2017 | 2017-05-11 | Not listed | Other Database Import | Tan,G;Pickett,B;Fedorova,N;Amedeo,P;Isom,R;Hu,L;Christensen,J;Durbin,A;Williams,T;Arumemi,F;Poulson,R;Bao,Y;Sanders,R;Zhdanov,S;Kiryutin,B;Lipman,DJ;Tatusova,T;Hatcher,E;Stallknecht,D |
| EPI_ISL_307695 | A/environment/South Carolina/UGAI17-2243/2017 | 2017-05-10 | Not listed | Other Database Import | Tan,G;Pickett,B;Fedorova,N;Amedeo,P;Isom,R;Hu,L;Christensen,J;Durbin,A;Williams,T;Arumemi,F;Poulson,R;Bao,Y;Sanders,R;Zhdanov,S;Kiryutin,B;Lipman,DJ;Tatusova,T;Hatcher,E;Stallknecht,D |
| EPI_ISL_307692 | A/environment/South Carolina/UGAI17-2246/2017 | 2017-05-10 | Not listed | Other Database Import | Tan,G;Pickett,B;Fedorova,N;Amedeo,P;Isom,R;Hu,L;Christensen,J;Durbin,A;Williams,T;Arumemi,F;Poulson,R;Bao,Y;Sanders,R;Zhdanov,S;Kiryutin,B;Lipman,DJ;Tatusova,T;Hatcher,E;Stallknecht,D |
| EPI_ISL_307685 | A/environment/South Carolina/UGAI17-2223/2017 | 2017-05-10 | Not listed | Other Database Import | Tan,G;Pickett,B;Fedorova,N;Amedeo,P;Isom,R;Hu,L;Christensen,J;Durbin,A;Williams,T;Arumemi,F;Poulson,R;Bao,Y;Sanders,R;Zhdanov,S;Kiryutin,B;Lipman,DJ;Tatusova,T;Hatcher,E;Stallknecht,D |
| EPI_ISL_307672 | A/environment/South Carolina/UGAI17-2213/2017 | 2017-05-10 | Not listed | Other Database Import | Tan,G;Pickett,B;Fedorova,N;Amedeo,P;Isom,R;Hu,L;Christensen,J;Durbin,A;Williams,T;Arumemi,F;Poulson,R;Bao,Y;Sanders,R;Zhdanov,S;Kiryutin,B;Lipman,DJ;Tatusova,T;Hatcher,E;Stallknecht,D |
| EPI_ISL_307671 | A/environment/New Jersey/UGAI17-2575/2017 | 2017-05-18 | Not listed | Other Database Import | Tan,G;Pickett,B;Fedorova,N;Amedeo,P;Isom,R;Hu,L;Christensen,J;Durbin,A;Williams,T;Arumemi,F;Poulson,R;Bao,Y;Sanders,R;Zhdanov,S;Kiryutin,B;Lipman,DJ;Tatusova,T;Hatcher,E;Stallknecht,D |
| EPI_ISL_307669 | A/environment/New Jersey/UGAI17-2302/2017 | 2017-05-16 | Not listed | Other Database Import | Tan,G;Pickett,B;Fedorova,N;Amedeo,P;Isom,R;Hu,L;Christensen,J;Durbin,A;Williams,T;Arumemi,F;Poulson,R;Bao,Y;Sanders,R;Zhdanov,S;Kiryutin,B;Lipman,DJ;Tatusova,T;Hatcher,E;Stallknecht,D |
| EPI_ISL_307667 | A/environment/Georgia/UGAI17-2201/2017 | 2017-05-08 | Not listed | Other Database Import | Tan,G;Pickett,B;Fedorova,N;Amedeo,P;Isom,R;Hu,L;Christensen,J;Durbin,A;Williams,T;Arumemi,F;Poulson,R;Bao,Y;Sanders,R;Zhdanov,S;Kiryutin,B;Lipman,DJ;Tatusova,T;Hatcher,E;Stallknecht,D |
| EPI_ISL_307642 | A/environment/South Carolina/UGAI17-2332/2017 | 2017-05-10 | Not listed | Other Database Import | Tan,G;Pickett,B;Fedorova,N;Amedeo,P;Isom,R;Hu,L;Christensen,J;Durbin,A;Williams,T;Arumemi,F;Poulson,R;Bao,Y;Sanders,R;Zhdanov,S;Kiryutin,B;Lipman,DJ;Tatusova,T;Hatcher,E;Stallknecht,D |
| EPI_ISL_293486 | A/environment/Niigata/5/2016 | 2016-11-28 | Not listed | Other Database Import | Soda,K;Usui,T;Ito,H;Ozaki,H;Yamaguchi,T;Ito,T |
| EPI_ISL_293485 | A/environment/Aomori/R1/2016 | 2016-11-28 | Not listed | Other Database Import | Soda,K;Usui,T;Ito,H;Ozaki,H;Yamaguchi,T;Ito,T |
| EPI_ISL_293484 | A/environment/Aomori/4/2016 | 2016-11-28 | Not listed | Other Database Import | Soda,K;Usui,T;Ito,H;Ozaki,H;Yamaguchi,T;Ito,T |
| EPI_ISL_266514 | A/environment/Zhongshan/ZS01/2016 | 2016-02-29 | Not listed | Other Database Import | Wu,Y;Lin,J;Shi,W;Xie,Y |
| EPI_ISL_244521 | A/environment/Korea/W543/2016 | 2016-11-18 | Not listed | Other Database Import | Si,YJ;Lee,IW;Kim,EH;Kim,YI;Kwon,HI;Park,SJ;Nguyen,HD;Kim,SM;Kwon,JJ;Choi,WS;Beak,YH;Song,MS;Kim,CJ;Webby,RJ;Choi,YK;Si,Y-J;Kwon,H-I;Lee,I-W;Dihn,HN;Kim,Y-I;Choi,Y-K |
| EPI_ISL_244519 | A/environment/Korea/W541/2016 | 2016-11-18 | Not listed | Other Database Import | Si,YJ;Lee,IW;Kim,EH;Kim,YI;Kwon,HI;Park,SJ;Nguyen,HD;Kim,SM;Kwon,JJ;Choi,WS;Beak,YH;Song,MS;Kim,CJ;Webby,RJ;Choi,YK;Si,Y-J;Kwon,H-I;Lee,I-W;Dihn,HN;Kim,Y-I;Choi,Y-K |
| EPI_ISL_266819 | A/enviroment/Guangdong/F4/2016 | 2016-03-02 | Not listed | South China Agricultural University | Qin,F |
| EPI_ISL_238605 | A/environment/Kagoshima/KU-ngr-I/2016(H5N6) | 2016-11-14 | Kagoshima University | Kagoshima University | MakatoOzawa |
| EPI_ISL_221711 | A/duck/Guangdong/01.01 SZSGXJK003-W/2016 | 2016-01-01 | Institute of Microbiology, Chinese Academy of Sciences | Institute of Microbiology, Chinese Academy of Sciences | Bi,Y |
| EPI_ISL_239267 | A/mandarin duck/Korea/T102-1/2016(H5N6) | 2016-11-13 | Not listed | Animal and Plant Quarantine Agency (S-2026) | Lee,YJ |
| EPI_ISL_243353 | A/duck/Pennsylvania/10218-MA/1984 | 2016 | Not listed | Other Database Import | Esmagambetov,IB;Tutykhina,IL;Bagaev,AV;Pichugin,AV;Shcherbinin,DN;Sedova,ES;Lysenko,AA;Logunov,DY;Shmarov,MM;Naroditsky,BS;Ataullakhanov,RI;Gintsburg,AL;Voronina,OL;Aksenova,EI;Kunda,MS;Ryzhova,NN;Semenov,AN;Botikov,AG |
| EPI_ISL_234239 | A/reassortant/JLUMV_RG1(chicken/Egypt/VSVRI/2009 x Puerto Rico/8/1934) | 2016 | Not listed | Other Database Import | Mostafa,A;Ibrahim,SM;Pleschka,S |
| EPI_ISL_305786 | A/humman/China/MDCK/2017 | 2017-12-07 | Not listed | Other Database Import | Duo,L |
| EPI_ISL_275433 | A/unknown/Tatarstan/94/2017 | 2017-05-08 | Not listed | State Research Center of Virology and Biotechnology (VECTOR) | Susloparov,I;Goncharova,N;Kolosova,N;Marchenko,V;Ryzhikov,A |
| EPI_ISL_275432 | A/unknown/Tatarstan/86/2017 | 2017-05-08 | Not listed | State Research Center of Virology and Biotechnology (VECTOR) | Susloparov,I;Goncharova,N;Kolosova,N;Marchenko,V;Ryzhikov,A |
| EPI_ISL_305763 | A/China/MDCK/2017 | 2017-12-07 | Not listed | Other Database Import | Zhou,J |
| EPI_ISL_304404 | A/Fujian-Sanyuan/21099/2017 | 2017-12-25 | Fujian Provincial Center for Disease Control and Prevention | WHO Chinese National Influenza Center | Yang,L |
| EPI_ISL_284651 | A/Anhui/33163/2016 | 2016-04-29 | Anhui Provincial Center for Disease Control and Prevention | WHO Chinese National Influenza Center | Zhang,Ye;Li,Xiyan;He,Jun;Wang,Dayan |
| EPI_ISL_281682 | A/Indonesia/NIHRD17109/2017 | 2017-09-08 | National Institute of Health Research and Development | National Institute of Health Research and Development | Pawestri,HA;Nugraha,AA;Pratiwi,E;Setiawaty,V |
| EPI_ISL_256213 | A/Hubei/29578/2016 | 2016-04-15 | Not listed | WHO Chinese National Influenza Center | Yang,L |
| EPI_ISL_240704 | A/Guangxi/55726/2016 | 2016-11-24 | Not listed | WHO Chinese National Influenza Center | Wang,D;Zhao,X;Li,Z;Li,X;Chen,W;Yang,L;Shu,Y |
| EPI_ISL_240703 | A/Hunan/55555/2016 | 2016-11-18 | Not listed | WHO Chinese National Influenza Center | Wang,D;Bo,H;Gao,R;Li,X;Chen,W;Yang,L;Shu,Y |
| EPI_ISL_207051 | A/Shenzhen/TH002/2016(H5N6) | 2016-01-06 | CAS Key Laboratory of Pathogenic Microbiology and Immunology, Institute of Microbiology, Chinese Academy of Sciences | Institute of Microbiology, Chinese Academy of Sciences | Bi,Y |
| EPI_ISL_206036 | A/Shenzhen/1/2016 | 2016-01-07 | Not listed | WHO Chinese National Influenza Center | Fang,S;Yang,L |
| EPI_ISL_308809 | A/pigeon/Cameroon/17RS1661-4/2017 | 2017-01 | Not listed | Other Database Import | Wade,A;Zecchin,B;Jumbo,SD;Fusaro,A;Taiga,T;Bianco,A;PouemeN,R;Salomoni,A;FeussomKameni,JM;Zamperin,G;Kazi,JP;Nenkam,R;Foupouapouognigni,Y;Abdoulkadiri,S;Yaya,A;Monne,I |
| EPI_ISL_308808 | A/Indian peafowl/Cameroon/17RS1661-6/2017 | 2017-01 | Not listed | Other Database Import | Wade,A;Zecchin,B;Jumbo,SD;Fusaro,A;Taiga,T;Bianco,A;PouemeN,R;Salomoni,A;FeussomKameni,JM;Zamperin,G;Kazi,JP;Nenkam,R;Foupouapouognigni,Y;Abdoulkadiri,S;Yaya,A;Monne,I |
| EPI_ISL_283705 | A/Chicken/Yunnan/YN-7/2016 (H5N6) | 2016-07-04 | South China Agricultural University | South China Agricultural University | Jiahao,Z;Guangjie,L;Ronghua,Z;Hexing,W;Guanming,S;Bo,L;Wenbao,Q;Ming,L |
| EPI_ISL_279790 | A/wildfowl/Shandong/SD04/2016 | 2016-01 | Not listed | Other Database Import | Meng,K;Wang,Y;Yuan,X;Zhang,Y;Qi,L |
| EPI_ISL_272859 | A/grey heron/W779/2017 | 2017-01-27 | Not listed | Other Database Import | Jeong,J;Woo,C;Kim,Y;Lee,K;Jo,SD;Son,KD;Oem,JK;Wang,SJ;Shin,J;Jheong,W |
| EPI_ISL_268981 | A/spot-billed pelican/Czech Republic/2270-17/2017 (H5N5) | 2017-02-14 | State Veterinary Institute Prague | State Veterinary Institute Prague | Nagy,A |
| EPI_ISL_266943 | A/snowy owl/Akita/0051D010-4/2016 | 2016-11-23 | Not listed | Other Database Import | Soda,K;Usui,T;Ito,H;Ozaki,H;Yamaguchi,T;Ito,T |
| EPI_ISL_266942 | A/snowy owl/Akita/0051D008/2016 | 2016-11-23 | Not listed | Other Database Import | Soda,K;Usui,T;Ito,H;Ozaki,H;Yamaguchi,T;Ito,T |
| EPI_ISL_266941 | A/snowy owl/Akita/0051D007/2016 | 2016-11-23 | Not listed | Other Database Import | Soda,K;Usui,T;Ito,H;Ozaki,H;Yamaguchi,T;Ito,T |
| EPI_ISL_260059 | A/cormorant/Germany-SH/R896/2017 | 2017-01-30 | Not listed | Friedrich-Loeffler-Institut | Starick,E |
| EPI_ISL_259525 | A/egret/Germany-SH/R1459/2017 | 2017-02-14 | Not listed | Friedrich-Loeffler-Institut | Starick,E |
| EPI_ISL_258527 | A/tawny owl/Germany-SN/R1186/2017 | 2017-02-08 | Not listed | Friedrich-Loeffler-Institut | Starick,E |
| EPI_ISL_258526 | A/white stork/Germany-TH/R1149/2017 | 2017-02-06 | Not listed | Friedrich-Loeffler-Institut | Starick,E |
| EPI_ISL_256453 | A/Duck/Hungary/54738/2016 | 2016-11-09 | National Food Chain Safety Office Veterinary Diagnostic Directorate Laboratory for Molecular Biology | Central Agricultural Office Veterinary Diagnostic Directorate | Dan,A |
| EPI_ISL_255937 | A/Pheasant/Hungary/7685/2017 | 2017-02-24 | National Food Chain Safety Office Veterinary Diagnostic Directorate Laboratory for Molecular Biology | Central Agricultural Office Veterinary Diagnostic Directorate | Dan,A |
| EPI_ISL_255936 | A/Pheasant/Hungary/6553/2017 | 2017-02-16 | National Food Chain Safety Office Veterinary Diagnostic Directorate Laboratory for Molecular Biology | Central Agricultural Office Veterinary Diagnostic Directorate | Dan,A |
| EPI_ISL_255935 | A/Mute swan/Hungary/6276/2017 | 2017-02-16 | National Food Chain Safety Office Veterinary Diagnostic Directorate Laboratory for Molecular Biology | Central Agricultural Office Veterinary Diagnostic Directorate | Dan,A |
| EPI_ISL_255934 | A/Mute swan/Hungary/6092/2017 | 2017-02-15 | National Food Chain Safety Office Veterinary Diagnostic Directorate Laboratory for Molecular Biology | Central Agricultural Office Veterinary Diagnostic Directorate | Dan,A |
| EPI_ISL_255933 | A/Cormorant/Hungary/6102/2017 | 2017-02-15 | National Food Chain Safety Office Veterinary Diagnostic Directorate Laboratory for Molecular Biology | Central Agricultural Office Veterinary Diagnostic Directorate | Dan,A |
| EPI_ISL_255215 | A/Mute swan/Hungary/3137/2017 | 2017-01-26 | Central Agricultural Office Veterinary Diagnostic Directorate | Central Agricultural Office Veterinary Diagnostic Directorate | Dan,A |
| EPI_ISL_255213 | A/GuineaFowl/Hungary/596/2017 | 2017-01-06 | Central Agricultural Office Veterinary Diagnostic Directorate | Central Agricultural Office Veterinary Diagnostic Directorate | Dan,A |
| EPI_ISL_255204 | A/Mute swan/Hungary/3542/2017 | 2017-01-29 | Central Agricultural Office Veterinary Diagnostic Directorate | Central Agricultural Office Veterinary Diagnostic Directorate | Dan,A |
| EPI_ISL_255201 | A/Mute swan/Hungary/2825/2017 | 2017-01-24 | Central Agricultural Office Veterinary Diagnostic Directorate | Central Agricultural Office Veterinary Diagnostic Directorate | Dan,A |
| EPI_ISL_255199 | A/Mute swan/Hungary/2193/2017 | 2017-01-19 | Central Agricultural Office Veterinary Diagnostic Directorate | Central Agricultural Office Veterinary Diagnostic Directorate | Dan,A |
| EPI_ISL_255198 | A/Turkey/Hungary/2030/2017 | 2017-01-18 | Central Agricultural Office Veterinary Diagnostic Directorate | Central Agricultural Office Veterinary Diagnostic Directorate | Dan,A |
| EPI_ISL_255195 | A/Duck/Hungary/1588/2017 | 2017-01-16 | Central Agricultural Office Veterinary Diagnostic Directorate | Central Agricultural Office Veterinary Diagnostic Directorate | Dan,A |
| EPI_ISL_255194 | A/Goose/Hungary/1030/2017 | 2017-01-11 | Central Agricultural Office Veterinary Diagnostic Directorate | Central Agricultural Office Veterinary Diagnostic Directorate | Dan,A |
| EPI_ISL_255174 | A/Goose/Hungary/65817/2016 | 2016-12-18 | Central Agricultural Office Veterinary Diagnostic Directorate | Central Agricultural Office Veterinary Diagnostic Directorate | Dan,A |
| EPI_ISL_243688 | A/black swan/Ibaraki/356T/2017 | 2017-01-21 | Not listed | National Institute of Animal Health | TakehikoSaito |
| EPI_ISL_243686 | A/whooper swan/Ibaraki/351C/2017 | 2017-01-19 | Not listed | National Institute of Animal Health | TakehikoSaito |
| EPI_ISL_243682 | A/black swan/Ibaraki/365T/2017 | 2017-01-24 | Not listed | National Institute of Animal Health | TakehikoSaito |
| EPI_ISL_242415 | A/black swan/Ibaraki/341T/2017 | 2017-01-16 | Not listed | National Institute of Animal Health | Saito,T;Uchida,Y |
| EPI_ISL_242405 | A/whooper swan/Ibaraki/331C/2017 | 2017-01-11 | Not listed | National Institute of Animal Health | Saito,T;Uchida,Y |
| EPI_ISL_241778 | A/muteswan/Ibaraki/277T/2016 | 2016-12-22 | Not listed | National Institute of Animal Health | Saito,T;Takemae,N |
| EPI_ISL_241773 | A/pochard/Ibaraki/268T/2016 | 2016-12-21 | Not listed | National Institute of Animal Health | Saito,T;Takemae,N |
| EPI_ISL_241772 | A/black swan/Ibaraki/310C/2017 | 2017-01-04 | Not listed | National Institute of Animal Health | Saito,T;Takemae,N |
| EPI_ISL_241771 | A/black swan/Ibaraki/271C/2016 | 2016-12-21 | Not listed | National Institute of Animal Health | Saito,T;Takemae,N |
| EPI_ISL_241759 | A/black headed gull/Ibaraki/291T/2016 | 2016-12-26 | Not listed | National Institute of Animal Health | Saito,T;Takemae,N |
| EPI_ISL_241757 | A/black swan/Ibaraki/256C/2016 | 2016-12-19 | Not listed | National Institute of Animal Health | Saito,T;Takemae,N |
| EPI_ISL_241756 | A/mute swan/Ibaraki/252T/2016 | 2016-12-18 | Not listed | National Institute of Animal Health | Saito,T;Takemae,N |
| EPI_ISL_241755 | A/great crested grebe/Ibaraki/287T/2016 | 2016-12-25 | Not listed | National Institute of Animal Health | Saito,T;Takemae,N |
| EPI_ISL_241753 | A/black swan/Ibaraki/290C/2016 | 2016-12-26 | Not listed | National Institute of Animal Health | Saito,T;Takemae,N |
| EPI_ISL_241747 | A/black-headed-gull/Ibaraki/267T/2016 | 2016-12-20 | Not listed | National Institute of Animal Health | Saito,T;Takemae,N |
| EPI_ISL_241746 | A/black headed gull/Ibaraki/282T/2016 | 2016-12-22 | Not listed | National Institute of Animal Health | Saito,T;Takemae,N |
| EPI_ISL_239266 | A/Eurasian eagle owl/Korea/960/2016(H5N6) | 2016-11-05 | Not listed | Animal and Plant Quarantine Agency (S-2026) | Lee,YJ |
| EPI_ISL_234058 | A/common tern /Uvs-Nuur Lake/26/2016 | 2016-05-25 | Research Institute of Experimental and Clinical Medicine | Research Institute of Experimental and Clinical Medicine | Kirill,Sharshov;Olga,Kurskaya;Ivan,Sobolev;Alexander,Alekseev;Tatyana,Alikina;Marsel,Kabilov;Alexander,Shestopalov |
| EPI_ISL_224580 | A/great crested grebe/Uvs-Nuur Lake/341/2016 | 2016-05-25 | Research Institute of Experimental and Clinical Medicine | Research Institute of Experimental and Clinical Medicine | Kirill,Sharshov;Olga,Kurskaya;Ivan,Sobolev;Alexander,Alekseev;Tatyana,Alikina;Marsel,Kabilov;Alexander,Shestopalov |
| EPI_ISL_308806 | A/chicken/Cameroon/17RS1661-1/2017 | 2017-01 | Not listed | Other Database Import | Wade,A;Zecchin,B;Jumbo,SD;Fusaro,A;Taiga,T;Bianco,A;PouemeN,R;Salomoni,A;FeussomKameni,JM;Zamperin,G;Kazi,JP;Nenkam,R;Foupouapouognigni,Y;Abdoulkadiri,S;Yaya,A;Monne,I |
| EPI_ISL_305602 | A/chicken/Cameroon/16VIR3791-22/2016 | 2016-06-01 | Not listed | Other Database Import | Wade,A;Monne,I;Taiga;Fouda,M;Abari,M;Feussom,JM;Zecchin,B;Fusaro,A;Milani,A;Terregino,C |
| EPI_ISL_305600 | A/chicken/Cameroon/16VIR3791-16/2016 | 2016-06-14 | Not listed | Other Database Import | Wade,A;Monne,I;Taiga;Fouda,M;Abari,M;Feussom,JM;Zecchin,B;Fusaro,A;Milani,A;Terregino,C |
| EPI_ISL_305599 | A/chicken/Cameroon/16VIR3791-12/2016 | 2016-05-23 | Not listed | Other Database Import | Wade,A;Monne,I;Taiga;Fouda,M;Abari,M;Feussom,JM;Zecchin,B;Fusaro,A;Milani,A;Terregino,C |
| EPI_ISL_301047 | A/chicken/Poland/199/2017 | 2017-02-21 | Not listed | National Veterinary Research Institut Poland, PIWet-PIB | Swieton,E;Smietanka,K |
| EPI_ISL_301037 | A/chicken/Poland/16/2017 | 2017-01-05 | Not listed | National Veterinary Research Institut Poland, PIWet-PIB | Swieton,E;Smietanka,K |
| EPI_ISL_300978 | A/chicken/Poland/117/2016 | 2016-12-30 | Not listed | National Veterinary Research Institut Poland, PIWet-PIB | Swieton,E;Smietanka,K |
| EPI_ISL_300872 | A/chicken/Poland/103/2016 | 2016-12-27 | Not listed | National Veterinary Research Institut Poland, PIWet-PIB | Swieton,E;Smietanka,K |
| EPI_ISL_300702 | A/chicken/Poland/101/2017 | 2017-02-03 | National Veterinary Research Institut Poland, PIWet-PIB | National Veterinary Research Institut Poland, PIWet-PIB | Swieton,E;Smietanka,K |
| EPI_ISL_300687 | A/chicken/Poland/34/2017 | 2017-01-11 | National Veterinary Research Institut Poland, PIWet-PIB | National Veterinary Research Institut Poland, PIWet-PIB | Swieton,E;Smietanka,K |
| EPI_ISL_298641 | A/chicken/Israel/881/2016 | 2016-12-07 | Not listed | Other Database Import | Shkoda,I;Lapin,K;Simanov,L;Lublin,A |
| EPI_ISL_297932 | A/chicken/Henan/YB0597/2016 | 2016-03 | Not listed | Other Database Import | Liu,K;Gu,M;Gao,R;Li,J;Liu,D;Sun,W;Hu,J;Xu,X;Wang,X;Liu,X |
| EPI_ISL_297930 | A/chicken/Anhui/MZ33/2016 | 2016-02 | Not listed | Other Database Import | Liu,K;Gu,M;Gao,R;Li,J;Liu,D;Sun,W;Hu,J;Xu,X;Wang,X;Liu,X |
| EPI_ISL_293992 | A/chicken/France/160001/2016 | 2016-02-01 | Anses (Ploufragan-Plouzané) | ANSES Agence Nationale De Securite Sanitaire De L’alimentation | Briand,FX |
| EPI_ISL_293987 | A/chicken/France/160102/2016 | 2016-02-03 | Anses (Ploufragan-Plouzané) | ANSES Agence Nationale De Securite Sanitaire De L’alimentation | Briand,FX |
| EPI_ISL_285623 | A/Chicken/South_Africa/S2017/08_0416_38/2017 | 2017-08-23 | Western Cape Provincial Veterinary Laboratory | National Institute of Communicable Diseases | Treurnicht,FK |
| EPI_ISL_285622 | A/duck/Egypt/Buheira-21/2017 | 2017 | Not listed | Friedrich-Loeffler-Institut | Salaheldin,AH;AbdEl-Hamid,HS;Elbestawy,AR;Hafez,MH;Veits,J;Mettenleiter,TC;Abdelwhab,EM' |
| EPI_ISL_285620 | A/chicken/Egypt/Buheira-12/2017 | 2017 | Not listed | Friedrich-Loeffler-Institut | Salaheldin,AH;AbdEl-Hamid,HS;Elbestawy,AR;Hafez,MH;Veits,J;Mettenleiter,TC;Abdelwhab,EM' |
| EPI_ISL_284662 | A/chicken/Vietnam/AI-1702/2017 | 2017-01-21 | Not listed | Other Database Import | Na,W;Kim,H-K;Phan,LV;Yoon,S;Jeong,DG;Song,D |
| EPI_ISL_283703 | A/Chicken/Yunnan/YN-3/2016 (H5N6) | 2016-06-28 | South China Agricultural University | South China Agricultural University | Jiahao,Z;Guangjie,L;Ronghua,Z;Hexing,W;Guanming,S;Bo,L;Wenbao,Q;Ming,L |
| EPI_ISL_282397 | A/chicken/Guangdong/GD1602/2016 | 2016-03-22 | Not listed | Other Database Import | Sun,W |
| EPI_ISL_280368 | A/chicken/Egypt/Qal-3/2016 | 2016-01-15 | Not listed | Other Database Import | Omar,DM;Marden,NA;Gaafar,LMO;El-Ebiary,EA;El-Dougdoug,KA;Othman,BA;Arafa,AA;Hussein,HA |
| EPI_ISL_273846 | A/chicken/Italy/17VIR3078/2017 | 2017-04-07 | Istituto Zooprofilattico Sperimentale Delle Venezie | Istituto Zooprofilattico Sperimentale Delle Venezie | Zecchin,B;Fusaro,A;Zamperin,G;Schivo,A;Salviato,A;Marciano,S;Ormelli,S;Terregino,C;Monne,I |
| EPI_ISL_273844 | A/chicken/Togo/17RS1021-1/2017 | 2017 | Ministere de l'Agriculture, de l'Elevage et de l'Hydraulique | Istituto Zooprofilattico Sperimentale Delle Venezie | Komla,Batawui;Emilie,Go-Maro;Bianca,Zecchin;Alice,Fusaro;Alessia,Schivo;Isabella,Monne |
| EPI_ISL_272875 | A/chicken/Gharbia/5/2016 | 2016-01-25 | Not listed | Other Database Import | Zanaty,A;Saleh,M;ElShehedy,M;ElTarabeley,M;AbdelFatah,S;Selim,A |
| EPI_ISL_272742 | A/chicken/Egypt/Alex-2/2017 | 2017-02-13 | Not listed | Other Database Import | Sedeek,ME-S;Awad,AM;Kandil,NA |
| EPI_ISL_272668 | A/chicken/Mexico/P-14/2016 | 2016-06-02 | Not listed | Other Database Import | Absalon,AE;Lucio-Decanini,E;Ochoa,R |
| EPI_ISL_266812 | A/chicken/Lampung Utara/03160218/2016 | 2016-03-03 | Not listed | Other Database Import | Wibawa,H;Poermadjaja,B;Mulyawan,H;Dharmawan,R;Mahawan,T;Hutagaol,NM;Miswati,Y;Srihanto,EA;Hartawan,DHW;Hendrawati,F;Riyadi,A;Deswarni;TriHarsono,A;Hartaningsih,N;Azhar,M;Stegemen,A;McGrane,J;Rasa,FST |
| EPI_ISL_266811 | A/chicken/Lampung Utara/03160217/2016 | 2016-02-03 | Not listed | Other Database Import | Wibawa,H;Poermadjaja,B;Mulyawan,H;Dharmawan,R;Mahawan,T;Hutagaol,NM;Miswati,Y;Srihanto,EA;Hartawan,DHW;Hendrawati,F;Riyadi,A;Deswarni;TriHarsono,A;Hartaningsih,N;Azhar,M;Stegemen,A;McGrane,J;Rasa,FST |
| EPI_ISL_266805 | A/chicken/Banyuwangi/04160330/2016 | 2016-03-14 | Not listed | Other Database Import | Wibawa,H;Poermadjaja,B;Mulyawan,H;Dharmawan,R;Mahawan,T;Hutagaol,NM;Miswati,Y;Srihanto,EA;Hartawan,DHW;Hendrawati,F;Riyadi,A;Deswarni;TriHarsono,A;Hartaningsih,N;Azhar,M;Stegemen,A;McGrane,J;Rasa,FST |
| EPI_ISL_266802 | A/chicken/Subang/08160018-002/2016 | 2016-01-08 | Not listed | Other Database Import | Wibawa,H;Poermadjaja,B;Mulyawan,H;Dharmawan,R;Mahawan,T;Hutagaol,NM;Miswati,Y;Srihanto,EA;Hartawan,DHW;Hendrawati,F;Riyadi,A;Deswarni;TriHarsono,A;Hartaningsih,N;Azhar,M;Stegemen,A;McGrane,J;Rasa,FST |
| EPI_ISL_266801 | A/chicken/Kuningan/08160178-003/2016 | 2016-03-18 | Not listed | Other Database Import | Wibawa,H;Poermadjaja,B;Mulyawan,H;Dharmawan,R;Mahawan,T;Hutagaol,NM;Miswati,Y;Srihanto,EA;Hartawan,DHW;Hendrawati,F;Riyadi,A;Deswarni;TriHarsono,A;Hartaningsih,N;Azhar,M;Stegemen,A;McGrane,J;Rasa,FST |
| EPI_ISL_266792 | A/chicken/Pati/04160433/2016 | 2016-03-30 | Not listed | Other Database Import | Wibawa,H;Poermadjaja,B;Mulyawan,H;Dharmawan,R;Mahawan,T;Hutagaol,NM;Miswati,Y;Srihanto,EA;Hartawan,DHW;Hendrawati,F;Riyadi,A;Deswarni;TriHarsono,A;Hartaningsih,N;Azhar,M;Stegemen,A;McGrane,J;Rasa,FST |
| EPI_ISL_266791 | A/chicken/Kediri/04160512/2016 | 2016-04-05 | Not listed | Other Database Import | Wibawa,H;Poermadjaja,B;Mulyawan,H;Dharmawan,R;Mahawan,T;Hutagaol,NM;Miswati,Y;Srihanto,EA;Hartawan,DHW;Hendrawati,F;Riyadi,A;Deswarni;TriHarsono,A;Hartaningsih,N;Azhar,M;Stegemen,A;McGrane,J;Rasa,FST |
| EPI_ISL_261634 | A/chicken/Republic of Macedonia/AR1167-L02131/2017 | 2017-01-21 | University “Ss. Cyril and Methodius” , Veterinary institute, Department of Avian diseases | Friedrich-Loeffler-Institut | Dodovski,A |
| EPI_ISL_258596 | A/chicken/Iran/Tehran-F-2/2016 | 2016-11-21 | Not listed | Other Database Import | Ghalyanchilangeroudi,A;Ghafouri,SA;Maghsoudloo,H;Khaltabadifarahani,R;Abdolahi,H;Tehrani,F;Fallah,MH |
| EPI_ISL_258595 | A/chicken/Iran/Tehran-F-1/2016 | 2016-11-21 | Not listed | Other Database Import | Ghalyanchilangeroudi,A;Ghafouri,SA;Maghsoudloo,H;Khaltabadifarahani,R;Abdolahi,H;Tehrani,F;Fallah,MH |
| EPI_ISL_257447 | A/chicken/Chiba/1-2C/2017 | 2017-03-23 | National Institute of Animal Health | National Institute of Animal Health | Mine,J |
| EPI_ISL_257445 | A/chicken/Chiba/1-1T/2017 | 2017-03-23 | National Institute of Animal Health | National Institute of Animal Health | Mine,J |
| EPI_ISL_257444 | A/chicken/Chiba/1-2T/2017 | 2017-03-23 | National Institute of Animal Health | National Institute of Animal Health | Mine,J |
| EPI_ISL_257441 | A/chicken/Chiba/1-3C/2017 | 2017-03-23 | National Institute of Animal Health | National Institute of Animal Health | Mine,J |
| EPI_ISL_257438 | A/chicken/Miyagi/1-5C/2017 | 2017-03-23 | National Institute of Animal Health | National Institute of Animal Health | Mine,J |
| EPI_ISL_257004 | A/chicken/Czech Republic/206-17_2/2017 | 2017-01-06 | Not listed | Other Database Import | Nagy,A |
| EPI_ISL_256398 | A/chicken/Taiwan/x37/2016 | 2016-12-21 | Not listed | Animal Health Research Institute | Yu-Ju,Lin;Li-Hsuan,Chen;Wan-Chen,Li;Yen-Ping,Chen;Yu-Pin,Liu;Fan,Lee;Wen-Jane,Tu |
| EPI_ISL_256396 | A/chicken/Taiwan/u7/2016 | 2016-09-30 | Not listed | Animal Health Research Institute | Yu-Ju,Lin;Li-Hsuan,Chen;Wan-Chen,Li;Yen-Ping,Chen;Yu-Pin,Liu;Fan,Lee;Wen-Jane,Tu |
| EPI_ISL_256308 | A/chicken/Italy/17VIR1751-3/2017 | 2017-02-27 | Istituto Zooprofilattico Sperimentale Delle Venezie | Istituto Zooprofilattico Sperimentale Delle Venezie | Zecchin,B;Fusaro,A;Milani,A;Schivo,A;Salviato,A;Zamperin,G;Marciano,S;Ormelli,S;Terregino,C;Monne,I |
| EPI_ISL_256307 | A/chicken/Italy/17VIR1684-2/2017 | 2017-02-27 | Istituto Zooprofilattico Sperimentale Delle Venezie | Istituto Zooprofilattico Sperimentale Delle Venezie | Zecchin,B;Fusaro,A;Milani,A;Schivo,A;Salviato,A;Zamperin,G;Marciano,S;Ormelli,S;Terregino,C;Monne,I |
| EPI_ISL_255184 | A/chicken/Italy/17VIR653-12/2017 | 2017-01-25 | Istituto Zooprofilattico Sperimentale Delle Venezie | Istituto Zooprofilattico Sperimentale Delle Venezie | Zecchin,B;Fusaro,A;Milani,A;Schivo,A;Salviato,A;Zamperin,G;Marciano,S;Ormelli,S;Terregino,C;Monne,I |
| EPI_ISL_253032 | A/chicken/Zhejiang/232/2016 | 2016-01-31 | Not listed | Other Database Import | Wu,H;Lu,R;Peng,X;Chen,B;Cheng,L;Wu,N |
| EPI_ISL_253031 | A/chicken/Zhejiang/217/2016 | 2016-01-31 | Not listed | Other Database Import | Wu,H;Lu,R;Peng,X;Chen,B;Cheng,L;Wu,N |
| EPI_ISL_253030 | A/chicken/Zhejiang/209/2016 | 2016-01-31 | Not listed | Other Database Import | Wu,H;Lu,R;Peng,X;Chen,B;Cheng,L;Wu,N |
| EPI_ISL_253029 | A/chicken/Zhejiang/194/2016 | 2016-01-31 | Not listed | Other Database Import | Wu,H;Lu,R;Peng,X;Chen,B;Cheng,L;Wu,N |
| EPI_ISL_252882 | A/chicken/Czech Republic/2764-17_2/2017 (H5N8) | 2017-02-24 | Not listed | State Veterinary Institute Prague | Nagy,Alexander |
| EPI_ISL_247403 | A/chicken/Saga/1-7T/2017 | 2017-02-04 | National Institute of Animal Health | National Institute of Animal Health | Takehiko,S |
| EPI_ISL_247399 | A/chicken/Saga/1-7C/2017 | 2017-02-04 | National Institute of Animal Health | National Institute of Animal Health | Takehiko,S |
| EPI_ISL_247398 | A/chicken/Saga/1-4T/2017 | 2017-02-04 | National Institute of Animal Health | National Institute of Animal Health | Takehiko,S |
| EPI_ISL_247396 | A/chicken/Saga/1-2T/2017 | 2017-02-04 | National Institute of Animal Health | National Institute of Animal Health | Takehiko,S |
| EPI_ISL_247395 | A/chicken/Saga/1-6C/2017 | 2017-02-04 | National Institute of Animal Health | National Institute of Animal Health | Takehiko,S |
| EPI_ISL_247393 | A/chicken/Saga/1-5C/2017 | 2017-02-04 | National Institute of Animal Health | National Institute of Animal Health | Takehiko,S |
| EPI_ISL_247382 | A/chicken/Saga/1-1C/2017 | 2017-02-04 | National Institute of Animal Health | National Institute of Animal Health | Takehiko,S |
| EPI_ISL_247378 | A/chicken/Saga/1-1T/2017 | 2017-02-04 | National Institute of Animal Health | National Institute of Animal Health | Takehiko,S |
| EPI_ISL_244533 | A/chicken/Taishun/TS90/2016 | 2016-02-19 | Not listed | Other Database Import | Chen,LJ;Tian,JH;Lin,XD;Liao,Y;Shi,M;Zhang,YZ |
| EPI_ISL_244532 | A/chicken/Taishun/TS12/2016 | 2016-02-19 | Not listed | Other Database Import | Chen,LJ;Tian,JH;Lin,XD;Liao,Y;Shi,M;Zhang,YZ |
| EPI_ISL_244531 | A/chicken/Hubei/ZYSJF38/2016 | 2016-02-21 | Not listed | Other Database Import | Chen,LJ;Tian,JH;Lin,XD;Liao,Y;Shi,M;Zhang,YZ |
| EPI_ISL_244530 | A/chicken/Hubei/ZYSJF22/2016 | 2016-02-21 | Not listed | Other Database Import | Chen,LJ;Tian,JH;Lin,XD;Liao,Y;Shi,M;Zhang,YZ |
| EPI_ISL_244529 | A/chicken/Hubei/ZYSJF16/2016 | 2016-02-21 | Not listed | Other Database Import | Chen,LJ;Tian,JH;Lin,XD;Liao,Y;Shi,M;Zhang,YZ |
| EPI_ISL_244528 | A/chicken/Hubei/ZYSJF11/2016 | 2016-02-21 | Not listed | Other Database Import | Chen,LJ;Tian,JH;Lin,XD;Liao,Y;Shi,M;Zhang,YZ |
| EPI_ISL_243687 | A/chicken/Miyazaki/2-3C/2017 | 2017-01-24 | Not listed | National Institute of Animal Health | TakehikoSaito |
| EPI_ISL_243683 | A/chicken/Miyazaki/2-1C/2017 | 2017-01-24 | Not listed | National Institute of Animal Health | TakehikoSaito |
| EPI_ISL_243681 | A/chicken/Miyazaki/2-4C/2017 | 2017-01-24 | Not listed | National Institute of Animal Health | TakehikoSaito |
| EPI_ISL_243680 | A/chicken/Miyazaki/2-1T/2017 | 2017-01-24 | Not listed | National Institute of Animal Health | TakehikoSaito |
| EPI_ISL_243673 | A/chicken/Miyazaki/2-2C/2017 | 2017-01-24 | Not listed | National Institute of Animal Health | TakehikoSaito |
| EPI_ISL_242666 | A/chicken/Kumamoto/1-6C/2016 | 2016-12-27 | Not listed | National Institute of Animal Health | Saito,T;Takemae,N |
| EPI_ISL_242414 | A/chicken/Gifu/1-2C/2017 | 2017-01-14 | Not listed | National Institute of Animal Health | Saito,T;Uchida,Y |
| EPI_ISL_242413 | A/chicken/Gifu/1-8C/2017 | 2017-01-14 | Not listed | National Institute of Animal Health | Saito,T;Uchida,Y |
| EPI_ISL_242412 | A/chicken/Gifu/1-9C/2017 | 2017-01-14 | Not listed | National Institute of Animal Health | Saito,T;Uchida,Y |
| EPI_ISL_242411 | A/chicken/Gifu/1-6T/2017 | 2017-01-14 | Not listed | National Institute of Animal Health | Saito,T;Uchida,Y |
| EPI_ISL_242407 | A/chicken/Gifu/1-2T/2017 | 2017-01-14 | Not listed | National Institute of Animal Health | Saito,T;Uchida,Y |
| EPI_ISL_242404 | A/chicken/Gifu/1-10C/2017 | 2017-01-14 | Not listed | National Institute of Animal Health | Saito,T;Uchida,Y |
| EPI_ISL_242401 | A/chicken/Gifu/1-1T/2017 | 2017-01-14 | Not listed | National Institute of Animal Health | Saito,T;Uchida,Y |
| EPI_ISL_241779 | A/chicken/Kumamoto/1-5T/2016 | 2016-12-27 | Not listed | National Institute of Animal Health | Saito,T;Takemae,N |
| EPI_ISL_241766 | A/chicken/Kumamoto/1-5C/2016 | 2016-12-27 | Not listed | National Institute of Animal Health | Saito,T;Takemae,N |
| EPI_ISL_241765 | A/chicken/Kumamoto/1-4T/2016 | 2016-12-27 | Not listed | National Institute of Animal Health | Saito,T;Takemae,N |
| EPI_ISL_241764 | A/chicken/Kumamoto/1-7C/2016 | 2016-12-27 | Not listed | National Institute of Animal Health | Saito,T;Takemae,N |
| EPI_ISL_241763 | A/chicken/Kumamoto/1-6T/2016 | 2016-12-27 | Not listed | National Institute of Animal Health | Saito,T;Takemae,N |
| EPI_ISL_241758 | A/chicken/Miyazaki/1-5C/2016 | 2016-12-19 | Not listed | National Institute of Animal Health | Saito,T;Takemae,N |
| EPI_ISL_241751 | A/chicken/Kumamoto/1-4C/2016 | 2016-12-27 | Not listed | National Institute of Animal Health | Saito,T;Takemae,N |
| EPI_ISL_241745 | A/chicken/Kumamoto/1-7T/2016 | 2016-12-27 | Not listed | National Institute of Animal Health | Saito,T;Takemae,N |
| EPI_ISL_240624 | A/chicken/Miyazaki/1-6C/2016 | 2016-12-19 | National Institute of Animal Health | National Institute of Animal Health | Uchida,U |
| EPI_ISL_240622 | A/chicken/Miyazaki/1-6T/2016 | 2016-12-19 | National Institute of Animal Health | National Institute of Animal Health | Uchida,Y |
| EPI_ISL_240618 | A/chicken/Hokkaido/1-3-7C/2016 | 2016-12-16 | National Institute of Animal Health | National Institute of Animal Health | Uchida,Y |
| EPI_ISL_240599 | A/chicken/Hokkaido/1-3-7T/2016 | 2016-12-16 | National Institute of Animal Health | National Institute of Animal Health | Uchida,Y |
| EPI_ISL_240530 | A/chicken/Niigata/1-3C/2016 | 2016-11-28 | National Institute of Animal Health | National Institute of Animal Health | Uchida,Y |
| EPI_ISL_239427 | A/chicken/Niigata/2-5C/2016 | 2016-11-30 | National Institute of Animal Health | National Institute of Animal Health | Uchida,Y |
| EPI_ISL_239425 | A/chicken/Niigata/2-6T/2016 | 2016-11-30 | National Institute of Animal Health | National Institute of Animal Health | Uchida,Y |
| EPI_ISL_233723 | A/chicken/Niger/16VIR2650-7/2016 | 2016 | Laboratoire Central de l’Elevage (LABOCEL) | Istituto Zooprofilattico Sperimentale Delle Venezie | Bangana,I;Zecchin,B;Fusaro,A;Issoufou,M;Alassane,A;Milani,A;Schivo,A;Salviato,A;Marciano,S;Ormelli,S;Monne,I |
| EPI_ISL_232043 | A/chicken/Ghana/16VIR4304-13/2016 | 2016-04-18 | Veterinary Services Directorate, Ministry of Food and Agriculture | Istituto Zooprofilattico Sperimentale Delle Venezie | Awuni,J;Aniwa,B;Fusaro,A;Zecchin,B;Schivo,A;Salviato,A;Milani,A;Marciano,S;Ormelli,S;Monne,I |
| EPI_ISL_284778 | A/chicken/Bangladesh/30065/2016 | 2016-05-27 | Not listed | Other Database Import | Barman,S;Turner,JC;Hasan,MK;Akhtar,S;Franks,J;El-Shesheny,R;Walker,D;Seiler,P;Friedman,K;Kercher,L;Kayali,G;Jones-Engel,L;McKenzie,P;Krauss,S;Webby,RJ;Feeroz,MM;Webster,RG |
| EPI_ISL_278201 | A/chicken/Belgium/5990/2017 | 2017-06-10 | Not listed | Other Database Import | Steensels,M;Lambrecht,B;Vandenbussche,F;VanBorm,S |
| EPI_ISL_278200 | A/Brahma chicken/Belgium/6153/2017 | 2017-06-15 | Not listed | Other Database Import | Steensels,M;Lambrecht,B;Vandenbussche,F;VanBorm,S |
| EPI_ISL_278026 | A/Chicken/Huizhou/16274/2016(H5N6) | 2016-03-01 | South China Agricultural University | South China Agricultural University | Weixin,J |
| EPI_ISL_275288 | A/chicken/Tatarstan/88/2017 | 2017-05-08 | Not listed | State Research Center of Virology and Biotechnology (VECTOR) | Susloparov,I;Goncharova,N;Kolosova,N;Marchenko,V;Ryzhikov,A |
| EPI_ISL_275287 | A/chicken/Rostov-on-Don/44/2017 | 2017-04-18 | Not listed | State Research Center of Virology and Biotechnology (VECTOR) | Susloparov,I;Goncharova,N;Kolosova,N;Marchenko,V;Ryzhikov,A |
| EPI_ISL_268993 | A/chicken/Czech Republic/4231-17_2/2017 (H5N8) | 2017-03-21 | State Veterinary Institute Prague | State Veterinary Institute Prague | Nagy,A |
| EPI_ISL_268992 | A/chicken/Czech Republic/3507-17/2017 (H5N8) | 2017-02-28 | State Veterinary Institute Prague | State Veterinary Institute Prague | Nagy,A |
| EPI_ISL_268991 | A/chicken/Czech Republic/2822-17_2/2017 (H5N8) | 2017-02-27 | State Veterinary Institute Prague | State Veterinary Institute Prague | Nagy,A |
| EPI_ISL_268984 | A/chicken/Czech Republic/2677-17_2/2017 (H5N8) | 2017-02-22 | State Veterinary Institute Prague | State Veterinary Institute Prague | Nagy,A |
| EPI_ISL_268982 | A/chicken/Czech Republic/2514-17/2017 (H5N8) | 2017-02-19 | State Veterinary Institute Prague | State Veterinary Institute Prague | Nagy,A |
| EPI_ISL_268980 | A/chicken/Czech Republic/2216-17_1/2017 (H5N8) | 2017-02-11 | State Veterinary Institute Prague | State Veterinary Institute Prague | Nagy,A |
| EPI_ISL_268975 | A/chicken/Czech Republic/1953-17/2017 (H5N8) | 2017-02-08 | State Veterinary Institute Prague | State Veterinary Institute Prague | Nagy,A |
| EPI_ISL_268968 | A/chicken/Czech Republic/1689-17/2017 (H5N8) | 2017-01-04 | State Veterinary Institute Prague | State Veterinary Institute Prague | Nagy,A |
| EPI_ISL_268967 | A/chicken/Czech Republic/1688-17_1/2017 (H5N8) | 2017-02-04 | State Veterinary Institute Prague | State Veterinary Institute Prague | Nagy,A |
| EPI_ISL_268966 | A/chicken/Czech Republic/1687-17_2/2017 (H5N8) | 2017-02-04 | State Veterinary Institute Prague | State Veterinary Institute Prague | Nagy,A |
| EPI_ISL_268963 | A/chicken/Czech Republic/1675-17_2/2017 (H5N8) | 2017-02-03 | State Veterinary Institute Prague | State Veterinary Institute Prague | Nagy,A |
| EPI_ISL_268957 | A/chicken/Czech Republic/1465-17/2017 (H5N8) | 2017-01-30 | State Veterinary Institute Prague | State Veterinary Institute Prague | Nagy,A |
| EPI_ISL_268625 | A/Ch/NL-Hiaure/16016112-001-005/2016 | 2016-12-17 | Wageningen Bioveterinary Research | Wageningen Bioveterinary Research | Beerens,N;Heutink,R;Harders,F;Verschuren-Pritz,S;Bossers,A;Koch,G;Bergervoet,S |
| EPI_ISL_268623 | A/Ch/NL-Boven Leeuwen/16016151-006-010/2016 | 2016-12-19 | Wageningen Bioveterinary Research | Wageningen Bioveterinary Research | Beerens,N;Heutink,R;Harders,F;Verschuren-Pritz,S;Bossers,A;Koch,G;Bergervoet,S |
| EPI_ISL_268622 | A/Ch/NL-Abbega/X16015736/2016 | 2016-12-12 | Wageningen Bioveterinary Research | Wageningen Bioveterinary Research | Beerens,N;Heutink,R;Harders,F;Verschuren-Pritz,S;Bossers,A;Koch,G;Bergervoet,S |
| EPI_ISL_266536 | A/chicken/Belgium/807/2017 | 2017-02-01 | Not listed | Other Database Import | Steensels,M;Lambrecht,B;Vandenbussche,F;VanBorm,S |
| EPI_ISL_261332 | A/chicken/Croatia/104/2017 | 2017-03-07 | Not listed | Croatian Veterinary Institute | Savić,Vladimir |
| EPI_ISL_256500 | A/chicken/Hokkaido/002/2017 | 2016-12-16 | Graduate School of Veterinary Medicine, Hokkaido University | Hokkaido University | MasatoshiOkamatsu |
| EPI_ISL_256300 | A/chicken/Sergiyev Posad/39/2017 | 2017-03-02 | Not listed | State Research Center of Virology and Biotechnology (VECTOR) | Susloparov,I;Goncharova,N;Kolosova,N;Marchenko,V;Ryzhikov,A |
| EPI_ISL_256299 | A/chicken/Sergiyev Posad/38/2017 | 2017-03-02 | Not listed | State Research Center of Virology and Biotechnology (VECTOR) | Susloparov,I;Goncharova,N;Kolosova,N;Marchenko,V;Ryzhikov,A |
| EPI_ISL_255916 | A/chicken/Poland/002/2017 | 2017-01-02 | Not listed | National Veterinary Research Institut Poland, PIWet-PIB | Swieton,E;Smietanka,K |
| EPI_ISL_255915 | A/chicken/Poland/114/2016 | 2016-12-28 | Not listed | National Veterinary Research Institut Poland, PIWet-PIB | Swieton,E;Smietanka,K |
| EPI_ISL_255205 | A/Chicken/Hungary/2496/2017 | 2017-01-24 | National Food Chain Safety Office Veterinary Diagnostic Directorate Laboratory for Molecular Biology | Central Agricultural Office Veterinary Diagnostic Directorate | Dan,A |
| EPI_ISL_254827 | A/chicken/Egypt/F12505C/2016 | 2016-01-23 | Not listed | Other Database Import | Rubrum,A;Jeevan,T;Kayali,G;Ali,MA;Kandeil,A;El-Shesheny,R;Webby,R |
| EPI_ISL_254821 | A/chicken/Egypt/N12643B/2016 | 2016-05-08 | Not listed | Other Database Import | Rubrum,A;Jeevan,T;Kayali,G;Ali,MA;Kandeil,A;El-Shesheny,R;Webby,R |
| EPI_ISL_254820 | A/chicken/Egypt/N12638D/2016 | 2016-05-08 | Not listed | Other Database Import | Rubrum,A;Jeevan,T;Kayali,G;Ali,MA;Kandeil,A;El-Shesheny,R;Webby,R |
| EPI_ISL_254819 | A/chicken/Egypt/F12505B/2016 | 2016-01-23 | Not listed | Other Database Import | Rubrum,A;Jeevan,T;Kayali,G;Ali,MA;Kandeil,A;El-Shesheny,R;Webby,R |
| EPI_ISL_254815 | A/chicken/Egypt/N12640A/2016 | 2016-05-08 | Not listed | Other Database Import | Rubrum,A;Jeevan,T;Kayali,G;Ali,MA;Kandeil,A;El-Shesheny,R;Webby,R |
| EPI_ISL_254725 | A/chicken/Korea/H903/2017 | 2017-02-08 | Animal and Plant Quarantine Agency (O-2015) | Animal and Plant Quarantine Agency (S-2026) | Lee,YJ;Lee,EK;Song,BM;Lee,YN |
| EPI_ISL_253037 | A/chicken/Wales/000023/2016 | 2016-12-31 | Animal and Plant Health Agency (APHA) | Animal and Plant Health Agency (APHA) | Seekings,James;Ellis,Richard;Brookes,SharonM;Reid,Scott;Essen,Stephen;Brown,IanH |
| EPI_ISL_250919 | A/chicken/Czech Republic/1896-17_1/2017 (H5N8) | 2017-02-07 | Not listed | State Veterinary Institute Prague | Nagy,Alexander |
| EPI_ISL_250887 | A/chicken/Czech Republic/55-17_1/2017 (H5N8) | 2017-01-02 | Not listed | State Veterinary Institute Prague | Nagy,A |
| EPI_ISL_248879 | A/chicken/Croatia/70/2017 | 2017-02-08 | Not listed | Croatian Veterinary Institute | Savić,Vladimir |
| EPI_ISL_247725 | A/chicken/Kalmykia/2643/2016 | 2016-11-21 | Not listed | State Research Center of Virology and Biotechnology (VECTOR) | Susloparov,I;Goncharova,N;Kolosova,N;Marchenko,V;Ryzhikov,A |
| EPI_ISL_247718 | A/chicken/Voronezh/18/2017 | 2017-01-06 | Not listed | State Research Center of Virology and Biotechnology (VECTOR) | Susloparov,I;Goncharova,N;Kolosova,N;Marchenko,V;Ryzhikov,A |
| EPI_ISL_243803 | A/chicken/Egypt/ZU118/2016 | 2016-02-26 | Not listed | Other Database Import | Hussein,A;Orabi,A;Saleh,AA;AbuEl-Magd,M;Iqbal,M |
| EPI_ISL_243802 | A/chicken/Egypt/ZU117/2016 | 2016-02-24 | Not listed | Other Database Import | Hussein,A;Orabi,A;Saleh,AA;AbuEl-Magd,M;Iqbal,M |
| EPI_ISL_243801 | A/chicken/Egypt/ZU116/2016 | 2016-02-20 | Not listed | Other Database Import | Hussein,A;Orabi,A;Saleh,AA;AbuEl-Magd,M;Iqbal,M |
| EPI_ISL_243800 | A/chicken/Egypt/ZU108/2016 | 2016-02-20 | Not listed | Other Database Import | Hussein,A;Orabi,A;Saleh,AA;AbuEl-Magd,M;Iqbal,M |
| EPI_ISL_243799 | A/chicken/Egypt/ZU105/2016 | 2016-02-20 | Not listed | Other Database Import | Hussein,A;Orabi,A;Saleh,AA;AbuEl-Magd,M;Iqbal,M |
| EPI_ISL_243798 | A/chicken/Egypt/ZU103/2016 | 2016-02-20 | Not listed | Other Database Import | Hussein,A;Orabi,A;Saleh,AA;AbuEl-Magd,M;Iqbal,M |
| EPI_ISL_243793 | A/chicken/Egypt/ZU30/2016 | 2016-02-15 | Not listed | Other Database Import | Hussein,A;Orabi,A;Saleh,AA;AbuEl-Magd,M;Iqbal,M |
| EPI_ISL_243792 | A/chicken/Egypt/ZU29/2016 | 2016-02-15 | Not listed | Other Database Import | Hussein,A;Orabi,A;Saleh,AA;AbuEl-Magd,M;Iqbal,M |
| EPI_ISL_243791 | A/chicken/Egypt/ZU28/2016 | 2016-02-15 | Not listed | Other Database Import | Hussein,A;Orabi,A;Saleh,AA;AbuEl-Magd,M;Iqbal,M |
| EPI_ISL_243790 | A/chicken/Egypt/ZU3/2016 | 2016-02-15 | Not listed | Other Database Import | Hussein,A;Orabi,A;Saleh,AA;AbuEl-Magd,M;Iqbal,M |
| EPI_ISL_240540 | A/chicken/Croatia/103/2016 | 2016-12-27 | Not listed | Croatian Veterinary Institute | Savić,Vladimir |
| EPI_ISL_240110 | A/chicken/Astrakhan/3131/2016 | 2016-12-13 | Not listed | State Research Center of Virology and Biotechnology (VECTOR) | Susloparov,I;Marchenko,V;Goncharova,N;Durymanov,A;Ilyicheva,T;Ryzhikov,A |
| EPI_ISL_240105 | A/chicken/Poland/85A/2016 | 2016-12-19 | Not listed | National Veterinary Research Institut Poland, PIWet-PIB | Swieton,E;Smietanka,K |
| EPI_ISL_240104 | A/chicken/Poland/79A/2016 | 2016-12-17 | Not listed | National Veterinary Research Institut Poland, PIWet-PIB | Swieton,E;Smietanka,K |
| EPI_ISL_239261 | A/chicken/Korea/HN1/2016(H5N6) | 2016-11-16 | Not listed | Animal and Plant Quarantine Agency (S-2026) | Lee,YJ |
| EPI_ISL_239260 | A/chicken/Korea/H23/2016(H5N6) | 2016-11-20 | Animal and Plant Quarantine Agency (O-2015) | Animal and Plant Quarantine Agency (S-2026) | Lee,YJ |
| EPI_ISL_238894 | A/Chicken/Sweden/SVA161122KU0453/SZ0209317/2016 | 2016-11-21 | National Veterinary Institute | National Veterinary Institute | Zohari,S |
| EPI_ISL_238893 | A/Chicken/Sweden/SVA161122KU0453/SZ0209316/2016 | 2016-11-21 | National Veterinary Institute | National Veterinary Institute | Zohari,S |
| EPI_ISL_238039 | A/chicken/Germany-SH/R8758/2016 | 2016-11-11 | Not listed | Friedrich-Loeffler-Institut | Starick,E |
| EPI_ISL_238038 | A/chicken/Germany-MV/R8790/2016 | 2016-11-11 | Not listed | Friedrich-Loeffler-Institut | Starick,E |
| EPI_ISL_205833 | A/chicken/Scotland/532/2016 | 2016-01-09 | Animal and Plant Health Agency (APHA) | Animal and Plant Health Agency (APHA) | Seekings,A;Ellis,R;McGinn,N;Mynn,J;Coward,V;Reid,S;Banks,J;Brookes,S;Brown,I |
| EPI_ISL_308807 | A/duck/Cameroon/17RS1661-3/2017 | 2017-01 | Not listed | Other Database Import | Wade,A;Zecchin,B;Jumbo,SD;Fusaro,A;Taiga,T;Bianco,A;PouemeN,R;Salomoni,A;FeussomKameni,JM;Zamperin,G;Kazi,JP;Nenkam,R;Foupouapouognigni,Y;Abdoulkadiri,S;Yaya,A;Monne,I |
| EPI_ISL_308802 | A/duck/Democratic Republic of the Congo/17RS882-29/2017 | 2017-05-15 | Not listed | Other Database Import | Twabela,A;Zecchin,B;Tshilenge,G;Sakoda,Y;Kone,P;Zamperin,G;Drago,A;Monne,I |
| EPI_ISL_305601 | A/duck/Cameroon/16VIR3791-21/2016 | 2016-05-30 | Not listed | Other Database Import | Wade,A;Monne,I;Taiga;Fouda,M;Abari,M;Feussom,JM;Zecchin,B;Fusaro,A;Milani,A;Terregino,C |
| EPI_ISL_300920 | A/domestic_duck/112/2016 | 2016-12-28 | Not listed | National Veterinary Research Institut Poland, PIWet-PIB | Swieton,E;Smietanka,K |
| EPI_ISL_300693 | A/domestic_duck/Poland/47/2017 | 2017-01-16 | National Veterinary Research Institut Poland, PIWet-PIB | National Veterinary Research Institut Poland, PIWet-PIB | Swieton,E;Smietanka,K |
| EPI_ISL_297136 | A/duck/Egypt/HASHF1/2016 | 2016-01 | Not listed | Other Database Import | Tolba,HMN;AbouElez,RMM;Elsohaby,I;Ahmed,HA |
| EPI_ISL_295814 | A/duck/Vietnam/HU5-1572/2016 | 2016-08-15 | Not listed | Other Database Import | Chu,DH;Nguyen,LT;Okamatsu,M;Matsuno,K;Sakoda,Y;Jizou,M |
| EPI_ISL_295795 | A/duck/Vietnam/HU8-1817/2017 | 2017-08-21 | Not listed | Other Database Import | Nguyen,LT;Chu,DH;Okamatsu,M;Matsuno,K;Sakoda,Y;Jizou,M |
| EPI_ISL_295794 | A/duck/Vietnam/HU8-1621/2017 | 2017-08-21 | Not listed | Other Database Import | Nguyen,LT;Chu,DH;Okamatsu,M;Matsuno,K;Sakoda,Y;Jizou,M |
| EPI_ISL_295755 | A/duck/Vietnam/HU8-1561/2017 | 2017-08-21 | Not listed | Other Database Import | Nguyen,LT;Chu,DH;Okamatsu,M;Matsuno,K;Sakoda,Y;Jizou,M |
| EPI_ISL_287907 | A/Duck/Netherlands/17017237-001-005/2017 | 2017-12-07 | Wageningen Bioveterinary Research | Wageningen Bioveterinary Research | Beerens,N;Heutink,R;Harders,F;Verschuren-Pritz,S;Bossers,A;Koch,G;Bergervoet,S |
| EPI_ISL_285625 | A/Duck/South_Africa/S2017/08_0481_P2/2017 | 2017-08-24 | Western Cape Provincial Veterinary Laboratory | National Institute of Communicable Diseases | Treurnicht,FK |
| EPI_ISL_285619 | A/chicken/Egypt/Kafr-Elshiekh-18/2017 | 2017 | Not listed | Friedrich-Loeffler-Institut | Salaheldin,AH;AbdEl-Hamid,HS;Elbestawy,AR;Hafez,MH;Veits,J;Mettenleiter,TC;Abdelwhab,EM' |
| EPI_ISL_283966 | A/Duck/Yunnan/YN-9/2016 (H5N6) | 2016-01-12 | South China Agricultural University | South China Agricultural University | Jiahao,Z;Guangjie,L;Ronghua,Z;Hexing,W;Guanming,S;Bo,L;Wenbao,Q;Ming,L |
| EPI_ISL_283702 | A/Duck/Yunnan/YN-2/2016 (H5N6) | 2016-06-22 | South China Agricultural University | South China Agricultural University | Jiahao,Z;Guangjie,L;Ronghua,Z;Hexing,W;Guanming,S;Bo,L;Wenbao,Q;Ming,L |
| EPI_ISL_279014 | A/duck/Kagoshima/KU-d42/2016 | 2016-11-20 | Not listed | Kagoshima University | MakatoOzawa |
| EPI_ISL_279013 | A/duck/Kagoshima/KU-d35/2016 | 2016-11-20 | Not listed | Kagoshima University | MakatoOzawa |
| EPI_ISL_268634 | A/Dk/NL-Stolwijk/16016291-016-020/2016 | 2016-12-21 | Wageningen Bioveterinary Research | Wageningen Bioveterinary Research | Beerens,N;Heutink,R;Harders,F;Verschuren-Pritz,S;Bossers,A;Koch,G;Bergervoet,S |
| EPI_ISL_268632 | A/Dk/NL-Kamperveen/16016104-001-005/2016 | 2016-12-15 | Wageningen Bioveterinary Research | Wageningen Bioveterinary Research | Beerens,N;Heutink,R;Harders,F;Verschuren-Pritz,S;Bossers,A;Koch,G;Bergervoet,S |
| EPI_ISL_268631 | A/Dk/NL-Biddinghuizen/16015145-021-025/2016 | 2016-12-01 | Wageningen Bioveterinary Research | Wageningen Bioveterinary Research | Beerens,N;Heutink,R;Harders,F;Verschuren-Pritz,S;Bossers,A;Koch,G;Bergervoet,S |
| EPI_ISL_268630 | A/Dk/NL-Biddinghuizen/16015083-016-020/2016 | 2016-11-30 | Wageningen Bioveterinary Research | Wageningen Bioveterinary Research | Beerens,N;Heutink,R;Harders,F;Verschuren-Pritz,S;Bossers,A;Koch,G;Bergervoet,S |
| EPI_ISL_268513 | A/duck/Egypt/F446/2017 | 2017-04-06 | National Laboratory for Veterinary Quality Control on Poultry production- Animal Health Research Inistitute | Animal Health Research Institute | Nahed,Yehia;Mahmoud,Naguib;Naglaa,Hagag;Mohamed,El-Husseiny;Zainab,Mosaad;Mohamed,Hassan;AbdelSatar,Arafa' |
| EPI_ISL_268021 | A/duck/Egypt/SS19/2017 | 2017-01-12 | National Laboratory for Veterinary Quality Control on Poultry production- Animal Health Research Inistitute | Animal Health Research Institute | Nahed,Yehia;Mahmoud,Naguib;Naglaa,Hagag;Mohamed,El-Husseiny;Zainab,Mosaad;Mohamed,Hassan;AbdelSatar,Arafa' |
| EPI_ISL_266803 | A/duck/Sukoharjo/04160290/2016 | 2016-03-03 | Not listed | Other Database Import | Wibawa,H;Poermadjaja,B;Mulyawan,H;Dharmawan,R;Mahawan,T;Hutagaol,NM;Miswati,Y;Srihanto,EA;Hartawan,DHW;Hendrawati,F;Riyadi,A;Deswarni;TriHarsono,A;Hartaningsih,N;Azhar,M;Stegemen,A;McGrane,J;Rasa,FST |
| EPI_ISL_266795 | A/duck/Sidrap/07160336-3/2016 | 2016-04-04 | Not listed | Other Database Import | Wibawa,H;Poermadjaja,B;Mulyawan,H;Dharmawan,R;Mahawan,T;Hutagaol,NM;Miswati,Y;Srihanto,EA;Hartawan,DHW;Hendrawati,F;Riyadi,A;Deswarni;TriHarsono,A;Hartaningsih,N;Azhar,M;Stegemen,A;McGrane,J;Rasa,FST |
| EPI_ISL_266790 | A/duck/Lamongan/04160434/2016 | 2016-03-30 | Not listed | Other Database Import | Wibawa,H;Poermadjaja,B;Mulyawan,H;Dharmawan,R;Mahawan,T;Hutagaol,NM;Miswati,Y;Srihanto,EA;Hartawan,DHW;Hendrawati,F;Riyadi,A;Deswarni;TriHarsono,A;Hartaningsih,N;Azhar,M;Stegemen,A;McGrane,J;Rasa,FST |
| EPI_ISL_266789 | A/duck/Lumajang/04160417/2016 | 2016-03-29 | Not listed | Other Database Import | Wibawa,H;Poermadjaja,B;Mulyawan,H;Dharmawan,R;Mahawan,T;Hutagaol,NM;Miswati,Y;Srihanto,EA;Hartawan,DHW;Hendrawati,F;Riyadi,A;Deswarni;TriHarsono,A;Hartaningsih,N;Azhar,M;Stegemen,A;McGrane,J;Rasa,FST |
| EPI_ISL_260799 | A/pintail/Tokyo/1/2017 | 2017-02-03 | Not listed | Hokkaido University | MasatoshiOkamatsu |
| EPI_ISL_260780 | A/mallard/Iwate/19/2017 | 2017-02-17 | Not listed | Hokkaido University | MasatoshiOkamatsu |
| EPI_ISL_256818 | A/domestic duck/Germany-MV/R9764/2016 | 2016-11-21 | Not listed | Friedrich-Loeffler-Institut | Starick,E |
| EPI_ISL_252825 | A/duck/Hubei/ZYSYF2/2016 | 2016-02-21 | Not listed | Other Database Import | Chen,LJ;Tian,JH;Lin,XD;Liao,Y;Shi,M;Zhang,YZ |
| EPI_ISL_247724 | A/wild duck/Tatarstan/3059/2016 | 2016-10-02 | Not listed | State Research Center of Virology and Biotechnology (VECTOR) | Susloparov,I;Goncharova,N;Kolosova,N;Marchenko,V;Ryzhikov,A |
| EPI_ISL_240678 | A/domestic duck/Siberia/50K/2016 | 2016-10-10 | Not listed | Research Institute of Experimental and Clinical Medicine | Sharshov,KA;Kurskaya,OG;Alexeev,AY;Sobolev,IA;Alikina,TY;Kabilov,MR;Shestopalov,AM |
| EPI_ISL_240677 | A/domestic duck/Siberia/103/2016 | 2016-10-04 | Research Institute of Experimental and Clinical Medicine | Research Institute of Experimental and Clinical Medicine | Sharshov,KA;Kurskaya,OG;Alexeev,AY;Sobolev,IA;Alikina,TY;Kabilov,MR;Shestopalov,AM |
| EPI_ISL_240616 | A/duck/Ibaraki/22/2016 | 2016-12-14 | National Institute of Animal Health | National Institute of Animal Health | Uchida,Y |
| EPI_ISL_240533 | A/muscovy duck/Aomori/1-7T/2016 | 2016-11-28 | National Institute of Animal Health | National Institute of Animal Health | Uchida,Y |
| EPI_ISL_240532 | A/muscovy duck/Aomori/1-1T/2016 | 2016-11-28 | National Institute of Animal Health | National Institute of Animal Health | Uchida,Y |
| EPI_ISL_239433 | A/muscovy duck/Aomori/1-3T/2016 | 2016-11-28 | National Institute of Animal Health | National Institute of Animal Health | Uchida,Y |
| EPI_ISL_239431 | A/muscovy duck/Aomori/2-3T/2016 | 2016-12-02 | National Institute of Animal Health | National Institute of Animal Health | Uchida,Y |
| EPI_ISL_239428 | A/muscovy duck/Aomori/2-2T/2016 | 2016-12-02 | National Institute of Animal Health | National Institute of Animal Health | Uchida,Y |
| EPI_ISL_239421 | A/muscovy duck/Aomori/1-2T/2016 | 2016-11-28 | National Institute of Animal Health | National Institute of Animal Health | Uchida,Y |
| EPI_ISL_239351 | A/duck/Hyogo/1/2016 | 2016-11-14 | Hyogo Prefectural Institute of Public Health and Consumer Sciences | National Institute of Infectious Diseases (NIID) | Nakauchi,Mina;Saito,Shinji;Takayama,Ikuyo;Kageyama,Tsutomu;Odagiri,Takato |
| EPI_ISL_237921 | A/wild duck/Poland/82A/2016 | 2016-11-02 | Not listed | National Veterinary Research Institut Poland, PIWet-PIB | Swieton,E;Smietanka,K |
| EPI_ISL_237792 | A/wild duck/Germany-BW/R8455/2016 | 2016-11-07 | Not listed | Friedrich-Loeffler-Institut | Starick,E |
| EPI_ISL_231747 | A/duck/Hubei/SZY250/2016 | 2016-04-21 | Not listed | Other Database Import | Yao,Y;Chen,J |
| EPI_ISL_231684 | A/wild duck/Tyva/35/2016 | 2016-05-25 | State Research Center of Virology and Biotechnology (VECTOR) | WHO National Influenza Centre Russian Federation | Fadeev,A;Komissarov,A;Egorova,A;Sintsova,K;Musaeva,T;Susloparov,I;Marchenko,V;Ryzhikov,A |
| EPI_ISL_221716 | A/duck/Guangdong/01.01 SZSGXJK007-G/2016 | 2016-01-01 | Institute of Microbiology, Chinese Academy of Sciences | Institute of Microbiology, Chinese Academy of Sciences | Bi,Y |
| EPI_ISL_221714 | A/duck/Guangdong/01.01 SZSGXJK006-Y/2016 | 2016-01-01 | Institute of Microbiology, Chinese Academy of Sciences | Institute of Microbiology, Chinese Academy of Sciences | Bi,Y |
| EPI_ISL_221708 | A/duck/Guangdong/01.01 SZSGXJK002-G/2016 | 2016-01-01 | Institute of Microbiology, Chinese Academy of Sciences | Institute of Microbiology, Chinese Academy of Sciences | Bi,Y |
| EPI_ISL_221707 | A/duck/Guangdong/01.01 SZSGXJK002-Y/2016 | 2016-01-01 | Institute of Microbiology, Chinese Academy of Sciences | Institute of Microbiology, Chinese Academy of Sciences | Bi,Y |
| EPI_ISL_221706 | A/duck/Guangdong/01.01 SZSGXJK001-G/2016 | 2016-01-01 | Institute of Microbiology, Chinese Academy of Sciences | Institute of Microbiology, Chinese Academy of Sciences | Bi,Y |
| EPI_ISL_221705 | A/duck/Guangdong/01.01 SZSGXJK001-Y /2016 | 2016-01-01 | Institute of Microbiology, Chinese Academy of Sciences | Institute of Microbiology, Chinese Academy of Sciences | Bi,Y |
| EPI_ISL_256497 | A/pintail/Hokkaido/X8/2016 | 2016-12-19 | Graduate School of Veterinary Medicine, Hokkaido University | Hokkaido University | MasatoshiOkamatsu |
| EPI_ISL_242903 | A/Northern Pintail/Tottori/b37/2016 | 2016-11-06 | Not listed | Other Database Import | Takakuwa,H |
| EPI_ISL_294055 | A/American wigeon/Ohio/16OS3736/2016 | 2016-10-22 | Not listed | Other Database Import | Killian,M;Franzen,K;Camp,P;Stuber,T;Robbe-Austerman,S;Lauterbach,S;Nolting,J;Bowman,A |
| EPI_ISL_280491 | A/shoveller duck/Shanghai/JDS90/2016 | 2016-11-03 | Not listed | Other Database Import | He,G;Zhou,L;Zhu,C;Shi,H;Li,X;Wu,D;Liu,J;Lv,J;Hu,C;Li,Z;Wang,Z;Wang,T |
| EPI_ISL_280488 | A/common teal/Shanghai/JDS81/2016 | 2016-11-03 | Not listed | Other Database Import | He,G;Zhou,L;Zhu,C;Shi,H;Li,X;Wu,D;Liu,J;Lv,J;Hu,C;Li,Z;Wang,Z;Wang,T |
| EPI_ISL_280487 | A/common teal/Shanghai/JDS76/2016 | 2016-11-03 | Not listed | Other Database Import | He,G;Zhou,L;Zhu,C;Shi,H;Li,X;Wu,D;Liu,J;Lv,J;Hu,C;Li,Z;Wang,Z;Wang,T |
| EPI_ISL_280485 | A/common teal/Shanghai/JDS36/2016 | 2016-11-03 | Not listed | Other Database Import | He,G;Zhou,L;Zhu,C;Shi,H;Li,X;Wu,D;Liu,J;Lv,J;Hu,C;Li,Z;Wang,Z;Wang,T |
| EPI_ISL_266425 | A/common teal/Korea/W559/2017 | 2017-01-04 | Not listed | Other Database Import | Kim,Y-I;Park,S-J;Kwon,H-I;Kim,E-H;Si,Y-J;Jeong,JH;Lee,I-W;Hiep,DN;Kwon,J-J;Choi,WS;Song,M-S;Kim,C-J;Choi,Y-K |
| EPI_ISL_266420 | A/common teal/Korea/W550/2016 | 2016-12-15 | Not listed | Other Database Import | Kim,Y-I;Park,S-J;Kwon,H-I;Kim,E-H;Si,Y-J;Jeong,JH;Lee,I-W;Hiep,DN;Kwon,J-J;Choi,WS;Song,M-S;Kim,C-J;Choi,Y-K |
| EPI_ISL_266419 | A/common teal/Korea/W548/2016 | 2016-12-15 | Not listed | Other Database Import | Kim,Y-I;Park,S-J;Kwon,H-I;Kim,E-H;Si,Y-J;Jeong,JH;Lee,I-W;Hiep,DN;Kwon,J-J;Choi,WS;Song,M-S;Kim,C-J;Choi,Y-K |
| EPI_ISL_266418 | A/common teal/Korea/W549/2016 | 2016-12-15 | Not listed | Other Database Import | Kim,Y-I;Park,S-J;Kwon,H-I;Kim,E-H;Si,Y-J;Jeong,JH;Lee,I-W;Hiep,DN;Kwon,J-J;Choi,WS;Song,M-S;Kim,C-J;Choi,Y-K |
| EPI_ISL_266417 | A/common teal/Korea/W547/2016 | 2016-12-15 | Not listed | Other Database Import | Kim,Y-I;Park,S-J;Kwon,H-I;Kim,E-H;Si,Y-J;Jeong,JH;Lee,I-W;Hiep,DN;Kwon,J-J;Choi,WS;Song,M-S;Kim,C-J;Choi,Y-K |
| EPI_ISL_243060 | A/teal/Tottori/1/2016 | 2016-11-15 | Not listed | Other Database Import | Soda,K;Usui,T;Ito,H;Ozaki,H;Murase,T;Yamaguchi,T;Ito,T |
| EPI_ISL_302824 | A/Eurasian wigeon/Netherlands/1/2018 | 2018-02-07 | Erasmus Medical Center | Erasmus Medical Center | Poen,MJ;Bestebroer,TM;Vuong,O;Scheuer,RD;Kelder,L;Fouchier,RAM |
| EPI_ISL_293501 | A/wigeon/Aichi/2301H025/2017 | 2017-01-03 | Not listed | Other Database Import | Soda,K;Usui,T;Ito,H;Ozaki,H;Yamaguchi,T;Ito,T |
| EPI_ISL_279024 | A/eurasian wigeon/Kagoshima/KU-21/2016 | 2016-11-23 | Not listed | Kagoshima University | MakatoOzawa |
| EPI_ISL_279023 | A/eurasian wigeon/Kagoshima/KU-20/2016 | 2016-11-23 | Not listed | Kagoshima University | MakatoOzawa |
| EPI_ISL_269703 | A/Eurasian_Wigeon/Netherlands/25/2016 | 2016-12-05 | Not listed | Erasmus Medical Center | Poen,MJ;VanDerJeugd,HP;Vuong,O;Scheuer,RD;Kleyheeg,E;Bestebroer,TM;Begeman,L;vandenBrand,JMA;Kuiken,T;Fouchier,RAM |
| EPI_ISL_269696 | A/Eurasian_Wigeon/Netherlands/23/2016 | 2016-12-05 | Erasmus Medical Center | Erasmus Medical Center | Poen,MJ;VanDerJeugd,HP;Vuong,O;Scheuer,RD;Kleyheeg,E;Bestebroer,TM;Begeman,L;vandenBrand,JMA;Kuiken,T;Fouchier,RAM |
| EPI_ISL_269596 | A/Eurasian_Wigeon/Netherlands/13/2016 | 2016-12-14 | Erasmus Medical Center | Erasmus Medical Center | Poen,MJ;VanDerJeugd,HP;Vuong,O;Scheuer,RD;Kleyheeg,E;Bestebroer,TM;Kuiken,T;Fouchier,RAM |
| EPI_ISL_269595 | A/Eurasian_Wigeon/Netherlands/12/2016 | 2016-12-14 | Erasmus Medical Center | Erasmus Medical Center | Poen,MJ;VanDerJeugd,HP;Vuong,O;Scheuer,RD;Kleyheeg,E;Bestebroer,TM;Kuiken,T;Fouchier,RAM |
| EPI_ISL_269593 | A/Eurasian_Wigeon/Netherlands/11/2016 | 2016-12-13 | Erasmus Medical Center | Erasmus Medical Center | Poen,MJ;VanDerJeugd,HP;Vuong,O;Scheuer,RD;Kleyheeg,E;Bestebroer,TM;Kuiken,T;Fouchier,RAM |
| EPI_ISL_269592 | A/Eurasian_Wigeon/Netherlands/8/2016 | 2016-12-09 | Erasmus Medical Center | Erasmus Medical Center | Poen,MJ;VanDerJeugd,HP;Vuong,O;Scheuer,RD;Kleyheeg,E;Bestebroer,TM;Begeman,L;vandenBrand,JMA;Kuiken,T;Fouchier,RAM |
| EPI_ISL_268937 | A/Eurasian_Wigeon/Netherlands/10/2016 | 2016-12-08 | Erasmus Medical Center | Erasmus Medical Center | Poen,MJ;VanDerJeugd,HP;Vuong,O;Scheuer,RD;Kleyheeg,E;Bestebroer,TM;Kuiken,T;Fouchier,RAM |
| EPI_ISL_268653 | A/Eur_Wig/NL-Zwolle/16015820-002/2016 | 2016-12-13 | Wageningen Bioveterinary Research | Wageningen Bioveterinary Research | Beerens,N;Heutink,R;Harders,F;Verschuren-Pritz,S;Bossers,A;Koch,G;Bergervoet,S |
| EPI_ISL_268651 | A/Eur_Wig/NL-Wormer/16016143-002/2016 | 2016-12-18 | Wageningen Bioveterinary Research | Wageningen Bioveterinary Research | Beerens,N;Heutink,R;Harders,F;Verschuren-Pritz,S;Bossers,A;Koch,G;Bergervoet,S |
| EPI_ISL_268650 | A/Eur_Wig/NL-West Graftdijk/16015746-003/2016 | 2016-12-12 | Wageningen Bioveterinary Research | Wageningen Bioveterinary Research | Beerens,N;Heutink,R;Harders,F;Verschuren-Pritz,S;Bossers,A;Koch,G;Bergervoet,S |
| EPI_ISL_268649 | A/Eur_Wig/NL-Walterswald/16015923-003/2016 | 2016-12-14 | Wageningen Bioveterinary Research | Wageningen Bioveterinary Research | Beerens,N;Heutink,R;Harders,F;Verschuren-Pritz,S;Bossers,A;Koch,G;Bergervoet,S |
| EPI_ISL_268648 | A/Eur_Wig/NL-Vianen/16015917-006/2016 | 2016-12-13 | Wageningen Bioveterinary Research | Wageningen Bioveterinary Research | Beerens,N;Heutink,R;Harders,F;Verschuren-Pritz,S;Bossers,A;Koch,G;Bergervoet,S |
| EPI_ISL_268646 | A/Eur_Wig/NL-Reeuwijk/16015903-003/2016 | 2016-12-13 | Wageningen Bioveterinary Research | Wageningen Bioveterinary Research | Beerens,N;Heutink,R;Harders,F;Verschuren-Pritz,S;Bossers,A;Koch,G;Bergervoet,S |
| EPI_ISL_268645 | A/Eur_Wig/NL-Leidschendam/16015697-007/2016 | 2016-12-10 | Wageningen Bioveterinary Research | Wageningen Bioveterinary Research | Beerens,N;Heutink,R;Harders,F;Verschuren-Pritz,S;Bossers,A;Koch,G;Bergervoet,S |
| EPI_ISL_268644 | A/Eur_Wig/NL-Leeuwarden/16015699-002/2016 | 2016-12-10 | Wageningen Bioveterinary Research | Wageningen Bioveterinary Research | Beerens,N;Heutink,R;Harders,F;Verschuren-Pritz,S;Bossers,A;Koch,G;Bergervoet,S |
| EPI_ISL_268643 | A/Eur_Wig/NL-Groningen/16015376-003/2016 | 2016-12-05 | Wageningen Bioveterinary Research | Wageningen Bioveterinary Research | Beerens,N;Heutink,R;Harders,F;Verschuren-Pritz,S;Bossers,A;Koch,G;Bergervoet,S |
| EPI_ISL_268642 | A/Eur_Wig/NL-Greonterp/16015653-001/2016 | 2016-12-08 | Wageningen Bioveterinary Research | Wageningen Bioveterinary Research | Beerens,N;Heutink,R;Harders,F;Verschuren-Pritz,S;Bossers,A;Koch,G;Bergervoet,S |
| EPI_ISL_268640 | A/Eur_Wig/NL-Ferwert/16015273-002/2016 | 2016-12-01 | Wageningen Bioveterinary Research | Wageningen Bioveterinary Research | Beerens,N;Heutink,R;Harders,F;Verschuren-Pritz,S;Bossers,A;Koch,G;Bergervoet,S |
| EPI_ISL_268639 | A/Eur_Wig/NL-Enumatil-Groningen/16015704-001/2016 | 2016-12-11 | Wageningen Bioveterinary Research | Wageningen Bioveterinary Research | Beerens,N;Heutink,R;Harders,F;Verschuren-Pritz,S;Bossers,A;Koch,G;Bergervoet,S |
| EPI_ISL_268638 | A/Eur_Wig/NL-Drieborg (Dollard)/16015513-001/2016 | 2016-12-06 | Wageningen Bioveterinary Research | Wageningen Bioveterinary Research | Beerens,N;Heutink,R;Harders,F;Verschuren-Pritz,S;Bossers,A;Koch,G;Bergervoet,S |
| EPI_ISL_268636 | A/Eur_Wig/NL-De Waal (Texel)/16014891-003/2016 | 2016-11-27 | Wageningen Bioveterinary Research | Wageningen Bioveterinary Research | Beerens,N;Heutink,R;Harders,F;Verschuren-Pritz,S;Bossers,A;Koch,G;Bergervoet,S |
| EPI_ISL_268635 | A/Eur_Wig/NL-Akkrum/16015817-003/2016 | 2016-12-13 | Wageningen Bioveterinary Research | Wageningen Bioveterinary Research | Beerens,N;Heutink,R;Harders,F;Verschuren-Pritz,S;Bossers,A;Koch,G;Bergervoet,S |
| EPI_ISL_262055 | A/eurasian wigeon/Germany-NI/AR249-L02143/2017 | 2017-01-04 | Not listed | Friedrich-Loeffler-Institut | Pohlmann,A |
| EPI_ISL_255914 | A/Eurasian Wigeon/Netherlands/9/2016 | 2016-12-04 | Erasmus Medical Center | Erasmus Medical Center | Poen,MJ;VanDerJeugd,HP;Vuong,O;Scheuer,RD;Kleyheeg,E;Lexmond,P;Eggink,WD;Müskens,GJDM;Bestebroer,TM;Koopmans,MPG;Kuiken,T;Fouchier,RAM |
| EPI_ISL_255912 | A/Eurasian Wigeon/Netherlands/4/2016 | 2016-12-09 | Erasmus Medical Center | Erasmus Medical Center | Poen,MJ;VanDerJeugd,HP;Vuong,O;Scheuer,RD;Kleyheeg,E;Lexmond,P;Eggink,WD;Müskens,GJDM;Bestebroer,TM;Koopmans,MPG;Kuiken,T;Fouchier,RAM |
| EPI_ISL_255911 | A/Eurasian Wigeon/Netherlands/2/2016 | 2016-12-05 | Erasmus Medical Center | Erasmus Medical Center | Poen,MJ;VanDerJeugd,HP;Vuong,O;Scheuer,RD;Kleyheeg,E;Lexmond,P;Eggink,WD;Müskens,GJDM;Bestebroer,TM;Koopmans,MPG;Kuiken,T;Fouchier,RAM |
| EPI_ISL_306664 | A/duck/Bangladesh/32247/2017 | 2017-03-29 | Not listed | Other Database Import | Barman,S;Turner,JC;Hasan,MK;Akhtar,S;Franks,J;El-Shesheny,R;Walker,D;Seiler,P;Friedman,K;Kercher,L;Kayali,G;Jones-Engel,L;McKenzie,P;Krauss,S;Webby,RJ;Feeroz,MM;Webster,RG |
| EPI_ISL_305417 | A/Domestic_Duck/Netherlands/EMC-6/2018 | 2018-03-13 | Erasmus Medical Center | Erasmus Medical Center | Poen,MJ;Bestebroer,TM;DeMeulder,D;Vuong,O;Scheuer,RD;NetherlandsFoodandConsumerProductSafetyAuthority,NVWA;Koopmans,MPG;Fouchier,RAM |
| EPI_ISL_304960 | A/duck/Vietnam/QuangBinh/QN530206/2018 | 2018-02-06 | Not listed | Other Database Import | Pham,HM;Pham,HK;Nguyen,VK |
| EPI_ISL_304957 | A/duck/Vietnam/QuangBinh/DH330718/2017 | 2017-07-18 | Not listed | Other Database Import | Pham,HM;Pham,HK;Nguyen,VK |
| EPI_ISL_304956 | A/duck/Vietnam/QuangBinh/DH130723/2017 | 2017-07-23 | Not listed | Other Database Import | Pham,HM;Pham,HK;Nguyen,VK |
| EPI_ISL_303520 | A/Mallard/Republic of Georgia/1/2018 | 2018-01-28 | National Center for Disease Control and Public Health, Georgia | Erasmus Medical Center | Poen,MJ;Bestebroer,TH;Machablishvili,A;Ninua,L;Chkhaidze,M;Vuong,O;Scheuer,RD;Fouchier,RAM;Lewis,NS |
| EPI_ISL_302524 | A/Anas platyrhynchos/Korea/W613/2017 | 2017-12-13 | Not listed | Other Database Import | Kim,YI;Si,YJ;Kwon,HI;Kim,EH;Park,SJ;Robles,NJ;Nguyen,HD;Yu,MA;Yu,KM;Lee,YJ;Lee,MH;Choi,YK;Kim,Y-I;Si,Y-J;Yu,K-M;Choi,Y-K |
| EPI_ISL_302523 | A/Anas platyrhynchos/Korea/W612/2017 | 2017-12-13 | Not listed | Other Database Import | Kim,YI;Si,YJ;Kwon,HI;Kim,EH;Park,SJ;Robles,NJ;Nguyen,HD;Yu,MA;Yu,KM;Lee,YJ;Lee,MH;Choi,YK;Kim,Y-I;Si,Y-J;Yu,K-M;Choi,Y-K |
| EPI_ISL_301784 | A/Mallard/Korea/K17-1825/2017 | 2017-12-22 | Avian diaseases laboratory, College of Veterinary Medicine, Konkuk University | Konkuk University | Kwon,JH;Jeong,S;Kim,YJ;Lee,SH;Song,CS |
| EPI_ISL_300743 | A/mallard/Poland/17/2017 | 2017-01-13 | Not listed | National Veterinary Research Institut Poland, PIWet-PIB | Swieton,E;Smietanka,K |
| EPI_ISL_290253 | A/duck/Vietnam/QuangBinh/LBM0911/2016(H5N6) | 2016-09-11 | Not listed | Other Database Import | Pham,HM;Pham,HK;NguyenVK |
| EPI_ISL_290252 | A/duck/Vietnam/QuangBinh/LBM0909/2016(H5N6) | 2016-09-09 | Not listed | Other Database Import | Pham,HM;Pham,HK;NguyenVK |
| EPI_ISL_284795 | A/duck/Bangladesh/31297/2016 | 2016-11-25 | Not listed | Other Database Import | Barman,S;Turner,JC;Hasan,MK;Akhtar,S;Franks,J;El-Shesheny,R;Walker,D;Seiler,P;Friedman,K;Kercher,L;Kayali,G;Jones-Engel,L;McKenzie,P;Krauss,S;Webby,RJ;Feeroz,MM;Webster,RG |
| EPI_ISL_273394 | A/mallard/Idaho/UGAI16-1964/2016 | 2016-08-18 | Not listed | Other Database Import | Shabman,R;Fedorova,N;Puri,V;Shrivastava,S;Amedeo,P;Isom,R;Hu,L;Durbin,A;Rocchi,I;Williams,T;Poulson,RL;Bao,Y;Sanders,R;Zhdanov,S;Kiryutin,B;Lipman,DJ;Tatusova,T;Stallknecht,DE |
| EPI_ISL_269604 | A/Mallard/Netherlands/1/2017 | 2017-01-07 | Erasmus Medical Center | Erasmus Medical Center | Poen,MJ;VanDerJeugd,HP;Vuong,O;Scheuer,RD;Kleyheeg,E;Bestebroer,TM;Kuiken,T;Fouchier,RAM |
| EPI_ISL_269603 | A/Mallard/Netherlands/51/2016 | 2016-12-20 | Erasmus Medical Center | Erasmus Medical Center | Poen,MJ;VanDerJeugd,HP;Vuong,O;Scheuer,RD;Kleyheeg,E;Bestebroer,TM;Begeman,L;vandenBrand,JMA;Kuiken,T;Fouchier,RAM |
| EPI_ISL_268987 | A/mallard/Czech Republic/2820-17_2/2017 (H5N8) | 2017-02-27 | State Veterinary Institute Prague | State Veterinary Institute Prague | Nagy,A |
| EPI_ISL_268985 | A/mallard/Czech Republic/2678-17_1/2017 (H5N8) | 2017-02-20 | State Veterinary Institute Prague | State Veterinary Institute Prague | Nagy,A |
| EPI_ISL_268983 | A/mallard/Czech Republic/2641-17/2017 (H5N8) | 2017-02-17 | State Veterinary Institute Prague | State Veterinary Institute Prague | Nagy,A |
| EPI_ISL_268969 | A/mallard/Czech Republic/1690-17_2/2017 (H5N8) | 2017-02-05 | State Veterinary Institute Prague | State Veterinary Institute Prague | Nagy,A |
| EPI_ISL_268965 | A/Indian Runner Duck/Czech Republic/1683-17_1/2017 (H5N8) | 2017-02-03 | State Veterinary Institute Prague | State Veterinary Institute Prague | Nagy,A |
| EPI_ISL_268962 | A/mallard/Czech Republic/1672-17/2017 (H5N8) | 2017-01-30 | State Veterinary Institute Prague | State Veterinary Institute Prague | Nagy,A |
| EPI_ISL_268960 | A/mallard/Czech Republic/1577-17/2017 (H5N8) | 2017-01-29 | State Veterinary Institute Prague | State Veterinary Institute Prague | Nagy,A |
| EPI_ISL_268934 | A/mallard/Czech Republic/508-17_4/2017 (H5N8) | 2017-01-12 | State Veterinary Institute Prague | State Veterinary Institute Prague | Nagy,A |
| EPI_ISL_268665 | A/Mal/NL-Mastenbroek/16015378-002/2016 | 2016-12-05 | Wageningen Bioveterinary Research | Wageningen Bioveterinary Research | Beerens,N;Heutink,R;Harders,F;Verschuren-Pritz,S;Bossers,A;Koch,G;Bergervoet,S |
| EPI_ISL_254814 | A/mallard/Hungary/57857/2016 | 2016-11-21 | Central Agricultural Office Veterinary Diagnostic Directorate | Central Agricultural Office Veterinary Diagnostic Directorate | Dan,A |
| EPI_ISL_250886 | A/Indian Runner Duck/Czech Republic/749-17/2017 (H5N8) | 2017-01-16 | Not listed | State Veterinary Institute Prague | Nagy,Alexander |
| EPI_ISL_248663 | A/mallard/Czech Republic/722-17_2/2017 (H5N8) | 2017-01-15 | Not listed | State Veterinary Institute Prague | Nagy,A |
| EPI_ISL_241952 | A/breeder duck/Croatia/21/2017 | 2017-01-14 | Not listed | Croatian Veterinary Institute | Savić,Vladimir |
| EPI_ISL_239420 | A/duck/Hungary/60441/2016 | 2016-12-02 | National Food Chain Safety Office Veterinary Diagnostic Directorate Laboratory for Molecular Biology | Central Agricultural Office Veterinary Diagnostic Directorate | Dan,A |
| EPI_ISL_239262 | A/duck/Korea/ES2/2016(H5N6) | 2016-11-16 | Not listed | Animal and Plant Quarantine Agency (S-2026) | Lee,YJ |
| EPI_ISL_234372 | A/duck/Korea/16A02/2016 | 2016-04-05 | Not listed | Animal and Plant Quarantine Agency (S-2026) | Lee,EK |
| EPI_ISL_234371 | A/breeder duck/Korea/16AQ17/2016 | 2016-03-23 | Not listed | Animal and Plant Quarantine Agency (S-2026) | Lee,EK |
| EPI_ISL_239271 | A/spot_billed_duck/Korea/WB141/2016(H5N6) | 2016-11-10 | Not listed | Animal and Plant Quarantine Agency (S-2026) | Lee,YJ |
| EPI_ISL_273596 | A/American black duck/Alberta/118/2016 | 2016-08-11 | Not listed | Other Database Import | Not listed |
| EPI_ISL_306666 | A/duck/Bangladesh/31992/2017 | 2017-02-15 | Not listed | Other Database Import | Barman,S;Turner,JC;Hasan,MK;Akhtar,S;Franks,J;El-Shesheny,R;Walker,D;Seiler,P;Friedman,K;Kercher,L;Kayali,G;Jones-Engel,L;McKenzie,P;Krauss,S;Webby,RJ;Feeroz,MM;Webster,RG |
| EPI_ISL_306662 | A/duck/Bangladesh/32003/2017 | 2017-02-15 | Not listed | Other Database Import | Barman,S;Turner,JC;Hasan,MK;Akhtar,S;Franks,J;El-Shesheny,R;Walker,D;Seiler,P;Friedman,K;Kercher,L;Kayali,G;Jones-Engel,L;McKenzie,P;Krauss,S;Webby,RJ;Feeroz,MM;Webster,RG |
| EPI_ISL_306657 | A/duck/Bangladesh/31993/2017 | 2017-02-15 | Not listed | Other Database Import | Barman,S;Turner,JC;Hasan,MK;Akhtar,S;Franks,J;El-Shesheny,R;Walker,D;Seiler,P;Friedman,K;Kercher,L;Kayali,G;Jones-Engel,L;McKenzie,P;Krauss,S;Webby,RJ;Feeroz,MM;Webster,RG |
| EPI_ISL_306656 | A/duck/Bangladesh/31998/2017 | 2017-02-15 | Not listed | Other Database Import | Barman,S;Turner,JC;Hasan,MK;Akhtar,S;Franks,J;El-Shesheny,R;Walker,D;Seiler,P;Friedman,K;Kercher,L;Kayali,G;Jones-Engel,L;McKenzie,P;Krauss,S;Webby,RJ;Feeroz,MM;Webster,RG |
| EPI_ISL_284812 | A/duck/Bangladesh/31023/2016 | 2016-10-24 | Not listed | Other Database Import | Barman,S;Turner,JC;Hasan,MK;Akhtar,S;Franks,J;El-Shesheny,R;Walker,D;Seiler,P;Friedman,K;Kercher,L;Kayali,G;Jones-Engel,L;McKenzie,P;Krauss,S;Webby,RJ;Feeroz,MM;Webster,RG |
| EPI_ISL_284808 | A/duck/Bangladesh/30821/2016 | 2016-09-27 | Not listed | Other Database Import | Barman,S;Turner,JC;Hasan,MK;Akhtar,S;Franks,J;El-Shesheny,R;Walker,D;Seiler,P;Friedman,K;Kercher,L;Kayali,G;Jones-Engel,L;McKenzie,P;Krauss,S;Webby,RJ;Feeroz,MM;Webster,RG |
| EPI_ISL_284807 | A/duck/Bangladesh/31095/2016 | 2016-10-24 | Not listed | Other Database Import | Barman,S;Turner,JC;Hasan,MK;Akhtar,S;Franks,J;El-Shesheny,R;Walker,D;Seiler,P;Friedman,K;Kercher,L;Kayali,G;Jones-Engel,L;McKenzie,P;Krauss,S;Webby,RJ;Feeroz,MM;Webster,RG |
| EPI_ISL_284804 | A/duck/Bangladesh/30815/2016 | 2016-09-27 | Not listed | Other Database Import | Barman,S;Turner,JC;Hasan,MK;Akhtar,S;Franks,J;El-Shesheny,R;Walker,D;Seiler,P;Friedman,K;Kercher,L;Kayali,G;Jones-Engel,L;McKenzie,P;Krauss,S;Webby,RJ;Feeroz,MM;Webster,RG |
| EPI_ISL_284790 | A/duck/Bangladesh/31091/2016 | 2016-10-24 | Not listed | Other Database Import | Barman,S;Turner,JC;Hasan,MK;Akhtar,S;Franks,J;El-Shesheny,R;Walker,D;Seiler,P;Friedman,K;Kercher,L;Kayali,G;Jones-Engel,L;McKenzie,P;Krauss,S;Webby,RJ;Feeroz,MM;Webster,RG |
| EPI_ISL_284789 | A/duck/Bangladesh/30057/2016 | 2016-05-26 | Not listed | Other Database Import | Barman,S;Turner,JC;Hasan,MK;Akhtar,S;Franks,J;El-Shesheny,R;Walker,D;Seiler,P;Friedman,K;Kercher,L;Kayali,G;Jones-Engel,L;McKenzie,P;Krauss,S;Webby,RJ;Feeroz,MM;Webster,RG |
| EPI_ISL_284788 | A/duck/Bangladesh/30160/2016 | 2016-05-28 | Not listed | Other Database Import | Barman,S;Turner,JC;Hasan,MK;Akhtar,S;Franks,J;El-Shesheny,R;Walker,D;Seiler,P;Friedman,K;Kercher,L;Kayali,G;Jones-Engel,L;McKenzie,P;Krauss,S;Webby,RJ;Feeroz,MM;Webster,RG |
| EPI_ISL_284787 | A/duck/Bangladesh/30817/2016 | 2016-09-27 | Not listed | Other Database Import | Barman,S;Turner,JC;Hasan,MK;Akhtar,S;Franks,J;El-Shesheny,R;Walker,D;Seiler,P;Friedman,K;Kercher,L;Kayali,G;Jones-Engel,L;McKenzie,P;Krauss,S;Webby,RJ;Feeroz,MM;Webster,RG |
| EPI_ISL_284786 | A/duck/Bangladesh/31028/2016 | 2016-10-24 | Not listed | Other Database Import | Barman,S;Turner,JC;Hasan,MK;Akhtar,S;Franks,J;El-Shesheny,R;Walker,D;Seiler,P;Friedman,K;Kercher,L;Kayali,G;Jones-Engel,L;McKenzie,P;Krauss,S;Webby,RJ;Feeroz,MM;Webster,RG |
| EPI_ISL_284785 | A/duck/Bangladesh/31296/2016 | 2016-11-25 | Not listed | Other Database Import | Barman,S;Turner,JC;Hasan,MK;Akhtar,S;Franks,J;El-Shesheny,R;Walker,D;Seiler,P;Friedman,K;Kercher,L;Kayali,G;Jones-Engel,L;McKenzie,P;Krauss,S;Webby,RJ;Feeroz,MM;Webster,RG |
| EPI_ISL_284777 | A/duck/Bangladesh/30890/2016 | 2016-09-27 | Not listed | Other Database Import | Barman,S;Turner,JC;Hasan,MK;Akhtar,S;Franks,J;El-Shesheny,R;Walker,D;Seiler,P;Friedman,K;Kercher,L;Kayali,G;Jones-Engel,L;McKenzie,P;Krauss,S;Webby,RJ;Feeroz,MM;Webster,RG |
| EPI_ISL_284776 | A/duck/Bangladesh/31294/2016 | 2016-11-25 | Not listed | Other Database Import | Barman,S;Turner,JC;Hasan,MK;Akhtar,S;Franks,J;El-Shesheny,R;Walker,D;Seiler,P;Friedman,K;Kercher,L;Kayali,G;Jones-Engel,L;McKenzie,P;Krauss,S;Webby,RJ;Feeroz,MM;Webster,RG |
| EPI_ISL_284775 | A/duck/Bangladesh/31098/2016 | 2016-10-24 | Not listed | Other Database Import | Barman,S;Turner,JC;Hasan,MK;Akhtar,S;Franks,J;El-Shesheny,R;Walker,D;Seiler,P;Friedman,K;Kercher,L;Kayali,G;Jones-Engel,L;McKenzie,P;Krauss,S;Webby,RJ;Feeroz,MM;Webster,RG |
| EPI_ISL_284774 | A/duck/Bangladesh/31093/2016 | 2016-10-24 | Not listed | Other Database Import | Barman,S;Turner,JC;Hasan,MK;Akhtar,S;Franks,J;El-Shesheny,R;Walker,D;Seiler,P;Friedman,K;Kercher,L;Kayali,G;Jones-Engel,L;McKenzie,P;Krauss,S;Webby,RJ;Feeroz,MM;Webster,RG |
| EPI_ISL_284770 | A/duck/Bangladesh/30885/2016 | 2016-09-27 | Not listed | Other Database Import | Barman,S;Turner,JC;Hasan,MK;Akhtar,S;Franks,J;El-Shesheny,R;Walker,D;Seiler,P;Friedman,K;Kercher,L;Kayali,G;Jones-Engel,L;McKenzie,P;Krauss,S;Webby,RJ;Feeroz,MM;Webster,RG |
| EPI_ISL_284767 | A/duck/Bangladesh/30892/2016 | 2016-09-27 | Not listed | Other Database Import | Barman,S;Turner,JC;Hasan,MK;Akhtar,S;Franks,J;El-Shesheny,R;Walker,D;Seiler,P;Friedman,K;Kercher,L;Kayali,G;Jones-Engel,L;McKenzie,P;Krauss,S;Webby,RJ;Feeroz,MM;Webster,RG |
| EPI_ISL_284714 | A/duck/Bangladesh/31094/2016 | 2016-10-24 | Not listed | Other Database Import | Barman,S;Turner,JC;Hasan,MK;Akhtar,S;Franks,J;El-Shesheny,R;Walker,D;Seiler,P;Friedman,K;Kercher,L;Kayali,G;Jones-Engel,L;McKenzie,P;Krauss,S;Webby,RJ;Feeroz,MM;Webster,RG |
| EPI_ISL_271714 | A/Mulard_duck/Hungary/59163/2016 | 2016-11-24 | National Food Chain Safety Office Veterinary Diagnostic Directorate Laboratory for Molecular Biology | Central Agricultural Office Veterinary Diagnostic Directorate | Dan,A |
| EPI_ISL_271711 | A/Mulard_duck/Hungary/62902/2016 | 2016-12-07 | National Food Chain Safety Office Veterinary Diagnostic Directorate Laboratory for Molecular Biology | Central Agricultural Office Veterinary Diagnostic Directorate | Dan,A |
| EPI_ISL_271710 | A/Mulard_duck/Hungary/60369/2016 | 2016-11-29 | National Food Chain Safety Office Veterinary Diagnostic Directorate Laboratory for Molecular Biology | Central Agricultural Office Veterinary Diagnostic Directorate | Dan,A |
| EPI_ISL_257073 | A/duck/Bangladesh/28389/2016 | 2016-02-29 | Not listed | Other Database Import | Barman,S;Marinova-Petkova,A;Hasan,MK;Akhtar,S;Turner,JC;Franks,J;Walker,D;Seiler,P;Friedman,K;Kercher,L;Kayali,G;Jones-Engel,L;McKenzie,P;Krauss,S;Webby,RJ;Feeroz,MM;Webster,RG |
| EPI_ISL_257052 | A/duck/Bangladesh/28250/2016 | 2016-02-04 | Not listed | Other Database Import | Barman,S;Marinova-Petkova,A;Hasan,MK;Akhtar,S;Turner,JC;Franks,J;Walker,D;Seiler,P;Friedman,K;Kercher,L;Kayali,G;Jones-Engel,L;McKenzie,P;Krauss,S;Webby,RJ;Feeroz,MM;Webster,RG |
| EPI_ISL_294171 | A/gadwall duck/Tennessee/17OS0341/2017 | 2017-01-14 | Not listed | Other Database Import | Killian,M;Franzen,K;Camp,P;Stuber,T;Robbe-Austerman,S;Lauterbach,S;Nolting,J;Bowman,A |
| EPI_ISL_280489 | A/Eurasian wigeon/Shanghai/JDS101/2016 | 2016-11-03 | Not listed | Other Database Import | He,G;Zhou,L;Zhu,C;Shi,H;Li,X;Wu,D;Liu,J;Lv,J;Hu,C;Li,Z;Wang,Z;Wang,T |
| EPI_ISL_250231 | A/gadwall/Chany/97/2016 | 2016-09-10 | Research Institute of Experimental and Clinical Medicine | WHO National Influenza Centre Russian Federation | Sharshov,KA;Sobolev,IA;Li,Xinxin;Fadeev,AV;Egorova,AA;Kotenko,AV;Komissarov,AB;Vasin,AV;Kurskaya,OG;Alexeev,AY;Shestopalov,AM |
| EPI_ISL_266799 | A/eagle/Jakarta Timur/20616-206-III/2016 | 2016-03-07 | Not listed | Other Database Import | Wibawa,H;Poermadjaja,B;Mulyawan,H;Dharmawan,R;Mahawan,T;Hutagaol,NM;Miswati,Y;Srihanto,EA;Hartawan,DHW;Hendrawati,F;Riyadi,A;Deswarni;TriHarsono,A;Hartaningsih,N;Azhar,M;Stegemen,A;McGrane,J;Rasa,FST |
| EPI_ISL_293498 | A/northern goshawk/Mie/2412C007/2016 | 2016-12-14 | Not listed | Other Database Import | Soda,K;Usui,T;Ito,H;Ozaki,H;Yamaguchi,T;Ito,T |
| EPI_ISL_290157 | A/northern goshawk/Tochigi/0912A004/2016 | 2016-12-12 | Not listed | Other Database Import | Soda,K;Usui,T;Ito,H;Ozaki,H;Yamagichi,T;Ito,T;Yamaguchi,T |
| EPI_ISL_309196 | A/Buteo buteo/Belgium/3022/2017 | 2017-03-23 | Not listed | Other Database Import | VanBorm,S;Vandenbussche,F;Mathijs,E;Lambrecht,B;Steensels,M |
| EPI_ISL_268927 | A/Common_Buzzard/Netherlands/1/2016 | 2016-12-07 | Erasmus Medical Center | Erasmus Medical Center | Poen,MJ;Bestebroer,TH;Vuong,O;Scheuer,RD;Fouchier,RAM |
| EPI_ISL_268620 | A/Buzzard/NL-Durgerdam/16015100-004/2016 | 2016-11-30 | Wageningen Bioveterinary Research | Wageningen Bioveterinary Research | Beerens,N;Heutink,R;Harders,F;Verschuren-Pritz,S;Bossers,A;Koch,G;Bergervoet,S |
| EPI_ISL_255208 | A/Common_buzzard/Hungary/7061/2017 | 2017-02-21 | Central Agricultural Office Veterinary Diagnostic Directorate | Central Agricultural Office Veterinary Diagnostic Directorate | Dan,A |
| EPI_ISL_301782 | A/white-tailed_eagle/Denmark/3073-1w/2018-02-13 | 2018-02-13 | Technical Univertity of Denmark | Technical Univertity of Denmark | Hjulsager,C;Krog,J;Larsen,L |
| EPI_ISL_252204 | A/sandpiper/Southcentral Alaska/16MB01259/2016 | 2016-05-09 | Not listed | Other Database Import | Das,SR;Halpin,RA;Lin,X;Simenauer,A;Akopov,A;Fedorova,N;Puri,V;Stockwell,T;Amedeo,P;Katzel,D;Schobel,S;Shrivastava,S;Hill,N;Bao,Y;Sanders,R;Zhdanov,S;Kiryutin,B;Lipman,DJ;Tatusova,T;Runstadler,J |
| EPI_ISL_252196 | A/sandpiper/Southcentral Alaska/16MB01220/2016 | 2016-05-07 | Not listed | Other Database Import | Das,SR;Halpin,RA;Lin,X;Simenauer,A;Akopov,A;Fedorova,N;Puri,V;Stockwell,T;Amedeo,P;Katzel,D;Schobel,S;Shrivastava,S;Hill,N;Bao,Y;Sanders,R;Zhdanov,S;Kiryutin,B;Lipman,DJ;Tatusova,T;Runstadler,J |
| EPI_ISL_252195 | A/sandpiper/Southcentral Alaska/16MB01156/2016 | 2016-05-07 | Not listed | Other Database Import | Das,SR;Halpin,RA;Lin,X;Simenauer,A;Akopov,A;Fedorova,N;Puri,V;Stockwell,T;Amedeo,P;Katzel,D;Schobel,S;Shrivastava,S;Hill,N;Bao,Y;Sanders,R;Zhdanov,S;Kiryutin,B;Lipman,DJ;Tatusova,T;Runstadler,J |
| EPI_ISL_292225 | A/canada_goose/England/AV58_18OPpoolEP1/2018 | 2018-01-05 | Animal and Plant Health Agency (APHA) | Animal and Plant Health Agency (APHA) | Seekings,James;Ellis,Richard;Brookes,SharonM;Reid,Scott;Essen,Stephen;Lewis,Nicola;Brown,IanH |
| EPI_ISL_255938 | A/Rook/Hungary/4975/2017 | 2017-02-08 | National Food Chain Safety Office Veterinary Diagnostic Directorate Laboratory for Molecular Biology | Central Agricultural Office Veterinary Diagnostic Directorate | Dan,A |
| EPI_ISL_288412 | A/Tufted Duck/Netherlands/17017367-007/2017 | 2017-12-09 | Wageningen Bioveterinary Research | Wageningen Bioveterinary Research | Beerens,N;Heutink,R;Harders,F;Verschuren-Pritz,S;Bossers,A;Koch,G;Bergervoet,S |
| EPI_ISL_287564 | A/T_Dk/NL-Werkendam/16014159-001/2016 | 2016-11-14 | Wageningen Bioveterinary Research | Wageningen Bioveterinary Research | Beerens,N;Heutink,R;Harders,F;Verschuren-Pritz,S;Bossers,A;Koch,G;Bergervoet,S |
| EPI_ISL_268681 | A/T_Dk/NL-Zuidoost Beemster/16014148-009/2016 | 2016-11-14 | Wageningen Bioveterinary Research | Wageningen Bioveterinary Research | Beerens,N;Heutink,R;Harders,F;Verschuren-Pritz,S;Bossers,A;Koch,G;Bergervoet,S |
| EPI_ISL_268680 | A/T_Dk/NL-Zuidoost Beemster/16014148-002/2016 | 2016-11-14 | Wageningen Bioveterinary Research | Wageningen Bioveterinary Research | Beerens,N;Heutink,R;Harders,F;Verschuren-Pritz,S;Bossers,A;Koch,G;Bergervoet,S |
| EPI_ISL_268679 | A/T_Dk/NL-Zeewolde/16013976-006/2016 | 2016-11-09 | Wageningen Bioveterinary Research | Wageningen Bioveterinary Research | Beerens,N;Heutink,R;Harders,F;Verschuren-Pritz,S;Bossers,A;Koch,G;Bergervoet,S |
| EPI_ISL_268678 | A/T_Dk/NL-Zeewolde/16013976-005/2016 | 2016-11-09 | Wageningen Bioveterinary Research | Wageningen Bioveterinary Research | Beerens,N;Heutink,R;Harders,F;Verschuren-Pritz,S;Bossers,A;Koch,G;Bergervoet,S |
| EPI_ISL_268676 | A/T_Dk/NL-Zeewolde/16013976-004/2016 | 2016-11-09 | Wageningen Bioveterinary Research | Wageningen Bioveterinary Research | Beerens,N;Heutink,R;Harders,F;Verschuren-Pritz,S;Bossers,A;Koch,G;Bergervoet,S |
| EPI_ISL_268675 | A/T_Dk/NL-Zeewolde/16013976-001-003/2016 | 2016-11-09 | Wageningen Bioveterinary Research | Wageningen Bioveterinary Research | Beerens,N;Heutink,R;Harders,F;Verschuren-Pritz,S;Bossers,A;Koch,G;Bergervoet,S |
| EPI_ISL_268674 | A/T_Dk/NL-Zeewolde/16013976-001/2016 | 2016-11-09 | Wageningen Bioveterinary Research | Wageningen Bioveterinary Research | Beerens,N;Heutink,R;Harders,F;Verschuren-Pritz,S;Bossers,A;Koch,G;Bergervoet,S |
| EPI_ISL_268673 | A/T_Dk/NL-Werkendam/16014159-003/2016 | 2016-11-14 | Wageningen Bioveterinary Research | Wageningen Bioveterinary Research | Beerens,N;Heutink,R;Harders,F;Verschuren-Pritz,S;Bossers,A;Koch,G;Bergervoet,S |
| EPI_ISL_268672 | A/T_Dk/NL-Werkendam/16014159-002/2016 | 2016-11-14 | Wageningen Bioveterinary Research | Wageningen Bioveterinary Research | Beerens,N;Heutink,R;Harders,F;Verschuren-Pritz,S;Bossers,A;Koch,G;Bergervoet,S |
| EPI_ISL_268670 | A/T_Dk/NL-Roggebotsluis/16014462-015/2016 | 2016-11-17 | Wageningen Bioveterinary Research | Wageningen Bioveterinary Research | Beerens,N;Heutink,R;Harders,F;Verschuren-Pritz,S;Bossers,A;Koch,G;Bergervoet,S |
| EPI_ISL_268668 | A/T_Dk/NL-Almeerder Zand/16014341-003/2016 | 2016-11-16 | Wageningen Bioveterinary Research | Wageningen Bioveterinary Research | Beerens,N;Heutink,R;Harders,F;Verschuren-Pritz,S;Bossers,A;Koch,G;Bergervoet,S |
| EPI_ISL_257699 | A/Tufted Duck/Switzerland/V237/2016 | 2016-11-13 | Institute of Virology and Immunology IVI | Faculty of Veterinary Medicine at the University of Bern | Dijkman,R |
| EPI_ISL_255891 | A/Tufted Duck/Netherlands/1/2016 | 2016-11-25 | Erasmus Medical Center | Erasmus Medical Center | Poen,MJ;VanDerJeugd,HP;Vuong,O;Scheuer,RD;Kleyheeg,E;Lexmond,P;Eggink,WD;Müskens,GJDM;Bestebroer,TM;Koopmans,MPG;Kuiken,T;Fouchier,RAM |
| EPI_ISL_247713 | A/tufted duck/Denmark/17740-1/2016 | 2016-11-08 | Technical University of Denmark | Animal and Plant Health Agency (APHA) | Hjulsager,CK;Krog,JS;Larsen,LE;Kvisgaard,LK;Essen,S |
| EPI_ISL_238040 | A/tufted duck/Germany-SN/R8795/2016 | 2016-11-11 | Not listed | Friedrich-Loeffler-Institut | Starick,E |
| EPI_ISL_237945 | A/tufted_duck/Germany/AR8459-L01988/2016 | 2016-11-08 | Not listed | Friedrich-Loeffler-Institut | Pohlmann,A |
| EPI_ISL_237733 | A/tufted_duck/Germany/AR8444-L01986/2016 | 2016-11-07 | Not listed | Friedrich-Loeffler-Institut | Pohlmann,A |
| EPI_ISL_237732 | A/tufted_duck/Germany-SH/R8446/2016 | 2016-11-07 | Not listed | Friedrich-Loeffler-Institut | Pohlmann,A |
| EPI_ISL_290312 | A/greater scaup/Aichi/2301H050/2017 | 2017-01-31 | Not listed | Other Database Import | Soda,K;Usui,T;Ito,H;Ozaki,H;Yamaguchi,T;Ito,T |
| EPI_ISL_309580 | A/ruddy turnstone/New Jersey/UGAI17-1985/2017 | 2017-05-13 | Not listed | Other Database Import | Tan,G;Pickett,B;Fedorova,N;Amedeo,P;Isom,R;Hu,L;Christensen,J;Durbin,A;Williams,T;Arumemi,F;Poulson,R;Bao,Y;Sanders,R;Zhdanov,S;Kiryutin,B;Lipman,DJ;Tatusova,T;Hatcher,E;Stallknecht,D |
| EPI_ISL_295820 | A/muscovy duck/Vietnam/HU7-23/2017 | 2017-08-01 | Not listed | Other Database Import | Nguyen;LT;Chu;DH;Matsuno,K;Okamatsu,M;Sakoda,Y;Jizou,M |
| EPI_ISL_295819 | A/muscovy duck/Vietnam/HU7-20/2017 | 2017-08-01 | Not listed | Other Database Import | Nguyen;LT;Chu;DH;Matsuno,K;Okamatsu,M;Sakoda,Y;Jizou,M |
| EPI_ISL_295818 | A/muscovy duck/Vietnam/HU7-17/2017 | 2017-08-01 | Not listed | Other Database Import | Nguyen;LT;Chu;DH;Matsuno,K;Okamatsu,M;Sakoda,Y;Jizou,M |
| EPI_ISL_295790 | A/muscovy duck/Vietnam/HU8-1670/2017 | 2017-08-21 | Not listed | Other Database Import | Nguyen,LT;Chu,DH;Okamatsu,M;Matsuno,K;Sakoda,Y;Jizou,M |
| EPI_ISL_295753 | A/muscovy duck/Vietnam/HU8-1569/2017 | 2017-08-21 | Not listed | Other Database Import | Nguyen,LT;Chu,DH;Okamatsu,M;Matsuno,K;Sakoda,Y;Jizou,M |
| EPI_ISL_266797 | A/muscovy duck/Pekalongan/04160460/2016 | 2016-04-04 | Not listed | Other Database Import | Wibawa,H;Poermadjaja,B;Mulyawan,H;Dharmawan,R;Mahawan,T;Hutagaol,NM;Miswati,Y;Srihanto,EA;Hartawan,DHW;Hendrawati,F;Riyadi,A;Deswarni;TriHarsono,A;Hartaningsih,N;Azhar,M;Stegemen,A;McGrane,J;Rasa,FST |
| EPI_ISL_238897 | A/Common Goldeneye/Sweden/SVA161117KU0322/SZ0002165/2016 | 2016-11-14 | National Veterinary Institute | National Veterinary Institute | Zohari,S |
| EPI_ISL_303643 | A/Mandarin_duck/Korea/K18-3/2018 | 2018-01-18 | Avian diaseases laboratory, College of Veterinary Medicine, Konkuk University | Konkuk University | Kwon,JH;Jeong,S;Kim,YJ;Lee,SH;Song,CS |
| EPI_ISL_303642 | A/Mandarin_duck/Korea/K17-1896/2017 | 2017-12-23 | Avian diaseases laboratory, College of Veterinary Medicine, Konkuk University | Konkuk University | Kwon,JH;Jeong,S;Kim,YJ;Lee,SH;Song,CS |
| EPI_ISL_303641 | A/Mandarin_duck/Korea/K17-1894/2017 | 2017-12-23 | Avian diaseases laboratory, College of Veterinary Medicine, Konkuk University | Konkuk University | Kwon,JH;Jeong,S;Kim,YJ;Lee,SH;Song,CS |
| EPI_ISL_303640 | A/Mandarin_duck/Korea/K17-1893/2017 | 2017-12-23 | Avian diaseases laboratory, College of Veterinary Medicine, Konkuk University | Konkuk University | Kwon,JH;Jeong,S;Kim,YJ;Lee,SH;Song,CS |
| EPI_ISL_303639 | A/Mandarin_duck/Korea/K17-1891/2017 | 2017-12-23 | Avian diaseases laboratory, College of Veterinary Medicine, Konkuk University | Konkuk University | Kwon,JH;Jeong,S;Kim,YJ;Lee,SH;Song,CS |
| EPI_ISL_303638 | A/Mandarin_duck/Korea/K17-1889/2017 | 2017-12-23 | Avian diaseases laboratory, College of Veterinary Medicine, Konkuk University | Konkuk University | Kwon,JH;Jeong,S;Kim,YJ;Lee,SH;Song,CS |
| EPI_ISL_303637 | A/Mandarin_duck/Korea/K17-1887/2017 | 2017-12-23 | Avian diaseases laboratory, College of Veterinary Medicine, Konkuk University | Konkuk University | Kwon,JH;Jeong,S;Kim,YJ;Lee,SH;Song,CS |
| EPI_ISL_303635 | A/Mandarin_duck/Korea/K17-1881/2017 | 2017-12-23 | Avian diaseases laboratory, College of Veterinary Medicine, Konkuk University | Konkuk University | Kwon,JH;Jeong,S;Kim,YJ;Lee,SH;Song,CS |
| EPI_ISL_303634 | A/Mandarin_duck/Korea/K17-1879/2017 | 2017-12-23 | Avian diaseases laboratory, College of Veterinary Medicine, Konkuk University | Konkuk University | Kwon,JH;Jeong,S;Kim,YJ;Lee,SH;Song,CS |
| EPI_ISL_303633 | A/Mandarin_duck/Korea/K17-1873/2017 | 2017-12-23 | Avian diaseases laboratory, College of Veterinary Medicine, Konkuk University | Konkuk University | Kwon,JH;Jeong,S;Kim,YJ;Lee,SH;Song,CS |
| EPI_ISL_303632 | A/Mandarin_duck/Korea/K17-1869/2017 | 2017-12-23 | Avian diaseases laboratory, College of Veterinary Medicine, Konkuk University | Konkuk University | Kwon,JH;Jeong,S;Kim,YJ;Lee,SH;Song,CS |
| EPI_ISL_303631 | A/Mandarin_duck/Korea/K17-1866/2017 | 2017-12-23 | Avian diaseases laboratory, College of Veterinary Medicine, Konkuk University | Konkuk University | Kwon,JH;Jeong,S;Kim,YJ;Lee,SH;Song,CS |
| EPI_ISL_303630 | A/Mandarin_duck/Korea/K17-1862/2017 | 2017-12-23 | Avian diaseases laboratory, College of Veterinary Medicine, Konkuk University | Konkuk University | Kwon,JH;Jeong,S;Kim,YJ;Lee,SH;Song,CS |
| EPI_ISL_301803 | A/Mandarin_duck/Korea/K17-1828/2017 | 2017-12-22 | Avian diaseases laboratory, College of Veterinary Medicine, Konkuk University | Konkuk University | Kwon,JH;Jeong,S;Kim,YJ;Lee,SH;Song,CS |
| EPI_ISL_301786 | A/Mandarin_duck/Korea/K17-1817/2017 | 2017-12-22 | Avian diaseases laboratory, College of Veterinary Medicine, Konkuk University | Konkuk University | Kwon,JH;Jeong,S;Kim,YJ;Lee,SH;Song,CS |
| EPI_ISL_290073 | A/mandarin duck/Tochigi/0902C033/2017 | 2017-02-20 | Not listed | Other Database Import | Soda,K;Usui,T;Ito,H;Ozaki,H;Yamaguchi,T;Ito,T |
| EPI_ISL_280490 | A/mandarin duck/Shanghai/PD4/2016 | 2016-11-17 | Not listed | Other Database Import | He,G;Zhou,L;Zhu,C;Shi,H;Li,X;Wu,D;Liu,J;Lv,J;Hu,C;Li,Z;Wang,Z;Wang,T |
| EPI_ISL_238148 | A/Mandarin_duck/Korea/K16-187-3/2016 | 2016-10-28 | Avian diaseases laboratory, College of Veterinary Medicine, Konkuk University | Konkuk University | Jung-Hoon,Kwon;Jin-Yong,Noh;Seong-Su,Yuk;Tseren-Ochir,Erdene-Ochir;Woo-Tack,Hong;Jei-Hyun,Jeong;Sol,Jeong;Gyeong-Bin,Gwon;Jiho,Lee;Yujin,Kim;Kyujik,Kim;Junbeom,Kim;Chang-Seon,Song |
| EPI_ISL_289714 | A/Black-headed Gull/Netherlands/29/2017 | 2017-12-18 | Erasmus Medical Center | Erasmus Medical Center | Poen,MJ;Bestebroer,TM;Kelder,L;Scheuer,RD;Koopmans,MPG;VanDerJeugd,HP;Fouchier,RAM |
| EPI_ISL_268800 | A/Black-headed_Gull/Netherlands/17/2016 | 2016-12-20 | Erasmus Medical Center | Erasmus Medical Center | Poen,MJ;VanDerJeugd,HP;Vuong,O;Scheuer,RD;Kleyheeg,E;Bestebroer,TM;Begeman,L;vandenBrand,JMA;Kuiken,T;Fouchier,RAM |
| EPI_ISL_268799 | A/Back-headed_Gull/Netherlands/8/2016 | 2016-12-20 | Erasmus Medical Center | Erasmus Medical Center | Poen,MJ;VanDerJeugd,HP;Vuong,O;Scheuer,RD;Kleyheeg,E;Bestebroer,TM;Begeman,L;vandenBrand,JMA;Kuiken,T;Fouchier,RAM |
| EPI_ISL_243050 | A/barnacle goose/Germany-SH/R11505/2016 | 2016-12-22 | Not listed | Friedrich-Loeffler-Institut | Starick,E |
| EPI_ISL_282408 | A/poultry/China/XY918.4/2016 | 2016-09 | Not listed | Other Database Import | Zhao,Z |
| EPI_ISL_302826 | A/Chicken/Netherlands/EMC-14/2018 | 2018-02-26 | Erasmus Medical Center | Erasmus Medical Center | Poen,MJ;Bestebroer,TM;DeMeulder,D;Vuong,O;Scheuer,RD;NetherlandsFoodandConsumerProductSafetyAuthority,NVWA;Koopmans,MPG;Fouchier,RAM |
| EPI_ISL_297466 | A/chicken/Kostroma/1721/2017 | 2017-12-22 | Not listed | State Research Center of Virology and Biotechnology (VECTOR) | Susloparov,I;Goncharova,N;Kolosova,N;Marchenko,V;Ryzhikov,A |
| EPI_ISL_297465 | A/chicken/Kostroma/1720/2017 | 2017-12-22 | Not listed | State Research Center of Virology and Biotechnology (VECTOR) | Susloparov,I;Goncharova,N;Kolosova,N;Marchenko,V;Ryzhikov,A |
| EPI_ISL_297464 | A/chicken/Kostroma/1719/2017 | 2017-12-22 | Not listed | State Research Center of Virology and Biotechnology (VECTOR) | Susloparov,I;Goncharova,N;Kolosova,N;Marchenko,V;Ryzhikov,A |
| EPI_ISL_297463 | A/chicken/Kostroma/1717/2017 | 2017-12-22 | Not listed | State Research Center of Virology and Biotechnology (VECTOR) | Susloparov,I;Goncharova,N;Kolosova,N;Marchenko,V;Ryzhikov,A |
| EPI_ISL_297235 | A/chicken/Rostov-on-Don/1598/2017 | 2017-11-17 | Not listed | State Research Center of Virology and Biotechnology (VECTOR) | Susloparov,I;Goncharova,N;Kolosova,N;Marchenko,V;Ryzhikov,A |
| EPI_ISL_295027 | A/chicken/Kostroma/1718/2017 | 2017-12-22 | Not listed | State Research Center of Virology and Biotechnology (VECTOR) | Susloparov,I;Goncharova,N;Kolosova,N;Marchenko,V;Ryzhikov,A |
| EPI_ISL_293483 | A/chicken/Kumamoto/45/2016 | 2016-12-26 | Not listed | Other Database Import | Soda,K;Usui,T;Ito,H;Ozaki,H;Yamaguchi,T;Ito,T |
| EPI_ISL_288364 | A/chicken/Greece/39_2017b/2017 | 2017-02-06 | Thessalonica Veterinary Centre (TVC) | Animal and Plant Health Agency (APHA) | Seekings,James;Ellis,Richard;Brookes,SharonM;Essen,Stephen;Lewis,Nicola;Brown,IanH;Dovas,C;Georgiades,D |
| EPI_ISL_288363 | A/chicken/Greece/39_2017a/2017 | 2017-02-06 | Thessalonica Veterinary Centre (TVC) | Animal and Plant Health Agency (APHA) | Seekings,James;Ellis,Richard;Brookes,SharonM;Essen,Stephen;Lewis,Nicola;Brown,IanH;Dovas,C;Georgiades,D |
| EPI_ISL_288362 | A/chicken/Greece/39_2017/2017 | 2017-02-06 | Thessalonica Veterinary Centre (TVC) | Animal and Plant Health Agency (APHA) | Seekings,James;Ellis,Richard;Brookes,SharonM;Reid,Stephen;Lewis,Nicola;Brown,IanH;Dovas,C;Georgiades,D |
| EPI_ISL_285917 | A/Chicken/South_Africa/S2017/09_0184_62/2017 | 2017-09-08 | Western Cape Provincial Veterinary Laboratory | National Institute of Communicable Diseases | Treurnicht,FK |
| EPI_ISL_285915 | A/Chicken/South_Africa/S2017/08_0581_P1/2017 | 2017-08-30 | Western Cape Provincial Veterinary Laboratory | National Institute of Communicable Diseases | Treurnicht,FK |
| EPI_ISL_285658 | A/Chicken/South_Africa/S2017/08_0561_P1/2017 | 2017-08-29 | Western Cape Provincial Veterinary Laboratory | National Institute of Communicable Diseases | Treurnicht,FK |
| EPI_ISL_284004 | A/Chicken/South_Africa/S2017/08_0336_P3/2017 | 2017-08-21 | Western Cape Provincial Veterinary Laboratory | National Institute of Communicable Diseases | Treurnicht,FK |
| EPI_ISL_309634 | A/ruddy turnstone/New Jersey/UGAI17-2989/2017 | 2017-05-27 | Not listed | Other Database Import | Tan,G;Pickett,B;Fedorova,N;Amedeo,P;Isom,R;Hu,L;Christensen,J;Durbin,A;Williams,T;Arumemi,F;Poulson,R;Bao,Y;Sanders,R;Zhdanov,S;Kiryutin,B;Lipman,DJ;Tatusova,T;Hatcher,E;Stallknecht,D |
| EPI_ISL_309632 | A/ruddy turnstone/New Jersey/UGAI17-2975/2017 | 2017-05-27 | Not listed | Other Database Import | Tan,G;Pickett,B;Fedorova,N;Amedeo,P;Isom,R;Hu,L;Christensen,J;Durbin,A;Williams,T;Arumemi,F;Poulson,R;Bao,Y;Sanders,R;Zhdanov,S;Kiryutin,B;Lipman,DJ;Tatusova,T;Hatcher,E;Stallknecht,D |
| EPI_ISL_309611 | A/ruddy turnstone/New Jersey/UGAI17-2770/2017 | 2017-05-22 | Not listed | Other Database Import | Tan,G;Pickett,B;Fedorova,N;Amedeo,P;Isom,R;Hu,L;Christensen,J;Durbin,A;Williams,T;Arumemi,F;Poulson,R;Bao,Y;Sanders,R;Zhdanov,S;Kiryutin,B;Lipman,DJ;Tatusova,T;Hatcher,E;Stallknecht,D |
| EPI_ISL_309591 | A/ruddy turnstone/New Jersey/UGAI17-2746/2017 | 2017-05-19 | Not listed | Other Database Import | Tan,G;Pickett,B;Fedorova,N;Amedeo,P;Isom,R;Hu,L;Christensen,J;Durbin,A;Williams,T;Arumemi,F;Poulson,R;Bao,Y;Sanders,R;Zhdanov,S;Kiryutin,B;Lipman,DJ;Tatusova,T;Hatcher,E;Stallknecht,D |
| EPI_ISL_309588 | A/ruddy turnstone/New Jersey/UGAI17-2667/2017 | 2017-05-19 | Not listed | Other Database Import | Tan,G;Pickett,B;Fedorova,N;Amedeo,P;Isom,R;Hu,L;Christensen,J;Durbin,A;Williams,T;Arumemi,F;Poulson,R;Bao,Y;Sanders,R;Zhdanov,S;Kiryutin,B;Lipman,DJ;Tatusova,T;Hatcher,E;Stallknecht,D |
| EPI_ISL_309583 | A/ruddy turnstone/New Jersey/UGAI17-2003/2017 | 2017-05-13 | Not listed | Other Database Import | Tan,G;Pickett,B;Fedorova,N;Amedeo,P;Isom,R;Hu,L;Christensen,J;Durbin,A;Williams,T;Arumemi,F;Poulson,R;Bao,Y;Sanders,R;Zhdanov,S;Kiryutin,B;Lipman,DJ;Tatusova,T;Hatcher,E;Stallknecht,D |
| EPI_ISL_307653 | A/ruddy turnstone/New Jersey/UGAI17-2652/2017 | 2017-05-19 | Not listed | Other Database Import | Tan,G;Pickett,B;Fedorova,N;Amedeo,P;Isom,R;Hu,L;Christensen,J;Durbin,A;Williams,T;Arumemi,F;Poulson,R;Bao,Y;Sanders,R;Zhdanov,S;Kiryutin,B;Lipman,DJ;Tatusova,T;Hatcher,E;Stallknecht,D |
| EPI_ISL_306672 | A/ruddy turnstone/Delaware Bay/327/2017 | 2017-05-21 | Not listed | Other Database Import | Not listed |
| EPI_ISL_303851 | A/ruddy turnstone/Delaware Bay/272/2017 | 2017-05-21 | Not listed | Other Database Import | Not listed |
| EPI_ISL_273583 | A/ruddy turnstone/Delaware Bay/317/2016 | 2016-05-24 | Not listed | Other Database Import | Not listed |
| EPI_ISL_252211 | A/least sandpiper/Southcentral Alaska/16MB01324/2016 | 2016-05-11 | Not listed | Other Database Import | Das,SR;Halpin,RA;Lin,X;Simenauer,A;Akopov,A;Fedorova,N;Puri,V;Stockwell,T;Amedeo,P;Katzel,D;Schobel,S;Shrivastava,S;Hill,N;Bao,Y;Sanders,R;Zhdanov,S;Kiryutin,B;Lipman,DJ;Tatusova,T;Runstadler,J |
| EPI_ISL_252210 | A/least sandpiper/Southcentral Alaska/16MB01323/2016 | 2016-05-11 | Not listed | Other Database Import | Das,SR;Halpin,RA;Lin,X;Simenauer,A;Akopov,A;Fedorova,N;Puri,V;Stockwell,T;Amedeo,P;Katzel,D;Schobel,S;Shrivastava,S;Hill,N;Bao,Y;Sanders,R;Zhdanov,S;Kiryutin,B;Lipman,DJ;Tatusova,T;Runstadler,J |
| EPI_ISL_293503 | A/common porchard/Yamaguchi/3501B002/2017 | 2017-01-09 | Not listed | Other Database Import | Soda,K;Usui,T;Ito,H;Ozaki,H;Yamaguchi,T;Ito,T |
| EPI_ISL_291109 | A/common_pochard/Germany-BY/AR09-18-L02421/2017 | 2017-12-28 | Not listed | Friedrich-Loeffler-Institut | Pohlmann,A |
| EPI_ISL_290071 | A/common porchard/Aichi/2301N025/2017 | 2017-01-11 | Not listed | Other Database Import | Soda,K;Usui,T;Ito,H;Ozaki,H;Yamaguchi,T;Ito,T |
| EPI_ISL_269693 | A/Common_Pochard/Netherlands/1/2016 | 2016-11-25 | Erasmus Medical Center | Erasmus Medical Center | Poen,MJ;VanDerJeugd,HP;Vuong,O;Scheuer,RD;Kleyheeg,E;Bestebroer,TM;Kuiken,T;Fouchier,RAM |
| EPI_ISL_255191 | A/Harris Hawk/Hungary/120/2017 | 2017-01-02 | Central Agricultural Office Veterinary Diagnostic Directorate | Central Agricultural Office Veterinary Diagnostic Directorate | Dan,A |
| EPI_ISL_255210 | A/Harris_hawk/Hungary/2762a/2017 | 2017-01-24 | Central Agricultural Office Veterinary Diagnostic Directorate | Central Agricultural Office Veterinary Diagnostic Directorate | Dan,A |
| EPI_ISL_268666 | A/P_falcon/NL-Vrouwenpolder (Zeeland)/16015510-001/2016 | 2016-12-07 | Wageningen Bioveterinary Research | Wageningen Bioveterinary Research | Beerens,N;Heutink,R;Harders,F;Verschuren-Pritz,S;Bossers,A;Koch,G;Bergervoet,S |
| EPI_ISL_260782 | A/peregrine falcon/Hokkaido/X2/2016 | 2016-11-24 | Not listed | Hokkaido University | MasatoshiOkamatsu |
| EPI_ISL_298653 | A/peregrine falcon/Israel/1086/2016 | 2016-12-25 | Not listed | Other Database Import | Shkoda,I;Lapin,K;Simanov,L;Lublin,A |
| EPI_ISL_293499 | A/peregrine falcon/Nagasaki/4212A005/2016 | 2016-12-22 | Not listed | Other Database Import | Soda,K;Usui,T;Ito,H;Ozaki,H;Yamaguchi,T;Ito,T |
| EPI_ISL_290310 | A/peregrine falcon/Niigata/15/2016 | 2016-12-01 | Not listed | Other Database Import | Soda,K;Usui,T;Ito,H;Ozaki,H;Yamaguchi,T;Ito,T |
| EPI_ISL_256496 | A/peregrine falcon/Hokkaido/X7/2016 | 2016-12-13 | Graduate School of Veterinary Medicine, Hokkaido University | Hokkaido University | MasatoshiOkamatsu |
| EPI_ISL_301039 | A/domestic_goose/Poland/190/2017 | 2017-02-19 | Not listed | National Veterinary Research Institut Poland, PIWet-PIB | Swieton,E;Smietanka,K |
| EPI_ISL_300703 | A/domestic_goose/Poland/124/2017 | 2017-02-06 | National Veterinary Research Institut Poland, PIWet-PIB | National Veterinary Research Institut Poland, PIWet-PIB | Swieton,E;Smietanka,K |
| EPI_ISL_284692 | A/Geese/South_Africa/S2017/09_0055_P2/2017 | 2017-09-04 | Western Cape Provincial Veterinary Laboratory | National Institute of Communicable Diseases | Treurnicht,FK |
| EPI_ISL_271707 | A/Goose/Hungary/17580/2017 | 2017-04-19 | National Food Chain Safety Office Veterinary Diagnostic Directorate Laboratory for Molecular Biology | Central Agricultural Office Veterinary Diagnostic Directorate | Dan,A |
| EPI_ISL_271706 | A/Goose/Hungary/17051/2017 | 2017-04-18 | National Food Chain Safety Office Veterinary Diagnostic Directorate Laboratory for Molecular Biology | Central Agricultural Office Veterinary Diagnostic Directorate | Dan,A |
| EPI_ISL_271705 | A/Goose/Hungary/15729/2017 | 2017-04-07 | National Food Chain Safety Office Veterinary Diagnostic Directorate Laboratory for Molecular Biology | Central Agricultural Office Veterinary Diagnostic Directorate | Dan,A |
| EPI_ISL_271704 | A/Goose/Hungary/17261/2017 | 2017-04-18 | National Food Chain Safety Office Veterinary Diagnostic Directorate Laboratory for Molecular Biology | Central Agricultural Office Veterinary Diagnostic Directorate | Dan,A |
| EPI_ISL_240103 | A/domestic goose/Poland/72/2016 | 2016-12-14 | Not listed | National Veterinary Research Institut Poland, PIWet-PIB | Swieton,E;Smietanka,K |
| EPI_ISL_240102 | A/domestic goose/Poland/33/2016 | 2016-12-02 | Not listed | National Veterinary Research Institut Poland, PIWet-PIB | Swieton,E;Smietanka,K |
| EPI_ISL_256515 | A/white-fronted goose/Miyagi/2/2016 | 2016-11-26 | Not listed | Hokkaido University | MasatoshiOkamatsu |
| EPI_ISL_255219 | A/White_fronted_goose/Hungary/801/2017 | 2017-01-09 | Central Agricultural Office Veterinary Diagnostic Directorate | Central Agricultural Office Veterinary Diagnostic Directorate | Dan,A |
| EPI_ISL_298652 | A/grey goose/Israel/986/2016 | 2016-12-12 | Not listed | Other Database Import | Shkoda,I;Lapin,K;Simanov,L;Lublin,A |
| EPI_ISL_268930 | A/goose/Czech Republic/136-17_1/2017 (H5N8) | 2017-01-04 | State Veterinary Institute Prague | State Veterinary Institute Prague | Nagy,A |
| EPI_ISL_268657 | A/Grey_Go/NL-Groot-Ammers/16015901-012/2016 | 2016-12-14 | Wageningen Bioveterinary Research | Wageningen Bioveterinary Research | Beerens,N;Heutink,R;Harders,F;Verschuren-Pritz,S;Bossers,A;Koch,G;Bergervoet,S |
| EPI_ISL_256309 | A/goose/Czech Republic/821-17_2/2017 (H5N8) | 2017-01-17 | Not listed | State Veterinary Institute Prague | Nagy,A |
| EPI_ISL_237965 | A/goose/Hungary/55128/2016 | 2016-11-11 | National Food Chain Safety Office Veterinary Diagnostic Directorate Laboratory for Molecular Biology | Central Agricultural Office Veterinary Diagnostic Directorate | Ronai,Z;Ursu,K;Balint,A;Szalay,A;Thuma,A;Gyuris,E;Erdelyi,K;Dan,A |
| EPI_ISL_224737 | A/Bar-headed Goose/Qinghai/BTY17-B/2016 | 2016-05-15 | State Key Laboratory of Virology and Wuhan Institute of Virology, Chinese Academy of Sciences | Wuhan Institute of Virology | Chen,J |
| EPI_ISL_224735 | A/Bar-headed Goose/Qinghai/BTY16-B/2016 | 2016-05-14 | State Key Laboratory of Virology and Wuhan Institute of Virology, Chinese Academy of Sciences | Wuhan Institute of Virology | Chen,J |
| EPI_ISL_224733 | A/Bar-headed Goose/Qinghai/BTY15-B/2016 | 2016-05-14 | State Key Laboratory of Virology and Wuhan Institute of Virology, Chinese Academy of Sciences | Wuhan Institute of Virology | Chen,J |
| EPI_ISL_224732 | A/Bar-headed Goose/Qinghai/BTY14-LU/2016 | 2016-05-14 | State Key Laboratory of Virology and Wuhan Institute of Virology, Chinese Academy of Sciences | Wuhan Institute of Virology | Chen,J |
| EPI_ISL_224730 | A/Bar-headed Goose/Qinghai/BTY13-LU/2016 | 2016-05-14 | State Key Laboratory of Virology and Wuhan Institute of Virology, Chinese Academy of Sciences | Wuhan Institute of Virology | Chen,J |
| EPI_ISL_224727 | A/Bar-headed Goose/Qinghai/BTY12-B/2016 | 2016-05-13 | State Key Laboratory of Virology and Wuhan Institute of Virology, Chinese Academy of Sciences | Wuhan Institute of Virology | Chen,J |
| EPI_ISL_224723 | A/Bar-headed Goose/Qinghai/BTY10-B/2016 | 2016-05-09 | State Key Laboratory of Virology and Wuhan Institute of Virology, Chinese Academy of Sciences | Wuhan Institute of Virology | Chen,J |
| EPI_ISL_224722 | A/Bar-headed Goose/Qinghai/BTY9-LU/2016 | 2016-05-09 | State Key Laboratory of Virology and Wuhan Institute of Virology, Chinese Academy of Sciences | Wuhan Institute of Virology | Chen,J |
| EPI_ISL_224717 | A/Bar-headed Goose/Qinghai/BTY7-LU1/2016 | 2016-05-11 | State Key Laboratory of Virology and Wuhan Institute of Virology, Chinese Academy of Sciences | Wuhan Institute of Virology | Chen,J |
| EPI_ISL_224716 | A/Bar-headed Goose/Qinghai/BTY7-B/2016 | 2016-05-11 | State Key Laboratory of Virology and Wuhan Institute of Virology, Chinese Academy of Sciences | Wuhan Institute of Virology | Chen,J |
| EPI_ISL_224715 | A/Bar-headed Goose/Qinghai/BTY6-LU/2016 | 2016-05-11 | State Key Laboratory of Virology and Wuhan Institute of Virology, Chinese Academy of Sciences | Wuhan Institute of Virology | Chen,J |
| EPI_ISL_224714 | A/Bar-headed Goose/Qinghai/BTY6-B/2016 | 2016-05-11 | State Key Laboratory of Virology and Wuhan Institute of Virology, Chinese Academy of Sciences | Wuhan Institute of Virology | Chen,J |
| EPI_ISL_224705 | A/Bar-headed Goose/Qinghai/BTY1-LV/2016 | 2016-05-09 | State Key Laboratory of Virology and Wuhan Institute of Virology, Chinese Academy of Sciences | Wuhan Institute of Virology | Chen,J |
| EPI_ISL_278027 | A/Goose/Qingyuan/16875/2016 | 2016-08-29 | South China Agricultural University | South China Agricultural University | Weixin,J |
| EPI_ISL_285651 | A/Guineafowl/South_Africa/S2017/08_0190_9/2017 | 2017-08-14 | Western Cape Provincial Veterinary Laboratory | National Institute of Communicable Diseases | Treurnicht,FK |
| EPI_ISL_268660 | A/Gull10/NL-Marker Wadden/16014466-014/2016 | 2016-11-17 | Wageningen Bioveterinary Research | Wageningen Bioveterinary Research | Beerens,N;Heutink,R;Harders,F;Verschuren-Pritz,S;Bossers,A;Koch,G;Bergervoet,S |
| EPI_ISL_247406 | A/black headed gull/Ibaraki/194T/2016 | 2016-12-06 | National Institute of Animal Health | National Institute of Animal Health | Takehiko,S |
| EPI_ISL_240111 | A/herring gull/Poland/84/2016 | 2016-11-09 | Not listed | National Veterinary Research Institut Poland, PIWet-PIB | Swieton,E;Smietanka,K |
| EPI_ISL_224750 | A/Brown-headed Gull/Qinghai/ZTO6-SP/2016 | 2016-05-15 | State Key Laboratory of Virology and Wuhan Institute of Virology, Chinese Academy of Sciences | Wuhan Institute of Virology | Chen,J |
| EPI_ISL_224744 | A/Brown-headed Gull/Qinghai/ZTO3-B/2016 | 2016-05-12 | State Key Laboratory of Virology and Wuhan Institute of Virology, Chinese Academy of Sciences | Wuhan Institute of Virology | Chen,J |
| EPI_ISL_224743 | A/Brown-headed Gull/Qinghai/ZTO1-LU/2016 | 2016-05-01 | State Key Laboratory of Virology and Wuhan Institute of Virology, Chinese Academy of Sciences | Wuhan Institute of Virology | Chen,J |
| EPI_ISL_224742 | A/Brown-headed Gull/Qinghai/ZTO1-B/2016 | 2016-05-01 | State Key Laboratory of Virology and Wuhan Institute of Virology, Chinese Academy of Sciences | Wuhan Institute of Virology | Chen,J |
| EPI_ISL_268916 | A/Caspian _Gull/Netherlands/1/2016 | 2016-12-20 | Erasmus Medical Center | Erasmus Medical Center | Poen,MJ;VanDerJeugd,HP;Vuong,O;Scheuer,RD;Kleyheeg,E;Bestebroer,TM;Begeman,L;vandenBrand,JMA;Kuiken,T;Fouchier,RAM |
| EPI_ISL_268621 | A/C_Gull/NL-Slootdorp/16014102-003/2016 | 2016-11-11 | Wageningen Bioveterinary Research | Wageningen Bioveterinary Research | Beerens,N;Heutink,R;Harders,F;Verschuren-Pritz,S;Bossers,A;Koch,G;Bergervoet,S |
| EPI_ISL_269602 | A/Lesser_Black-backed_Gull/Netherlands/1/2016 | 2016-12-20 | Erasmus Medical Center | Erasmus Medical Center | Poen,MJ;VanDerJeugd,HP;Vuong,O;Scheuer,RD;Kleyheeg,E;Bestebroer,TM;Begeman,L;vandenBrand,JMA;Kuiken,T;Fouchier,RAM |
| EPI_ISL_252194 | A/glaucous-winged gull/Southcentral Alaska/16AS00020/2016 | 2016-03-03 | Not listed | Other Database Import | Das,SR;Halpin,RA;Lin,X;Simenauer,A;Akopov,A;Fedorova,N;Puri,V;Stockwell,T;Amedeo,P;Katzel,D;Schobel,S;Shrivastava,S;Hill,N;Bao,Y;Sanders,R;Zhdanov,S;Kiryutin,B;Lipman,DJ;Tatusova,T;Runstadler,J |
| EPI_ISL_289500 | A/Great Black-backed Gull/Netherlands/1/2017 | 2017-12-18 | Erasmus Medical Center | Erasmus Medical Center | Poen,MJ;Bestebroer,TM;Kelder,L;Scheuer,RD;Koopmans,MPG;VanDerJeugd,HP;Fouchier,RAM |
| EPI_ISL_269598 | A/Great_Black-backed_Gull/Netherlands/3/2016 | 2016-11-23 | Erasmus Medical Center | Erasmus Medical Center | Poen,MJ;VanDerJeugd,HP;Vuong,O;Scheuer,RD;Kleyheeg,E;Bestebroer,TM;Begeman,L;vandenBrand,JMA;Kuiken,T;Fouchier,RAM |
| EPI_ISL_255892 | A/Great Black-backed Gull/Netherlands/2/2016 | 2016-11-23 | Erasmus Medical Center | Erasmus Medical Center | Poen,MJ;VanDerJeugd,HP;Vuong,O;Scheuer,RD;Kleyheeg,E;Lexmond,P;Eggink,WD;Müskens,GJDM;Bestebroer,TM;Koopmans,MPG;Kuiken,T;Fouchier,RAM |
| EPI_ISL_268619 | A/Bl_H_gull/NL-Slootdorp/16014102-002/2016 | 2016-11-11 | Wageningen Bioveterinary Research | Wageningen Bioveterinary Research | Beerens,N;Heutink,R;Harders,F;Verschuren-Pritz,S;Bossers,A;Koch,G;Bergervoet,S |
| EPI_ISL_231685 | A/black-headed gull/Tyva/41/2016 | 2016-05-25 | State Research Center of Virology and Biotechnology (VECTOR) | WHO National Influenza Centre Russian Federation | Fadeev,A;Komissarov,A;Egorova,A;Sintsova,K;Musaeva,T;Susloparov,I;Marchenko,V;Ryzhikov,A |
| EPI_ISL_285618 | A/Ostrich/South_Africa/S2017/08_0362_P7/2017 | 2017-08-22 | Western Cape Provincial Veterinary Laboratory | National Institute of Communicable Diseases | Treurnicht,FK |
| EPI_ISL_285608 | A/Ostrich/South_Africa/S2017/08_0362_P8_33/2017 | 2017-08-22 | Western Cape Provincial Veterinary Laboratory | National Institute of Communicable Diseases | Treurnicht,FK |
| EPI_ISL_285509 | A/Ostrich/South_Africa/S2017/08_0362_P11/2017 | 2017-08-22 | Western Cape Provincial Veterinary Laboratory | National Institute of Communicable Diseases | Treurnicht,FK |
| EPI_ISL_284198 | A/Ostrich/South_Africa/S2017/08_0361_P17/2017 | 2017-08-22 | Western Cape Provincial Veterinary Laboratory | National Institute of Communicable Diseases | Treurnicht,FK |
| EPI_ISL_283970 | A/Ostrich/Guangxi/GX-1/2017 (H5N6) | 2017-06-28 | South China Agricultural University | South China Agricultural University | Jiahao,Zhang;Guangjie,Lao;Zhengji,Wei;Guanming,Su;Bo,Li;Wenbao,Qi;Ming,Liao |
| EPI_ISL_257657 | A/Pavo cristatus/Jiangxi/JA1/2016 | 2016-02-27 | Not listed | Other Database Import | Li,M;Zhao,N;Luo,J;Li,Y;Chen,L;Ma,J;Zhao,L;Yuan,G;Wang,C;Wang,Y;Liu,Y;He,H |
| EPI_ISL_301070 | A/swan/Poland/99/2017 | 2017-02-09 | Not listed | National Veterinary Research Institut Poland, PIWet-PIB | Swieton,E;Smietanka,K |
| EPI_ISL_300746 | A/swan/Poland/81/2017 | 2017-02-02 | Not listed | National Veterinary Research Institut Poland, PIWet-PIB | Swieton,E;Smietanka,K |
| EPI_ISL_285627 | A/Swan/South_Africa/S2017/08_0517_P2/2017 | 2017-08-28 | Western Cape Provincial Veterinary Laboratory | National Institute of Communicable Diseases | Treurnicht,FK |
| EPI_ISL_260797 | A/tundra swan/Niigata/10/2016 | 2016-12-16 | Not listed | Hokkaido University | MasatoshiOkamatsu |
| EPI_ISL_260796 | A/tundra swan/Niigata/9/2016 | 2016-12-10 | Not listed | Hokkaido University | MasatoshiOkamatsu |
| EPI_ISL_260795 | A/tundra swan/Niigata/7/2016 | 2016-12-14 | Not listed | Hokkaido University | MasatoshiOkamatsu |
| EPI_ISL_260789 | A/whooper swan/Aomori/6/2016 | 2016-12-12 | Not listed | Hokkaido University | MasatoshiOkamatsu |
| EPI_ISL_260786 | A/whooper swan/Hokkaido/X15/2017 | 2017-02-22 | Not listed | Hokkaido University | MasatoshiOkamatsu |
| EPI_ISL_260785 | A/whooper swan/Hokkaido/X14/2017 | 2017-01-22 | Not listed | Hokkaido University | MasatoshiOkamatsu |
| EPI_ISL_260783 | A/whooper swan/Hokkaido/X4/2016 | 2016-12-02 | Not listed | Hokkaido University | MasatoshiOkamatsu |
| EPI_ISL_260779 | A/whooper swan/Iwate/18/2016 | 2017-02-13 | Not listed | Hokkaido University | MasatoshiOkamatsu |
| EPI_ISL_260776 | A/whooper swan/Iwate/14/2016 | 2016-12-27 | Not listed | Hokkaido University | MasatoshiOkamatsu |
| EPI_ISL_260774 | A/whooper swan/Iwate/10/2016 | 2016-12-25 | Not listed | Hokkaido University | MasatoshiOkamatsu |
| EPI_ISL_260773 | A/whooper swan/Iwate/7/2016 | 2016-12-22 | Not listed | Hokkaido University | MasatoshiOkamatsu |
| EPI_ISL_260771 | A/whooper swan/Iwate/4/2016 | 2016-12-17 | Not listed | Hokkaido University | MasatoshiOkamatsu |
| EPI_ISL_260768 | A/black swan/Akita/3/2016 | 2016-11-17 | Not listed | Hokkaido University | MasatoshiOkamatsu |
| EPI_ISL_260766 | A/mute swan/Ibaraki/5/2016 | 2016-12-14 | Not listed | Hokkaido University | MasatoshiOkamatsu |
| EPI_ISL_255181 | A/swan/Italy/17VIR537-2/2017 | 2017-01-19 | Istituto Zooprofilattico Sperimentale Delle Venezie | Istituto Zooprofilattico Sperimentale Delle Venezie | Zecchin,B;Fusaro,A;Milani,A;Schivo,A;Salviato,A;Zamperin,G;Marciano,S;Ormelli,S;Terregino,C;Monne,I |
| EPI_ISL_247401 | A/mute swan/Ibaraki/221C/2016 | 2016-12-14 | National Institute of Animal Health | National Institute of Animal Health | Takehiko,S |
| EPI_ISL_247400 | A/mute swan/Ibaraki/210T/2016 | 2016-12-10 | National Institute of Animal Health | National Institute of Animal Health | Takehiko,S |
| EPI_ISL_247371 | A/mute swan/Ibaraki/219T/2016 | 2016-12-14 | National Institute of Animal Health | National Institute of Animal Health | Takehiko,S |
| EPI_ISL_240893 | A/swan/Germany-SN/R10645/2016 | 2016-12-13 | Not listed | Friedrich-Loeffler-Institut | Starick,E |
| EPI_ISL_240628 | A/mute swan/Kyoto/1T/2016 | 2016-12-22 | National Institute of Animal Health | National Institute of Animal Health | Uchida,Y |
| EPI_ISL_240627 | A/mute swan/Ibaraki/211T/2016 | 2016-12-12 | National Institute of Animal Health | National Institute of Animal Health | Uchida,Y |
| EPI_ISL_240609 | A/mute swan/Kyoto/3T/2016 | 2016-12-22 | National Institute of Animal Health | National Institute of Animal Health | Uchida,Y |
| EPI_ISL_240607 | A/mute swan/Kyoto/4T/2016 | 2016-12-22 | National Institute of Animal Health | National Institute of Animal Health | Uchida,Y |
| EPI_ISL_240011 | A/mute swan/Ibaraki/254C/2016 | 2016-12-18 | National Institute of Animal Health | National Institute of Animal Health | Mine,J |
| EPI_ISL_240006 | A/mute swan/Ibaraki/247C/2016 | 2016-12-16 | National Institute of Animal Health | National Institute of Animal Health | Mine,J |
| EPI_ISL_240003 | A/mute swan/Ibaraki/250T/2016 | 2016-12-17 | National Institute of Animal Health | National Institute of Animal Health | Mine,J |
| EPI_ISL_295493 | A/Cygnus atratus/Hubei/2Z2-O/2016 | 2016-12-28 | Not listed | Other Database Import | Ma,L;Zhao,L;Wang,R;Chen,Q |
| EPI_ISL_294413 | A/black swan/Aichi/2312T002/2016 | 2016-12-04 | Not listed | Other Database Import | Soda,K;Usui,T;Ito,H;Ozaki,H;Yamaguchi,T;Ito,T |
| EPI_ISL_268618 | A/Bk_swan/NL-Den Oever/16013973-002/2016 | 2016-11-10 | Wageningen Bioveterinary Research | Wageningen Bioveterinary Research | Beerens,N;Heutink,R;Harders,F;Verschuren-Pritz,S;Bossers,A;Koch,G;Bergervoet,S |
| EPI_ISL_266940 | A/tundra swan/Tottori/3111S002/2016 | 2016-11-20 | Not listed | Other Database Import | Soda,K;Usui,T;Ito,H;Ozaki,H;Yamaguchi,T;Ito,T |
| EPI_ISL_266939 | A/tundra swan/Tottori/3111S001/2016 | 2016-11-20 | Not listed | Other Database Import | Soda,K;Usui,T;Ito,H;Ozaki,H;Yamaguchi,T;Ito,T |
| EPI_ISL_256524 | A/whooper swan/Tochigi/1/2017 | 2017-02-03 | Not listed | Hokkaido University | MasatoshiOkamatsu |
| EPI_ISL_256508 | A/whooper swan/Iwate/5/2016 | 2016-12-18 | Not listed | Hokkaido University | MasatoshiOkamatsu |
| EPI_ISL_256505 | A/whooper swan/Aomori/9/2016 | 2016-12-29 | Graduate School of Veterinary Medicine, Hokkaido University | Hokkaido University | MasatoshiOkamatsu |
| EPI_ISL_256499 | A/whooper swan/Hokkaido/X13/2017 | 2017-01-18 | Graduate School of Veterinary Medicine, Hokkaido University | Hokkaido University | MasatoshiOkamatsu |
| EPI_ISL_256498 | A/whooper swan/Hokkaido/X12/2017 | 2017-01-15 | Graduate School of Veterinary Medicine, Hokkaido University | Hokkaido University | MasatoshiOkamatsu |
| EPI_ISL_244523 | A/whooper swan/Korea/Gangjin 48/2016 | 2016-11-20 | Not listed | Other Database Import | Jeong,J;Woo,C |
| EPI_ISL_309195 | A/Cygnus olor/Belgium/2967/2017 | 2017-03-21 | Not listed | Other Database Import | VanBorm,S;Vandenbussche,F;Mathijs,E;Lambrecht,B;Steensels,M |
| EPI_ISL_308777 | A/mute swan/Croatia/85/2017 | 2017-02-14 | Not listed | Croatian Veterinary Institute | Savić,Vladimir |
| EPI_ISL_295704 | A/mute swan/Ibaraki/0803-208/2016 | 2016-12-10 | Not listed | Other Database Import | Soda,K;Usui,T;Ito,H;Ozaki,H;Yamaguchi,T;Ito,T |
| EPI_ISL_290306 | A/mute swan/Hyogo/2801ITM011/2017 | 2017-01-17 | Not listed | Other Database Import | Soda,K;Usui,T;Ito,H;Ozaki,H;Murase,T;Yamaguchi,T;Ito,T |
| EPI_ISL_290305 | A/mute swan/Hyogo/2801ITM010/2017 | 2017-01-17 | Not listed | Other Database Import | Soda,K;Usui,T;Ito,H;Ozaki,H;Murase,T;Yamaguchi,T;Ito,T |
| EPI_ISL_290302 | A/mute swan/Hyogo/2801ITM009/2017 | 2017-01-17 | Not listed | Other Database Import | Soda,K;Usui,T;Ito,H;Ozaki,H;Murase,T;Yamaguchi,T;Ito,T |
| EPI_ISL_290300 | A/mute swan/Hyogo/2801ITM007/2017 | 2017-01-17 | Not listed | Other Database Import | Soda,K;Usui,T;Ito,H;Ozaki,H;Murase,T;Yamaguchi,T;Ito,T |
| EPI_ISL_290299 | A/mute swan/Hyogo/2801ITM006/2017 | 2017-01-15 | Not listed | Other Database Import | Soda,K;Usui,T;Ito,H;Ozaki,H;Murase,T;Yamaguchi,T;Ito,T |
| EPI_ISL_290298 | A/mute swan/Hyogo/2801ITM005/2017 | 2017-01-15 | Not listed | Other Database Import | Soda,K;Usui,T;Ito,H;Ozaki,H;Murase,T;Yamaguchi,T;Ito,T |
| EPI_ISL_290295 | A/mute swan/Hyogo/2801ITM002/2017 | 2017-01-13 | Not listed | Other Database Import | Soda,K;Usui,T;Ito,H;Ozaki,H;Murase,T;Yamaguchi,T;Ito,T |
| EPI_ISL_290156 | A/mute swan/Ibaraki/0803_210/2016 | 2016-12-11 | Not listed | Other Database Import | Soda,K;Usui,T;Ito,H;Ozaki,H;Yamagichi,T;Ito,T;Yamaguchi,T |
| EPI_ISL_289173 | A/mute swan/Shimane/3211A001/2017 | 2017-11-05 | Not listed | Other Database Import | Soda,K;Ito,H;Usui,T;Ozaki,H;Murase,T;Yamaguchi,T;Ito,T |
| EPI_ISL_287565 | A/M_Swan/NL-Groningen/16015826-001/2016 | 2016-12-13 | Wageningen Bioveterinary Research | Wageningen Bioveterinary Research | Beerens,N;Heutink,R;Harders,F;Verschuren-Pritz,S;Bossers,A;Koch,G;Bergervoet,S |
| EPI_ISL_268974 | A/mute swan/Czech Republic/1848-17_2/2017 (H5N8) | 2017-02-06 | State Veterinary Institute Prague | State Veterinary Institute Prague | Nagy,A |
| EPI_ISL_268961 | A/mute swan/Czech Republic/1640-17/2017 (H5N8) | 2017-02-02 | State Veterinary Institute Prague | State Veterinary Institute Prague | Nagy,A |
| EPI_ISL_268959 | A/mute swan/Czech Republic/1576-17_C/2017 (H5N8) | 2017-01-31 | State Veterinary Institute Prague | State Veterinary Institute Prague | Nagy,A |
| EPI_ISL_268954 | A/mute swan/Czech Republic/1339-17/2017 (H5N8) | 2017-01-31 | State Veterinary Institute Prague | State Veterinary Institute Prague | Nagy,A |
| EPI_ISL_268953 | A/mute swan/Czech Republic/1337-17/2017 (H5N8) | 2017-01-27 | State Veterinary Institute Prague | State Veterinary Institute Prague | Nagy,A |
| EPI_ISL_268952 | A/mute swan/Czech Republic/1331-17_1/2017 (H5N8) | 2017-01-23 | State Veterinary Institute Prague | State Veterinary Institute Prague | Nagy,A |
| EPI_ISL_268945 | A/mute swan/Czech Republic/1171-17/2017 (H5N8) | 2017-01-22 | State Veterinary Institute Prague | State Veterinary Institute Prague | Nagy,A |
| EPI_ISL_268941 | A/mute swan/Czech Republic/1060-17/2017 (H5N8) | 2017-01-19 | State Veterinary Institute Prague | State Veterinary Institute Prague | Nagy,A |
| EPI_ISL_268939 | A/mute swan/Czech Republic/987-17_2/2017 (H5N8) | 2017-01-20 | State Veterinary Institute Prague | State Veterinary Institute Prague | Nagy,A |
| EPI_ISL_268938 | A/mute swan/Czech Republic/967-17/2017 (H5N8) | 2017-01-19 | State Veterinary Institute Prague | State Veterinary Institute Prague | Nagy,A |
| EPI_ISL_268928 | A/mute swan/Czech Republic/54-17_1/2017 (H5N8) | 2017-01-02 | State Veterinary Institute Prague | State Veterinary Institute Prague | Nagy,A |
| EPI_ISL_268662 | A/M_Swan/NL-Roggebotsluis/16014462-019/2016 | 2016-11-17 | Wageningen Bioveterinary Research | Wageningen Bioveterinary Research | Beerens,N;Heutink,R;Harders,F;Verschuren-Pritz,S;Bossers,A;Koch,G;Bergervoet,S |
| EPI_ISL_256501 | A/mute swan/Aomori/4/2016 | 2016-12-06 | Graduate School of Veterinary Medicine, Hokkaido University | Hokkaido University | MasatoshiOkamatsu |
| EPI_ISL_256462 | A/Mute_swan/Hungary/5879/2017 | 2017-02-14 | National Food Chain Safety Office Veterinary Diagnostic Directorate Laboratory for Molecular Biology | Central Agricultural Office Veterinary Diagnostic Directorate | Dan,A |
| EPI_ISL_255202 | A/Mute swan/Hungary/3139/2017 | 2017-01-26 | Central Agricultural Office Veterinary Diagnostic Directorate | Central Agricultural Office Veterinary Diagnostic Directorate | Dan,A |
| EPI_ISL_250920 | A/mute swan/Czech Republic/2031-17/2017 (H5N5) | 2017-02-09 | Not listed | State Veterinary Institute Prague | Nagy,Alexander |
| EPI_ISL_250885 | A/mute swan/Czech Republic/54-17_2/2017 (H5N8) | 2017-01-02 | Not listed | State Veterinary Institute Prague | Nagy,Alexander |
| EPI_ISL_248664 | A/mute swan/Croatia/61/2017 | 2017-02-02 | Not listed | Croatian Veterinary Institute | Savić,Vladimir |
| EPI_ISL_243698 | A/mute swan/Croatia/42/2017 | 2017-01-20 | Not listed | Croatian Veterinary Institute | Savić,Vladimir |
| EPI_ISL_241953 | A/mute swan/Croatia/15/2017 | 2017-01-10 | Not listed | Croatian Veterinary Institute | Savić,Vladimir |
| EPI_ISL_239069 | A/mute swan/Croatia/85/2016 | 2016-11-28 | Not listed | Croatian Veterinary Institute | Savić,Vladimir |
| EPI_ISL_237730 | A/mute_swan/Hungary/51049/2016 | 2016-10-19 | National Food Chain Safety Office Veterinary Diagnostic Directorate Laboratory for Molecular Biology | Central Agricultural Office Veterinary Diagnostic Directorate | Ronai,Z;Ursu,K;Balint,A;Szalay,A;Thuma,A;Gyuris,E;Erdelyi,K;Dan,A |
| EPI_ISL_300783 | A/turkey/Poland/89/2016 | 2016-12-22 | Not listed | National Veterinary Research Institut Poland, PIWet-PIB | Swieton,E;Smietanka,K |
| EPI_ISL_300749 | A/turkey/Poland/81/2016 | 2016-12-19 | Not listed | National Veterinary Research Institut Poland, PIWet-PIB | Swieton,E;Smietanka,K |
| EPI_ISL_300698 | A/turkey/Poland/54/2017 | 2017-01-18 | National Veterinary Research Institut Poland, PIWet-PIB | National Veterinary Research Institut Poland, PIWet-PIB | Swieton,E;Smietanka,K |
| EPI_ISL_298649 | A/turkey/Israel/184/2017 | 2017-02-05 | Not listed | Other Database Import | Shkoda,I;Lapin,K;Simanov,L;Lublin,A |
| EPI_ISL_273847 | A/turkey/Italy/17VIR5878-3/2017 | 2017-07-23 | Istituto Zooprofilattico Sperimentale Delle Venezie | Istituto Zooprofilattico Sperimentale Delle Venezie | Zecchin,B;Fusaro,A;Zamperin,G;Schivo,A;Salviato,A;Marciano,S;Ormelli,S;Terregino,C;Monne,I |
| EPI_ISL_268972 | A/turkey/Czech Republic/1767-17_2/2017 (H5N8) | 2017-02-06 | State Veterinary Institute Prague | State Veterinary Institute Prague | Nagy,A |
| EPI_ISL_257731 | A/turkey/Germany-BB/R377ff/2017 | 2017-01-17 | Not listed | Friedrich-Loeffler-Institut | Starick,E |
| EPI_ISL_255185 | A/turkey/Italy/17VIR973-2/2017 | 2017-02-01 | Istituto Zooprofilattico Sperimentale Delle Venezie | Istituto Zooprofilattico Sperimentale Delle Venezie | Zecchin,B;Fusaro,A;Milani,A;Schivo,A;Salviato,A;Zamperin,G;Marciano,S;Ormelli,S;Terregino,C;Monne,I |
| EPI_ISL_255183 | A/turkey/Italy/17VIR576-11/2017 | 2017-01-23 | Istituto Zooprofilattico Sperimentale Delle Venezie | Istituto Zooprofilattico Sperimentale Delle Venezie | Zecchin,B;Fusaro,A;Milani,A;Schivo,A;Salviato,A;Zamperin,G;Marciano,S;Ormelli,S;Terregino,C;Monne,I |
| EPI_ISL_253036 | A/turkey/England/003778/2017 | 2017-01-15 | Animal and Plant Health Agency (APHA) | Animal and Plant Health Agency (APHA) | Seekings,James;Ellis,Richard;Brookes,SharonM;Reid,Scott;Essen,Stephen;Brown,IanH |
| EPI_ISL_249681 | A/turkey/Germany-BB/R234ff/2017 | 2017-01-09 | Not listed | Friedrich-Loeffler-Institut | Starick,E |
| EPI_ISL_248666 | A/bronze turkey/Czech Republic/1414-17/2017 (H5N8) | 2017-01-30 | Not listed | State Veterinary Institute Prague | Nagy,Alexander |
| EPI_ISL_247721 | A/turkey/Rostov-on-Don/11/2017 | 2017-01-01 | Not listed | State Research Center of Virology and Biotechnology (VECTOR) | Susloparov,I;Goncharova,N;Kolosova,N;Marchenko,V;Ryzhikov,A |
| EPI_ISL_239801 | A/turkey/England/052131/2016 | 2016-12-15 | Animal and Plant Health Agency (APHA) | Animal and Plant Health Agency (APHA) | Seekings,James;Ellis,Richard;Brookes,SharonM;Reid,Scott;Essen,Stephen;Brown,IanH |
| EPI_ISL_237731 | A/domestic_turkey/Hungary/53433/2016 | 2016-11-02 | National Food Chain Safety Office Veterinary Diagnostic Directorate Laboratory for Molecular Biology | Central Agricultural Office Veterinary Diagnostic Directorate | Ronai,Z;Ursu,K;Balint,A;Szalay,A;Thuma,A;Gyuris,E;Erdelyi,K;Dan,A |
| EPI_ISL_283971 | A/Quail/Guangxi/GX-2/2017 (H5N6) | 2017-07-15 | South China Agricultural University | South China Agricultural University | Jiahao,Zhang;Guangjie,Lao;Zhengji,Wei;Guanming,Su;Bo,Li;Wenbao,Qi;Ming,Liao |
| EPI_ISL_298648 | A/great egret/Israel/1088/2016 | 2016-12-25 | Not listed | Other Database Import | Shkoda,I;Lapin,K;Simanov,L;Lublin,A |
| EPI_ISL_297141 | A/quail/Egypt/HASHM5/2016 | 2016-01 | Not listed | Other Database Import | Ahmed,HA;Tolba,HMN;Elsohaby,I;AbouElez,RMM |
| EPI_ISL_285653 | A/Pigeon/South_Africa/S2017/08_0323_P1/2017 | 2017-08-18 | Western Cape Provincial Veterinary Laboratory | National Institute of Communicable Diseases | Treurnicht,FK |
| EPI_ISL_285611 | A/Wildbirds_Guineafowl_Makou_Egyptian Geese/South_Africa/S2017/08_0275_P1/2017 | 2017-08-16 | Western Cape Provincial Veterinary Laboratory | National Institute of Communicable Diseases | Treurnicht,FK |
| EPI_ISL_284196 | A/Pigeon/South_Africa/S2017/08_0324_24/2017 | 2017-08-18 | Western Cape Provincial Veterinary Laboratory | National Institute of Communicable Diseases | Treurnicht,FK |
| EPI_ISL_279039 | A/crane/Kagoshima/KU-52/2016 | 2016-12-11 | Not listed | Kagoshima University | MakatoOzawa |
| EPI_ISL_279037 | A/crane/Kagoshima/KU-46/2016 | 2016-12-05 | Not listed | Kagoshima University | MakatoOzawa |
| EPI_ISL_279033 | A/crane/Kagoshima/KU-37/2016 | 2016-11-28 | Not listed | Kagoshima University | MakatoOzawa |
| EPI_ISL_279028 | A/crane/Kagoshima/KU-28/2016 | 2016-11-24 | Not listed | Kagoshima University | MakatoOzawa |
| EPI_ISL_279026 | A/crane/Kagoshima/KU-26/2016 | 2016-11-23 | Not listed | Kagoshima University | MakatoOzawa |
| EPI_ISL_279020 | A/crane/Kagoshima/KU-16/2016 | 2016-11-22 | Not listed | Kagoshima University | MakatoOzawa |
| EPI_ISL_279019 | A/crane/Kagoshima/KU-15/2016 | 2016-11-22 | Not listed | Kagoshima University | MakatoOzawa |
| EPI_ISL_279018 | A/crane/Kagoshima/KU-12/2016 | 2016-11-21 | Not listed | Kagoshima University | MakatoOzawa |
| EPI_ISL_268979 | A/quail/Czech Republic/2063-17_1/2017 (H5N8) | 2017-02-11 | State Veterinary Institute Prague | State Veterinary Institute Prague | Nagy,A |
| EPI_ISL_268663 | A/Magpie/NL-Volendam/16014331-002/2016 | 2016-11-16 | Wageningen Bioveterinary Research | Wageningen Bioveterinary Research | Beerens,N;Heutink,R;Harders,F;Verschuren-Pritz,S;Bossers,A;Koch,G;Bergervoet,S |
| EPI_ISL_256506 | A/grey-faced buzzard/Aomori/10/2016 | 2016-12-28 | Not listed | Hokkaido University | MasatoshiOkamatsu |
| EPI_ISL_243085 | A/wigeon/Italy/16VIR9616-3/2016 | 2016-12-29 | Istituto Zooprofilattico Sperimentale Delle Venezie | Istituto Zooprofilattico Sperimentale Delle Venezie | Silvia,Ormelli;Sabrina,Marciano;Alessia,Schivo;Annalisa,Salviato;Adelaide,Milani;Gianpiero,Zamperini;Bianca,Zecchin;Alice,Fusaro;Calogero,Terregino;Isabella,Monne |
| EPI_ISL_242902 | A/wigeon/Italy/17VIR57-3/2017 | 2017-01-03 | Istituto Zooprofilattico Sperimentale Delle Venezie | Istituto Zooprofilattico Sperimentale Delle Venezie | Silvia,Ormelli;Sabrina,Marciano;Alessia,Schivo;Annalisa,Salviato;Adelaide,Milani;Gianpiero,Zamperin;Bianca,Zecchin;Alice,Fusaro;Calogero,Terregino;Isabella,Monne |
| EPI_ISL_239573 | A/crane/Kagoshima/KU-4/2016(H5N6) | 2016-11-19 | Kagoshima University | Kagoshima University | MakatoOzawa |
| EPI_ISL_244744 | A/lion/Hubei/1-2/2016 | 2016-04-12 | Not listed | Other Database Import | Chen,Q;Wang,H;Zhao,L;Ma,L;Wang,R;Lei,Y;Li,Y;Yang,G;Chen,J;Chen,G;Li,L;Jin,T;Li,J;Liu,X;Xu,X;Wong,G;Liu,L;Liu,Y;Shi,W;Bi,Y;Gao,GF |
| EPI_ISL_262950 | A/swine/Nigeria/77/2016 | 2016-01-30 | National Veterinary Research Institute | Friedrich-Loeffler-Institut | Meseko,Clement;Starick,Elke |
| EPI_ISL_262932 | A/swine/Nigeria/55/2016 | 2016-01-09 | National Veterinary Research Institute | Friedrich-Loeffler-Institut | Meseko,Clement;Starick,Elke |
| EPI_ISL_309422 | A/environment/South Carolina/UGAI17-2505/2017 | 2017-05-12 | Not listed | Other Database Import | Tan,G;Pickett,B;Fedorova,N;Amedeo,P;Isom,R;Hu,L;Christensen,J;Durbin,A;Williams,T;Arumemi,F;Poulson,R;Bao,Y;Sanders,R;Zhdanov,S;Kiryutin,B;Lipman,DJ;Tatusova,T;Hatcher,E;Stallknecht,D |
| EPI_ISL_307668 | A/environment/New Jersey/UGAI17-2155/2017 | 2017-05-14 | Not listed | Other Database Import | Tan,G;Pickett,B;Fedorova,N;Amedeo,P;Isom,R;Hu,L;Christensen,J;Durbin,A;Williams,T;Arumemi,F;Poulson,R;Bao,Y;Sanders,R;Zhdanov,S;Kiryutin,B;Lipman,DJ;Tatusova,T;Hatcher,E;Stallknecht,D |
| EPI_ISL_307666 | A/environment/Georgia/UGAI17-2179/2017 | 2017-05-08 | Not listed | Other Database Import | Tan,G;Pickett,B;Fedorova,N;Amedeo,P;Isom,R;Hu,L;Christensen,J;Durbin,A;Williams,T;Arumemi,F;Poulson,R;Bao,Y;Sanders,R;Zhdanov,S;Kiryutin,B;Lipman,DJ;Tatusova,T;Hatcher,E;Stallknecht,D |
| EPI_ISL_293489 | A/environment/Saga/4/2017 | 2017-02-04 | Not listed | Other Database Import | Soda,K;Usui,T;Ito,H;Ozaki,H;Yamaguchi,T;Ito,T |
| EPI_ISL_293488 | A/environment/Hokkaido/1-8/2016 | 2016-12-16 | Not listed | Other Database Import | Soda,K;Usui,T;Ito,H;Ozaki,H;Yamaguchi,T;Ito,T |
| EPI_ISL_293487 | A/environment/Aomori/2-1/2016 | 2016-12-02 | Not listed | Other Database Import | Soda,K;Usui,T;Ito,H;Ozaki,H;Yamaguchi,T;Ito,T |
| EPI_ISL_290158 | A/environment/Gifu/21/2017 | 2017-01-14 | Not listed | Other Database Import | Soda,K;Usui,T;Ito,H;Ozaki,H;Yamagichi,T;Ito,T;Yamaguchi,T |
| EPI_ISL_244522 | A/environment/Korea/W544/2016 | 2016-11-18 | Not listed | Other Database Import | Si,YJ;Lee,IW;Kim,EH;Kim,YI;Kwon,HI;Park,SJ;Nguyen,HD;Kim,SM;Kwon,JJ;Choi,WS;Beak,YH;Song,MS;Kim,CJ;Webby,RJ;Choi,YK;Si,Y-J;Kwon,H-I;Lee,I-W;Dihn,HN;Kim,Y-I;Choi,Y-K |
| EPI_ISL_244520 | A/environment/Korea/W542/2016 | 2016-11-18 | Not listed | Other Database Import | Si,YJ;Lee,IW;Kim,EH;Kim,YI;Kwon,HI;Park,SJ;Nguyen,HD;Kim,SM;Kwon,JJ;Choi,WS;Beak,YH;Song,MS;Kim,CJ;Webby,RJ;Choi,YK;Si,Y-J;Kwon,H-I;Lee,I-W;Dihn,HN;Kim,Y-I;Choi,Y-K |
| EPI_ISL_221719 | A/environment/Guangdong/01.01 SZSGXJK006-E/2016 | 2016-01-01 | Institute of Microbiology, Chinese Academy of Sciences | Institute of Microbiology, Chinese Academy of Sciences | Bi,Y |
| EPI_ISL_304647 | A/reassortant/IDCDC-RG42A(Sichuan/26221/2014 X Puerto Rico/8/1934) | 2017 | Not listed | Other Database Import | Wong,T;Creanga,A;Wang,L;Barnes,J;Jernigan,D;Wentworth,D;Chen,L-M |
| EPI_ISL_284650 | A/Anhui/33162/2016 | 2016-04-28 | Anhui Provincial Center for Disease Control and Prevention | WHO Chinese National Influenza Center | Zhang,Ye;Li,Xiyan;He,Jun;Wang,Dayan |
| EPI_ISL_232493 | A/Egypt/N04915/2014 NIBRG-306 | 2016-08-31 | Not listed | National Institute for Biological Standards and Control (NIBSC) | Nicolson,C |
| EPI_ISL_207048 | A/Shenzhen/TH003/2016(H5N6) | 2016-01-15 | CAS Key Laboratory of Pathogenic Microbiology and Immunology, Institute of Microbiology, Chinese Academy of Sciences | Institute of Microbiology, Chinese Academy of Sciences | Bi,Y |
| EPI_ISL_293952 | A/duck/France/160927/2016 | 2017-07-21 | Anses (Ploufragan-Plouzané) | ANSES Agence Nationale De Securite Sanitaire De L’alimentation | Briand,FX |
| EPI_ISL_290294 | A/Heron/Vietnam/QuangBinh/LBM0910/2016(H5N6) | 2016-09-10 | Not listed | Other Database Import | Pham,HM;Pham,HK;Nguyen,VK |
| EPI_ISL_260788 | A/goshawk/Aomori/5/2016 | 2016-12-12 | Not listed | Hokkaido University | MasatoshiOkamatsu |
| EPI_ISL_260763 | A/mute swan/Ibaraki/2/2016 | 2016-12-14 | Not listed | Hokkaido University | MasatoshiOkamatsu |
| EPI_ISL_258415 | A/grey heron/Germany-TH/R1125/2017 | 2017-02-04 | Not listed | Friedrich-Loeffler-Institut | Starick,E |
| EPI_ISL_256457 | A/Duck/Hungary/55764/2016 | 2016-11-11 | National Food Chain Safety Office Veterinary Diagnostic Directorate Laboratory for Molecular Biology | Central Agricultural Office Veterinary Diagnostic Directorate | Dan,A |
| EPI_ISL_256298 | A/gadwall/Kurgan/2442/2016 | 2016-08-27 | Not listed | State Research Center of Virology and Biotechnology (VECTOR) | Susloparov,I;Goncharova,N;Kolosova,N;Marchenko,V;Ryzhikov,A |
| EPI_ISL_255216 | A/Mallard/Hungary/5821/2017 | 2017-02-13 | Central Agricultural Office Veterinary Diagnostic Directorate | Central Agricultural Office Veterinary Diagnostic Directorate | Dan,A |
| EPI_ISL_255214 | A/Chicken/Hungary/1751/2017 | 2017-01-17 | Central Agricultural Office Veterinary Diagnostic Directorate | Central Agricultural Office Veterinary Diagnostic Directorate | Dan,A |
| EPI_ISL_255207 | A/Mute swan/Hungary/5316/2017 | 2017-02-09 | Central Agricultural Office Veterinary Diagnostic Directorate | Central Agricultural Office Veterinary Diagnostic Directorate | Dan,A |
| EPI_ISL_255200 | A/Mute swan/Hungary/2508/2017 | 2017-01-22 | Central Agricultural Office Veterinary Diagnostic Directorate | Central Agricultural Office Veterinary Diagnostic Directorate | Dan,A |
| EPI_ISL_255197 | A/Mute swan/Hungary/1955/2017 | 2017-01-18 | Central Agricultural Office Veterinary Diagnostic Directorate | Central Agricultural Office Veterinary Diagnostic Directorate | Dan,A |
| EPI_ISL_255193 | A/Duck/Hungary/984/2017 | 2017-01-11 | Central Agricultural Office Veterinary Diagnostic Directorate | Central Agricultural Office Veterinary Diagnostic Directorate | Dan,A |
| EPI_ISL_255192 | A/Goose/Hungary/982/2017 | 2017-01-11 | Central Agricultural Office Veterinary Diagnostic Directorate | Central Agricultural Office Veterinary Diagnostic Directorate | Dan,A |
| EPI_ISL_255190 | A/Mute swan/Hungary/119/2017 | 2017-01-02 | Central Agricultural Office Veterinary Diagnostic Directorate | Central Agricultural Office Veterinary Diagnostic Directorate | Dan,A |
| EPI_ISL_247717 | A/long-eared owl/Voronezh/16/2017 | 2017-01-06 | Not listed | State Research Center of Virology and Biotechnology (VECTOR) | Susloparov,I;Goncharova,N;Kolosova,N;Marchenko,V;Ryzhikov,A |
| EPI_ISL_247716 | A/Ural owl/Voronezh/14/2017 | 2017-01-06 | Not listed | State Research Center of Virology and Biotechnology (VECTOR) | Susloparov,I;Goncharova,N;Kolosova,N;Marchenko,V;Ryzhikov,A |
| EPI_ISL_243677 | A/black swan/Ibaraki/345T/2017 | 2017-01-17 | Not listed | National Institute of Animal Health | TakehikoSaito |
| EPI_ISL_243676 | A/black swan/Ibaraki/357T/2017 | 2017-01-22 | Not listed | National Institute of Animal Health | TakehikoSaito |
| EPI_ISL_243675 | A/black swan/Ibaraki/348C/2017 | 2017-01-18 | Not listed | National Institute of Animal Health | TakehikoSaito |
| EPI_ISL_241777 | A/great crested grebe/Ibaraki/298C/2016 | 2016-12-28 | Not listed | National Institute of Animal Health | Saito,T;Takemae,N |
| EPI_ISL_241776 | A/black headed gull/Ibaraki/289T/2016 | 2016-12-26 | Not listed | National Institute of Animal Health | Saito,T;Takemae,N |
| EPI_ISL_241769 | A/mute swan/Ibaraki/248C/2016 | 2016-12-17 | Not listed | National Institute of Animal Health | Saito,T;Takemae,N |
| EPI_ISL_241768 | A/mute swan/Ibaraki/270T/2016 | 2016-12-21 | Not listed | National Institute of Animal Health | Saito,T;Takemae,N |
| EPI_ISL_241762 | A/black swan/Ibaraki/302C/2016 | 2016-12-30 | Not listed | National Institute of Animal Health | Saito,T;Takemae,N |
| EPI_ISL_241754 | A/great crested grebe/Ibaraki/286T/2016 | 2016-12-25 | Not listed | National Institute of Animal Health | Saito,T;Takemae,N |
| EPI_ISL_241752 | A/whooper swan/Ibaraki/301T/2016 | 2016-12-28 | Not listed | National Institute of Animal Health | Saito,T;Takemae,N |
| EPI_ISL_241750 | A/mute swan/Ibaraki/262C/2016 | 2016-12-20 | Not listed | National Institute of Animal Health | Saito,T;Takemae,N |
| EPI_ISL_241749 | A/mute swan/Ibaraki/263T/2016 | 2016-12-20 | Not listed | National Institute of Animal Health | Saito,T;Takemae,N |
| EPI_ISL_239802 | A/Common-coot/Egypt/CA285/2016 | 2016-11-26 | National Laboratory for Veterinary Quality Control on Poultry production- Animal Health Research Inistitute | Animal Health Research Institute | Arafa,AM;Hagag,N;Zanaty,A;Elhusseiny,MH;Selim,AA;Abdelhalim,A;Erfan,A;Hassan,MK;Naguib,MM |
| EPI_ISL_234057 | A/grey heron /Uvs-Nuur Lake/20/2016 | 2016-05-25 | Research Institute of Experimental and Clinical Medicine | Research Institute of Experimental and Clinical Medicine | Kirill,Sharshov;Olga,Kurskaya;Ivan,Sobolev;Alexander,Alekseev;Alexander,Shestopalov |
| EPI_ISL_309197 | A/Brahma chicken/Belgium/6153/2017 | 2017-06-15 | Not listed | Other Database Import | VanBorm,S;Vandenbussche,F;Mathijs,E;Lambrecht,B;Steensels,M |
| EPI_ISL_301048 | A/chicken/Poland/208/2017 | 2017-02-22 | Not listed | National Veterinary Research Institut Poland, PIWet-PIB | Swieton,E;Smietanka,K |
| EPI_ISL_301038 | A/chicken/Poland/77/2017 | 2017-01-30 | Not listed | National Veterinary Research Institut Poland, PIWet-PIB | Swieton,E;Smietanka,K |
| EPI_ISL_300939 | A/chicken/Poland/116/2016 | 2016-12-30 | Not listed | National Veterinary Research Institut Poland, PIWet-PIB | Swieton,E;Smietanka,K |
| EPI_ISL_300712 | A/chicken/Poland/263/2017 | 2017-03-04 | Not listed | National Veterinary Research Institut Poland, PIWet-PIB | Swieton,E;Smietanka,K |
| EPI_ISL_298642 | A/chicken/Israel/1048/2016 | 2016-12-20 | Not listed | Other Database Import | Shkoda,I;Lapin,K;Simanov,L;Lublin,A |
| EPI_ISL_297931 | A/chicken/Anhui/MZ34/2016 | 2016-02 | Not listed | Other Database Import | Liu,K;Gu,M;Gao,R;Li,J;Liu,D;Sun,W;Hu,J;Xu,X;Wang,X;Liu,X |
| EPI_ISL_285624 | A/Chicken/South_Africa/S2017/09_0050_56/2017 | 2017-09-04 | Western Cape Provincial Veterinary Laboratory | National Institute of Communicable Diseases | Treurnicht,FK |
| EPI_ISL_285621 | A/chicken/Egypt/Gharbiya-15/2017 | 2017 | Not listed | Friedrich-Loeffler-Institut | Salaheldin,AH;AbdEl-Hamid,HS;Elbestawy,AR;Hafez,MH;Veits,J;Mettenleiter,TC;Abdelwhab,EM' |
| EPI_ISL_284685 | A/chicken/Egypt/173CAL/2017 | 2017-04 | Not listed | Other Database Import | Arafa,A;El-Husseiny,M;Mady,W;Samy,A;Adel,A;Yehia,N;Zanaty,A;Hagag,N;Erfan,A;Selim,A;Hasan,W;Hassan,M |
| EPI_ISL_283969 | A/Chicken/Yunnan/YN-12/2016 (H5N6) | 2016-06-21 | South China Agricultural University | South China Agricultural University | Jiahao,Z;Guangjie,L;Ronghua,Z;Hexing,W;Guanming,S;Bo,L;Wenbao,Q;Ming,L |
| EPI_ISL_283968 | A/Chicken/Yunnan/YN-11/2016 (H5N6) | 2016-03-21 | South China Agricultural University | South China Agricultural University | Wenbao,Q |
| EPI_ISL_283965 | A/Chicken/Yunnan/YN-8/2016 (H5N6) | 2016-09-22 | South China Agricultural University | South China Agricultural University | Jiahao,Z;Guangjie,L;Ronghua,Z;Hexing,W;Guanming,S;Bo,L;Wenbao,Q;Ming,L |
| EPI_ISL_283129 | A/chicken/Czech Republic/988-17/2017 (H5N8) | 2017-01-20 | State Veterinary Institute Prague | State Veterinary Institute Prague | Nagy,A |
| EPI_ISL_282395 | A/chicken/Xuzhou/XZ6/2016 | 2016-02-13 | Not listed | Other Database Import | Sun,W |
| EPI_ISL_277042 | A/chicken/Wuxi/7765/2016 | 2016-03-14 | Beijing Institute of Microbiology and Epidemiology | Beijing Institute of Microbiology and Epidemiology | Maijuan,Ma; Shanhui,Chen; Teng,Zhao |
| EPI_ISL_273845 | A/chicken/Togo/17RS1021-7/2017 | 2017 | Ministere de l'Agriculture, de l'Elevage et de l'Hydraulique | Istituto Zooprofilattico Sperimentale Delle Venezie | Komla,Batawui;Emilie,Go-Maro;Bianca,Zecchin;Alice,Fusaro;Alessia,Schivo;Isabella,Monne |
| EPI_ISL_272741 | A/chicken/Egypt/Alex-1/2017 | 2017-01-22 | Not listed | Other Database Import | Sedeek,ME-S;Awad,AM;Kandil,NA |
| EPI_ISL_266804 | A/chicken/Sleman/04160326/2016 | 2016-03-08 | Not listed | Other Database Import | Wibawa,H;Poermadjaja,B;Mulyawan,H;Dharmawan,R;Mahawan,T;Hutagaol,NM;Miswati,Y;Srihanto,EA;Hartawan,DHW;Hendrawati,F;Riyadi,A;Deswarni;TriHarsono,A;Hartaningsih,N;Azhar,M;Stegemen,A;McGrane,J;Rasa,FST |
| EPI_ISL_266800 | A/chicken/Majalengka/08160070-001/2016 | 2016-03-25 | Not listed | Other Database Import | Wibawa,H;Poermadjaja,B;Mulyawan,H;Dharmawan,R;Mahawan,T;Hutagaol,NM;Miswati,Y;Srihanto,EA;Hartawan,DHW;Hendrawati,F;Riyadi,A;Deswarni;TriHarsono,A;Hartaningsih,N;Azhar,M;Stegemen,A;McGrane,J;Rasa,FST |
| EPI_ISL_266796 | A/chicken/Denpasar/06160095/2016 | 2016-02 | Not listed | Other Database Import | Wibawa,H;Poermadjaja,B;Mulyawan,H;Dharmawan,R;Mahawan,T;Hutagaol,NM;Miswati,Y;Srihanto,EA;Hartawan,DHW;Hendrawati,F;Riyadi,A;Deswarni;TriHarsono,A;Hartaningsih,N;Azhar,M;Stegemen,A;McGrane,J;Rasa,FST |
| EPI_ISL_266794 | A/chicken/Sukoharjo/04160454/2016 | 2016-04-01 | Not listed | Other Database Import | Wibawa,H;Poermadjaja,B;Mulyawan,H;Dharmawan,R;Mahawan,T;Hutagaol,NM;Miswati,Y;Srihanto,EA;Hartawan,DHW;Hendrawati,F;Riyadi,A;Deswarni;TriHarsono,A;Hartaningsih,N;Azhar,M;Stegemen,A;McGrane,J;Rasa,FST |
| EPI_ISL_266793 | A/chicken/Lamongan/04160418/2016 | 2016-03-30 | Not listed | Other Database Import | Wibawa,H;Poermadjaja,B;Mulyawan,H;Dharmawan,R;Mahawan,T;Hutagaol,NM;Miswati,Y;Srihanto,EA;Hartawan,DHW;Hendrawati,F;Riyadi,A;Deswarni;TriHarsono,A;Hartaningsih,N;Azhar,M;Stegemen,A;McGrane,J;Rasa,FST |
| EPI_ISL_257449 | A/chicken/Chiba/1-1C/2017 | 2017-03-23 | National Institute of Animal Health | National Institute of Animal Health | Mine,J |
| EPI_ISL_257448 | A/chicken/Miyagi/1-5T/2017 | 2017-03-23 | National Institute of Animal Health | National Institute of Animal Health | Mine,J |
| EPI_ISL_257446 | A/chicken/Chiba/1-4T/2017 | 2017-03-23 | National Institute of Animal Health | National Institute of Animal Health | Mine,J |
| EPI_ISL_257443 | A/chicken/Chiba/1-5C/2017 | 2017-03-23 | National Institute of Animal Health | National Institute of Animal Health | Mine,J |
| EPI_ISL_257442 | A/chicken/Chiba/1-3T/2017 | 2017-03-23 | National Institute of Animal Health | National Institute of Animal Health | Mine,J |
| EPI_ISL_257440 | A/chicken/Chiba/1-5T/2017 | 2017-03-23 | National Institute of Animal Health | National Institute of Animal Health | Mine,J |
| EPI_ISL_257439 | A/chicken/Chiba/1-4C/2017 | 2017-03-23 | National Institute of Animal Health | National Institute of Animal Health | Mine,J |
| EPI_ISL_249692 | A/chicken/Germany-NI/R11406/2016 | 2016-12-24 | Not listed | Friedrich-Loeffler-Institut | Starick,E |
| EPI_ISL_249691 | A/chicken/Germany-MV/R10048/2016 | 2016-11-27 | Not listed | Friedrich-Loeffler-Institut | Starick,E |
| EPI_ISL_247397 | A/chicken/Saga/1-3T/2017 | 2017-02-04 | National Institute of Animal Health | National Institute of Animal Health | Takehiko,S |
| EPI_ISL_247392 | A/chicken/Saga/1-6T/2017 | 2017-02-04 | National Institute of Animal Health | National Institute of Animal Health | Takehiko,S |
| EPI_ISL_247385 | A/chicken/Saga/1-2C/2017 | 2017-02-04 | National Institute of Animal Health | National Institute of Animal Health | Takehiko,S |
| EPI_ISL_247384 | A/chicken/Saga/1-3C/2017 | 2017-02-04 | National Institute of Animal Health | National Institute of Animal Health | Takehiko,S |
| EPI_ISL_247380 | A/chicken/Saga/1-4C/2017 | 2017-02-04 | National Institute of Animal Health | National Institute of Animal Health | Takehiko,S |
| EPI_ISL_247379 | A/chicken/Saga/1-5T/2017 | 2017-02-04 | National Institute of Animal Health | National Institute of Animal Health | Takehiko,S |
| EPI_ISL_244534 | A/chicken/Taishun/TS2/2016 | 2016-02-19 | Not listed | Other Database Import | Chen,LJ;Tian,JH;Lin,XD;Liao,Y;Shi,M;Zhang,YZ |
| EPI_ISL_243684 | A/chicken/Miyazaki/2-5T/2017 | 2017-01-24 | Not listed | National Institute of Animal Health | TakehikoSaito |
| EPI_ISL_242409 | A/chicken/Gifu/1-6C/2017 | 2017-01-14 | Not listed | National Institute of Animal Health | Saito,T;Uchida,Y |
| EPI_ISL_242408 | A/chicken/Gifu/1-10T/2017 | 2017-01-14 | Not listed | National Institute of Animal Health | Saito,T;Uchida,Y |
| EPI_ISL_242403 | A/chicken/Gifu/1-1C/2017 | 2017-01-14 | Not listed | National Institute of Animal Health | Saito,T;Uchida,Y |
| EPI_ISL_241775 | A/chicken/Kumamoto/1-2T/2016 | 2016-12-27 | Not listed | National Institute of Animal Health | Saito,T;Takemae,N |
| EPI_ISL_241774 | A/chicken/Kumamoto/1-1C/2016 | 2016-12-27 | Not listed | National Institute of Animal Health | Saito,T;Takemae,N |
| EPI_ISL_241770 | A/chicken/Kumamoto/1-3C/2016 | 2016-12-27 | Not listed | National Institute of Animal Health | Saito,T;Takemae,N |
| EPI_ISL_241761 | A/chicken/Kumamoto/1-3T/2016 | 2016-12-27 | Not listed | National Institute of Animal Health | Saito,T;Takemae,N |
| EPI_ISL_241760 | A/chicken/Kumamoto/1-2C/2016 | 2016-12-27 | Not listed | National Institute of Animal Health | Saito,T;Takemae,N |
| EPI_ISL_241748 | A/chicken/Kumamoto/1-1T/2016 | 2016-12-27 | Not listed | National Institute of Animal Health | Saito,T;Takemae,N |
| EPI_ISL_240625 | A/chicken/Miyazaki/1-7T/2016 | 2016-12-19 | National Institute of Animal Health | National Institute of Animal Health | Uchida,Y |
| EPI_ISL_240621 | A/chicken/Miyazaki/1-7C/2016 | 2016-12-19 | National Institute of Animal Health | National Institute of Animal Health | Uchida,Y |
| EPI_ISL_240612 | A/chicken/Hokkaido/1-1C2C/2016 | 2016-12-16 | National Institute of Animal Health | National Institute of Animal Health | Uchida,Y |
| EPI_ISL_240606 | A/chicken/Miyazaki/1-4C/2016 | 2016-12-19 | National Institute of Animal Health | National Institute of Animal Health | Uchida,U |
| EPI_ISL_240531 | A/chicken/Niigata/1-4C/2016 | 2016-11-28 | National Institute of Animal Health | National Institute of Animal Health | Uchida,Y |
| EPI_ISL_240528 | A/chicken/Niigata/1-2C/2016 | 2016-11-28 | National Institute of Animal Health | National Institute of Animal Health | Uchida,Y |
| EPI_ISL_240527 | A/chicken/Niigata/1-1T/2016 | 2016-11-28 | National Institute of Animal Health | National Institute of Animal Health | Uchida,Y |
| EPI_ISL_239426 | A/chicken/Niigata/2-8T/2016 | 2016-11-30 | National Institute of Animal Health | National Institute of Animal Health | Uchida,Y |
| EPI_ISL_239423 | A/chicken/Niigata/2-7T/2016 | 2016-11-30 | National Institute of Animal Health | National Institute of Animal Health | Uchida,Y |
| EPI_ISL_234280 | A/chicken/Lebanon/157/2016 | 2016-06 | Not listed | Other Database Import | Ibrahim,E;Sirawan,A;El-Bazzal,B;Kandeil,A;Ali,M;Kayali,G |
| EPI_ISL_232042 | A/chicken/Ghana/16VIR4304-5/2016 | 2016-04-13 | Veterinary Services Directorate, Ministry of Food and Agriculture | Istituto Zooprofilattico Sperimentale Delle Venezie | Awuni,J;Aniwa,B;Zecchin,B;Fusaro,A;Schivo,A;Salviato,A;Milani,A;Marciano,S;Ormelli,S;Monne,I |
| EPI_ISL_224757 | A/chicken/Lebanon/16VIR2783-20/2016 | 2016-04-22 | Ministry of Agriculture, Animal Resources Directorate | Istituto Zooprofilattico Sperimentale Delle Venezie | ElRomeh,A;Zecchin,B;Fusaro,A;Ormelli,S;Marciano,S;Milani,A;Schivo,A;Salviato,A;Monne,I |
| EPI_ISL_304959 | A/chicken/Vietnam/QuangBinh/BoTrach1113/2017 | 2017-11-13 | Not listed | Other Database Import | Pham,HM;Pham,HK;Nguyen,VK |
| EPI_ISL_304958 | A/chicken/Vietnam/QuangBinh/BD1113/2017 | 2017-11-13 | Not listed | Other Database Import | Pham,HM;Pham,HK;Nguyen,VK |
| EPI_ISL_284771 | A/chicken/Bangladesh/31269/2016 | 2016-11-25 | Not listed | Other Database Import | Barman,S;Turner,JC;Hasan,MK;Akhtar,S;Franks,J;El-Shesheny,R;Walker,D;Seiler,P;Friedman,K;Kercher,L;Kayali,G;Jones-Engel,L;McKenzie,P;Krauss,S;Webby,RJ;Feeroz,MM;Webster,RG |
| EPI_ISL_275283 | A/chicken/Shchyolkovo/47/2017 | 2017-03-05 | Not listed | State Research Center of Virology and Biotechnology (VECTOR) | Susloparov,I;Goncharova,N;Kolosova,N;Marchenko,V;Ryzhikov,A |
| EPI_ISL_268989 | A/chicken/Czech Republic/2821-17_2/2017 (H5N8) | 2017-02-27 | State Veterinary Institute Prague | State Veterinary Institute Prague | Nagy,A |
| EPI_ISL_268955 | A/chicken/Czech Republic/1344-17/2017 (H5N8) | 2017-01-31 | State Veterinary Institute Prague | State Veterinary Institute Prague | Nagy,A |
| EPI_ISL_268946 | A/chicken/Czech Republic/1208-17_1/2017 (H5N8) | 2017-01-24 | State Veterinary Institute Prague | State Veterinary Institute Prague | Nagy,A |
| EPI_ISL_268933 | A/chicken/Czech Republic/508-17_1/2017 (H5N8) | 2017-01-12 | State Veterinary Institute Prague | State Veterinary Institute Prague | Nagy,A |
| EPI_ISL_268627 | A/Ch/NL-Zoeterwoude/16016484-021-025/2016 | 2016-12-24 | Wageningen Bioveterinary Research | Wageningen Bioveterinary Research | Beerens,N;Heutink,R;Harders,F;Verschuren-Pritz,S;Bossers,A;Koch,G;Bergervoet,S |
| EPI_ISL_268626 | A/Ch/NL-Rhenen/16016141-006/2016 | 2016-12-17 | Wageningen Bioveterinary Research | Wageningen Bioveterinary Research | Beerens,N;Heutink,R;Harders,F;Verschuren-Pritz,S;Bossers,A;Koch,G;Bergervoet,S |
| EPI_ISL_268624 | A/Ch/NL-Den Oever/16014231-001/2016 | 2016-11-15 | Wageningen Bioveterinary Research | Wageningen Bioveterinary Research | Beerens,N;Heutink,R;Harders,F;Verschuren-Pritz,S;Bossers,A;Koch,G;Bergervoet,S |
| EPI_ISL_257702 | A/chicken/Czech Republic/2644-17_1/2017 (H5N8) | 2017-02-22 | Not listed | State Veterinary Institute Prague | Nagy,A |
| EPI_ISL_257701 | A/chicken/Czech Republic/2643-17_1/2017 (H5N8) | 2017-02-22 | Not listed | State Veterinary Institute Prague | Nagy,A |
| EPI_ISL_254823 | A/chicken/Egypt/N12642E/2016 | 2016-05-08 | Not listed | Other Database Import | Rubrum,A;Jeevan,T;Kayali,G;Ali,MA;Kandeil,A;El-Shesheny,R;Webby,R |
| EPI_ISL_254816 | A/chicken/Egypt/F12505E/2016 | 2016-01-23 | Not listed | Other Database Import | Rubrum,A;Jeevan,T;Kayali,G;Ali,MA;Kandeil,A;El-Shesheny,R;Webby,R |
| EPI_ISL_252881 | A/chicken/Czech Republic/2764-17_1/2017 (H5N8) | 2017-02-24 | Not listed | State Veterinary Institute Prague | Nagy,Alexander |
| EPI_ISL_250918 | A/chicken/Czech Republic/1863-17_1/2017 (H5N8) | 2017-02-07 | Not listed | State Veterinary Institute Prague | Nagy,Alexander |
| EPI_ISL_249324 | A/chicken/Czech Republic/585-17_1/2017 (H5N8) | 2017-01-15 | State Veterinary Institute Prague | State Veterinary Institute Prague | Nagy,Alexander |
| EPI_ISL_247720 | A/chicken/Voronezh/20/2017 | 2017-01-06 | Not listed | State Research Center of Virology and Biotechnology (VECTOR) | Susloparov,I;Goncharova,N;Kolosova,N;Marchenko,V;Ryzhikov,A |
| EPI_ISL_247719 | A/chicken/Voronezh/19/2017 | 2017-01-06 | Not listed | State Research Center of Virology and Biotechnology (VECTOR) | Susloparov,I;Goncharova,N;Kolosova,N;Marchenko,V;Ryzhikov,A |
| EPI_ISL_243804 | A/chicken/Egypt/ZU120/2016 | 2016-02-26 | Not listed | Other Database Import | Hussein,A;Orabi,A;Saleh,AA;AbuEl-Magd,M;Iqbal,M |
| EPI_ISL_243797 | A/chicken/Egypt/ZU53/2016 | 2016-02-15 | Not listed | Other Database Import | Hussein,A;Orabi,A;Saleh,AA;AbuEl-Magd,M;Iqbal,M |
| EPI_ISL_243796 | A/chicken/Egypt/ZU47/2016 | 2016-02-15 | Not listed | Other Database Import | Hussein,A;Orabi,A;Saleh,AA;AbuEl-Magd,M;Iqbal,M |
| EPI_ISL_243795 | A/chicken/Egypt/ZU46/2016 | 2016-02-15 | Not listed | Other Database Import | Hussein,A;Orabi,A;Saleh,AA;AbuEl-Magd,M;Iqbal,M |
| EPI_ISL_243794 | A/chicken/Egypt/ZU34/2016 | 2016-02-15 | Not listed | Other Database Import | Hussein,A;Orabi,A;Saleh,AA;AbuEl-Magd,M;Iqbal,M |
| EPI_ISL_243789 | A/chicken/Egypt/ZU2/2016 | 2016-02-15 | Not listed | Other Database Import | Hussein,A;Orabi,A;Saleh,AA;AbuEl-Magd,M;Iqbal,M |
| EPI_ISL_242817 | A/chicken/Czech Republic/206-17_2/2017(H5N8) | 2017-01-06 | Not listed | State Veterinary Institute Prague | Nagy,Alexander |
| EPI_ISL_240109 | A/chicken/Kalmykia/2661/2016 | 2016-11-23 | Not listed | State Research Center of Virology and Biotechnology (VECTOR) | Susloparov,I;Marchenko,V;Goncharova,N;Durymanov,A;Ilyicheva,T;Ryzhikov,A |
| EPI_ISL_239419 | A/chicken/Hungary/59048/2016 | 2016-11-25 | National Food Chain Safety Office Veterinary Diagnostic Directorate Laboratory for Molecular Biology | Central Agricultural Office Veterinary Diagnostic Directorate | Dan,A |
| EPI_ISL_238896 | A/Chicken/Sweden/SVA161122KU0453/SZ0209321/2016 | 2016-11-21 | National Veterinary Institute | National Veterinary Institute | Zohari,S |
| EPI_ISL_238895 | A/Chicken/Sweden/SVA161122KU0453/SZ0209318/2016 | 2016-11-21 | National Veterinary Institute | National Veterinary Institute | Zohari,S |
| EPI_ISL_308805 | A/duck/Democratic Republic of the Congo/17RS882-5/2017 | 2017-05-14 | Not listed | Other Database Import | Twabela,A;Zecchin,B;Tshilenge,G;Sakoda,Y;Kone,P;Zamperin,G;Drago,A;Monne,I |
| EPI_ISL_308804 | A/duck/Democratic Republic of the Congo/17RS882-40/2017 | 2017-05-13 | Not listed | Other Database Import | Twabela,A;Zecchin,B;Tshilenge,G;Sakoda,Y;Kone,P;Zamperin,G;Drago,A;Monne,I |
| EPI_ISL_308803 | A/duck/Democratic Republic of the Congo/17RS882-33/2017 | 2017-05-14 | Not listed | Other Database Import | Twabela,A;Zecchin,B;Tshilenge,G;Sakoda,Y;Kone,P;Zamperin,G;Drago,A;Monne,I |
| EPI_ISL_301068 | A/wild_duck/Poland/78/2017 | 2017-02-02 | Not listed | National Veterinary Research Institut Poland, PIWet-PIB | Swieton,E;Smietanka,K |
| EPI_ISL_301053 | A/mallard/Poland/33/2017 | 2017-01-20 | Not listed | National Veterinary Research Institut Poland, PIWet-PIB | Swieton,E;Smietanka,K |
| EPI_ISL_300750 | A/domestic_duck/Poland/88/2016 | 2016-12-22 | Not listed | National Veterinary Research Institut Poland, PIWet-PIB | Swieton,E;Smietanka,K |
| EPI_ISL_300745 | A/wild duck/Poland/57/2017 | 2017-01-27 | Not listed | National Veterinary Research Institut Poland, PIWet-PIB | Swieton,E;Smietanka,K |
| EPI_ISL_297137 | A/duck/Egypt/HASHM2/2016 | 2016-01 | Not listed | Other Database Import | Ahmed,HA;AbouElez,RMM;Elsohaby,I;Tolba,HMN |
| EPI_ISL_295821 | A/duck/Vietnam/HU5-1575/2016 | 2016-08-15 | Not listed | Other Database Import | Nguyen;LT;Chu;DH;Matsuno,K;Okamatsu,M;Sakoda,Y;Jizou,M |
| EPI_ISL_295816 | A/duck/Vietnam/HU5-1574/2016 | 2016-08-15 | Not listed | Other Database Import | Chu,DH;Nguyen,LT;Okamatsu,M;Matsuno,K;Sakoda,Y;Jizou,M |
| EPI_ISL_295815 | A/duck/Vietnam/HU5-1573/2016 | 2016-08-15 | Not listed | Other Database Import | Chu,DH;Nguyen,LT;Okamatsu,M;Matsuno,K;Sakoda,Y;Jizou,M |
| EPI_ISL_295813 | A/duck/Vietnam/HU5-1571/2016 | 2016-08-15 | Not listed | Other Database Import | Chu,DH;Nguyen,LT;Okamatsu,M;Matsuno,K;Sakoda,Y;Jizou,M |
| EPI_ISL_295495 | A/Duck/Dongting/D76-1/2016 | 2016-12-28 | Not listed | Other Database Import | Ma,L;Wang,R;Zhang,X;Chen,Q |
| EPI_ISL_294124 | A/duck/France/160014/2016 | 2016-01-07 | Anses (Ploufragan-Plouzané) | ANSES Agence Nationale De Securite Sanitaire De L’alimentation | Briand,FX |
| EPI_ISL_293980 | A/duck/France/160678/2016 | 2016-07-15 | Anses (Ploufragan-Plouzané) | ANSES Agence Nationale De Securite Sanitaire De L’alimentation | Briand,FX |
| EPI_ISL_287906 | A/Duck/Netherlands/17017236-001-005/2017 | 2017-12-07 | Wageningen Bioveterinary Research | Wageningen Bioveterinary Research | Beerens,N;Heutink,R;Harders,F;Verschuren-Pritz,S;Bossers,A;Koch,G;Bergervoet,S |
| EPI_ISL_285607 | A/Duck/South_Africa/S2017/08_0340_P2/2017 | 2017-08-22 | Western Cape Provincial Veterinary Laboratory | National Institute of Communicable Diseases | Treurnicht,FK |
| EPI_ISL_285606 | A/Duck/South_Africa/S2017/08_0340_P1/2017 | 2017-08-22 | Western Cape Provincial Veterinary Laboratory | National Institute of Communicable Diseases | Treurnicht,FK |
| EPI_ISL_282396 | A/duck/Anhui/S4/2016 | 2016-01-09 | Not listed | Other Database Import | Sun,W |
| EPI_ISL_279016 | A/duck/Kagoshima/KU-d79/2016 | 2016-11-20 | Not listed | Kagoshima University | MakatoOzawa |
| EPI_ISL_279015 | A/duck/Kagoshima/KU-d66/2016 | 2016-11-20 | Not listed | Kagoshima University | MakatoOzawa |
| EPI_ISL_268633 | A/Dk/NL-Rotterdam/16014008-001-005/2016 | 2016-11-10 | Wageningen Bioveterinary Research | Wageningen Bioveterinary Research | Beerens,N;Heutink,R;Harders,F;Verschuren-Pritz,S;Bossers,A;Koch,G;Bergervoet,S |
| EPI_ISL_268629 | A/Dk/NL-Biddinghuizen/16014829-011-015/2016 | 2016-11-25 | Wageningen Bioveterinary Research | Wageningen Bioveterinary Research | Beerens,N;Heutink,R;Harders,F;Verschuren-Pritz,S;Bossers,A;Koch,G;Bergervoet,S |
| EPI_ISL_266806 | A/duck/Klaten/04160386/2016 | 2016-03-29 | Not listed | Other Database Import | Wibawa,H;Poermadjaja,B;Mulyawan,H;Dharmawan,R;Mahawan,T;Hutagaol,NM;Miswati,Y;Srihanto,EA;Hartawan,DHW;Hendrawati,F;Riyadi,A;Deswarni;TriHarsono,A;Hartaningsih,N;Azhar,M;Stegemen,A;McGrane,J;Rasa,FST |
| EPI_ISL_260769 | A/mallard/Iwate/2/2016 | 2016-12-09 | Not listed | Hokkaido University | MasatoshiOkamatsu |
| EPI_ISL_253038 | A/pochard_duck/England/SA12_157809/2016 | 2016-12-19 | Animal and Plant Health Agency (APHA) | Animal and Plant Health Agency (APHA) | Saumya,Thomas;Seekings,James;Brookes,SharonM;Reid,Scott;Essen,Stephen;Brown,IanH |
| EPI_ISL_252826 | A/duck/Hubei/ZYSYF25/2016 | 2016-02-21 | Not listed | Other Database Import | Chen,LJ;Tian,JH;Lin,XD;Liao,Y;Shi,M;Zhang,YZ |
| EPI_ISL_248634 | A/duck/Taiwan/1702004/2017 | 2017-02-03 | Not listed | Animal Health Research Institute | Yu-Ju,Lin;Li-Hsuan,Chen;Wan-Chen,Li;Yen-Ping,Chen;Yu-Pin,Liu;Fan,Lee;Wen-Jane,Tu |
| EPI_ISL_247429 | A/domestic duck/Germany-BB/R681ff/2017 | 2017-01-26 | Not listed | Friedrich-Loeffler-Institut | Starick,E |
| EPI_ISL_246662 | A/duck/Togo/16VIR6560-10/2016 | 2016-08-23 | Ministère de l´Agriculture de l´Elevage et de la Pêche | Istituto Zooprofilattico Sperimentale Delle Venezie | Komla,Batawui;Emilie,Go-Maro;Bianca,Zecchin;Alice,Fusaro;Adelaide,Milani;Annalisa,Salviato;Alessia,Schivo;Gianpiero,Zamperin;Calogero,Terregino;Isabella,Monne |
| EPI_ISL_241249 | A/domestic duck/Germany-MV/R9869/2016 | 2016-11-23 | Not listed | Friedrich-Loeffler-Institut | Starick,E |
| EPI_ISL_240012 | A/duck/France/161108h/2016 | 2016-11-28 | Not listed | ANSES Agence Nationale De Securite Sanitaire De L’alimentation | Briand,FX |
| EPI_ISL_239997 | A/decoy_duck/France/161104e/2016 | 2016-11-26 | Anses (Ploufragan-Plouzané) | ANSES Agence Nationale De Securite Sanitaire De L’alimentation | Briand,FX |
| EPI_ISL_239430 | A/muscovy duck/Aomori/2-1C/2016 | 2016-12-02 | National Institute of Animal Health | National Institute of Animal Health | Uchida,Y |
| EPI_ISL_239424 | A/muscovy duck/Aomori/1-10C/2016 | 2016-11-28 | National Institute of Animal Health | National Institute of Animal Health | Uchida,Y |
| EPI_ISL_239422 | A/muscovy duck/Aomori/2-4T/2016 | 2016-12-02 | National Institute of Animal Health | National Institute of Animal Health | Uchida,Y |
| EPI_ISL_237553 | A/duck/India/10CA01/2016 | 2016-10-17 | ICAR-National Institute of High Security Animal Diseases | ICAR-National Institute of High Security Animal Diseases | Nagarajan,Shanmugasundaram;Tripathi,Sushil;Kumar,Manoj;Murugkar,HarshadV;Tosh,Chakradhar;Singh,VijendraPal |
| EPI_ISL_232492 | A/duck/Vietnam/NCVD1584/2012 NIBRG-301 | 2016-08-31 | Not listed | National Institute for Biological Standards and Control (NIBSC) | Nicolson,C |
| EPI_ISL_221715 | A/duck/Guangdong/01.01 SZSGXJK006-G/2016 | 2016-01-01 | Institute of Microbiology, Chinese Academy of Sciences | Institute of Microbiology, Chinese Academy of Sciences | Bi,Y |
| EPI_ISL_221713 | A/duck/Guangdong/01.01 SZSGXJK005-G /2016 | 2016-01-01 | Institute of Microbiology, Chinese Academy of Sciences | Institute of Microbiology, Chinese Academy of Sciences | Bi,Y |
| EPI_ISL_221712 | A/duck/Guangdong/01.01 SZSGXJK005-Y/2016 | 2016-01-01 | Institute of Microbiology, Chinese Academy of Sciences | Institute of Microbiology, Chinese Academy of Sciences | Bi,Y |
| EPI_ISL_221710 | A/duck/Guangdong/01.01 SZSGXJK004-Y/2016 | 2016-01-01 | Institute of Microbiology, Chinese Academy of Sciences | Institute of Microbiology, Chinese Academy of Sciences | Bi,Y |
| EPI_ISL_221709 | A/duck/Guangdong/01.01 SZSGXJK003-G/2016 | 2016-01-01 | Institute of Microbiology, Chinese Academy of Sciences | Institute of Microbiology, Chinese Academy of Sciences | Bi,Y |
| EPI_ISL_279021 | A/northern pintail/Kagoshima/KU-23/2016 | 2016-11-22 | Not listed | Kagoshima University | MakatoOzawa |
| EPI_ISL_294053 | A/green-winged teal/Mississippi/16OS5996/2016 | 2016-12-19 | Not listed | Other Database Import | Killian,M;Franzen,K;Camp,P;Stuber,T;Robbe-Austerman,S;Lauterbach,S;Nolting,J;Bowman,A |
| EPI_ISL_280486 | A/common teal/Shanghai/JDS66/2016 | 2016-11-03 | Not listed | Other Database Import | He,G;Zhou,L;Zhu,C;Shi,H;Li,X;Wu,D;Liu,J;Lv,J;Hu,C;Li,Z;Wang,Z;Wang,T |
| EPI_ISL_268682 | A/Teal/NL-Ferwert/16015273-013/2016 | 2016-12-01 | Wageningen Bioveterinary Research | Wageningen Bioveterinary Research | Beerens,N;Heutink,R;Harders,F;Verschuren-Pritz,S;Bossers,A;Koch,G;Bergervoet,S |
| EPI_ISL_267136 | A/green-winged teal/Egypt/877/2016 | 2016-12-08 | Not listed | Other Database Import | Kandeil,A;Kayed,A;Moatasim,Y;Webby,R;McKenzie,PP;Kayali,G;Ali,MA |
| EPI_ISL_267135 | A/green-winged teal/Egypt/871/2016 | 2016-12-08 | Not listed | Other Database Import | Kandeil,A;Kayed,A;Moatasim,Y;Webby,R;McKenzie,PP;Kayali,G;Ali,MA |
| EPI_ISL_266424 | A/common teal/Korea/W558/2017 | 2017-01-04 | Not listed | Other Database Import | Kim,Y-I;Park,S-J;Kwon,H-I;Kim,E-H;Si,Y-J;Jeong,JH;Lee,I-W;Hiep,DN;Kwon,J-J;Choi,WS;Song,M-S;Kim,C-J;Choi,Y-K |
| EPI_ISL_266421 | A/common teal/Korea/W555/2017 | 2017-01-04 | Not listed | Other Database Import | Kim,Y-I;Park,S-J;Kwon,H-I;Kim,E-H;Si,Y-J;Jeong,JH;Lee,I-W;Hiep,DN;Kwon,J-J;Choi,WS;Song,M-S;Kim,C-J;Choi,Y-K |
| EPI_ISL_243061 | A/teal/Tottori/2/2016 | 2016-11-15 | Not listed | Other Database Import | Soda,K;Ito,H;Usui,T;Ozaki,H;Murase,T;Yamaguchi,T;Ito,T |
| EPI_ISL_279029 | A/eurasian wigeon/Kagoshima/KU-32/2016 | 2016-11-24 | Not listed | Kagoshima University | MakatoOzawa |
| EPI_ISL_269695 | A/Eurasian_Wigeon/Netherlands/21/2016 | 2016-12-05 | Not listed | Erasmus Medical Center | Poen,MJ;VanDerJeugd,HP;Vuong,O;Scheuer,RD;Kleyheeg,E;Bestebroer,TM;Begeman,L;vandenBrand,JMA;Kuiken,T;Fouchier,RAM |
| EPI_ISL_269694 | A/Eurasian_Wigeon/Netherlands/1/2016 | 2016-12-04 | Erasmus Medical Center | Erasmus Medical Center | Poen,MJ;Müskens,GJDM;VanDerJeugd,HP;Vuong,O;Scheuer,RD;Kleyheeg,E;Bestebroer,TM;Kuiken,T;Fouchier,RAM |
| EPI_ISL_269594 | A/Eurasian_Wigeon/Netherlands/22/2016 | 2016-12-14 | Erasmus Medical Center | Erasmus Medical Center | Poen,MJ;VanDerJeugd,HP;Vuong,O;Scheuer,RD;Kleyheeg,E;Bestebroer,TM;Kuiken,T;Fouchier,RAM |
| EPI_ISL_269591 | A/Eurasian_Wigeon/Netherlands/6/2016 | 2016-12-09 | Erasmus Medical Center | Erasmus Medical Center | Poen,MJ;VanDerJeugd,HP;Vuong,O;Scheuer,RD;Kleyheeg,E;Bestebroer,TM;Begeman,L;vandenBrand,JMA;Kuiken,T;Fouchier,RAM |
| EPI_ISL_268652 | A/Eur_Wig/NL-Zoeterwoude/16015702-010/2016 | 2016-12-10 | Wageningen Bioveterinary Research | Wageningen Bioveterinary Research | Beerens,N;Heutink,R;Harders,F;Verschuren-Pritz,S;Bossers,A;Koch,G;Bergervoet,S |
| EPI_ISL_268647 | A/Eur_Wig/NL-Terschelling/16015692-010/2016 | 2016-12-09 | Wageningen Bioveterinary Research | Wageningen Bioveterinary Research | Beerens,N;Heutink,R;Harders,F;Verschuren-Pritz,S;Bossers,A;Koch,G;Bergervoet,S |
| EPI_ISL_268641 | A/Eur_Wig/NL-Gouda/16015824-001/2016 | 2016-12-13 | Wageningen Bioveterinary Research | Wageningen Bioveterinary Research | Beerens,N;Heutink,R;Harders,F;Verschuren-Pritz,S;Bossers,A;Koch,G;Bergervoet,S |
| EPI_ISL_268637 | A/Eur_Wig/NL-De Waal (Texel)/16014891-004/2016 | 2016-11-27 | Wageningen Bioveterinary Research | Wageningen Bioveterinary Research | Beerens,N;Heutink,R;Harders,F;Verschuren-Pritz,S;Bossers,A;Koch,G;Bergervoet,S |
| EPI_ISL_240702 | A/Eurasian wigeon/Netherlands/1/2016 | 2016-12-04 | Erasmus Medical Center | Erasmus Medical Center | Poen,MJ;VanDerJeugd,HP;Vuong,O;Scheuer,RD;Kleyheeg,E;Müskens,GJDM;Bestebroer,TM;Koopmans,MPG;Kuiken,T;Fouchier,RAM |
| EPI_ISL_309194 | A/Anas platyrhynchos/Belgium/1899/2017 | 2017-02-27 | Not listed | Other Database Import | VanBorm,S;Vandenbussche,F;Mathijs,E;Lambrecht,B;Steensels,M |
| EPI_ISL_306660 | A/duck/Bangladesh/32502/2017 | 2017-04-19 | Not listed | Other Database Import | Barman,S;Turner,JC;Hasan,MK;Akhtar,S;Franks,J;El-Shesheny,R;Walker,D;Seiler,P;Friedman,K;Kercher,L;Kayali,G;Jones-Engel,L;McKenzie,P;Krauss,S;Webby,RJ;Feeroz,MM;Webster,RG |
| EPI_ISL_306659 | A/duck/Bangladesh/32664/2017 | 2017-04-25 | Not listed | Other Database Import | Barman,S;Turner,JC;Hasan,MK;Akhtar,S;Franks,J;El-Shesheny,R;Walker,D;Seiler,P;Friedman,K;Kercher,L;Kayali,G;Jones-Engel,L;McKenzie,P;Krauss,S;Webby,RJ;Feeroz,MM;Webster,RG |
| EPI_ISL_305416 | A/Domestic_Duck/Netherlands/EMC-2/2018 | 2018-03-13 | Erasmus Medical Center | Erasmus Medical Center | Poen,MJ;Bestebroer,TM;DeMeulder,D;Vuong,O;Scheuer,RD;NetherlandsFoodandConsumerProductSafetyAuthority,NVWA;Koopmans,MPG;Fouchier,RAM |
| EPI_ISL_302526 | A/Anas platyrhynchos/Korea/W615/2017 | 2017-12-13 | Not listed | Other Database Import | Kim,YI;Si,YJ;Kwon,HI;Kim,EH;Park,SJ;Robles,NJ;Nguyen,HD;Yu,MA;Yu,KM;Lee,YJ;Lee,MH;Choi,YK;Kim,Y-I;Si,Y-J;Yu,K-M;Choi,Y-K |
| EPI_ISL_302525 | A/Anas platyrhynchos/Korea/W614/2017 | 2017-12-13 | Not listed | Other Database Import | Kim,YI;Si,YJ;Kwon,HI;Kim,EH;Park,SJ;Robles,NJ;Nguyen,HD;Yu,MA;Yu,KM;Lee,YJ;Lee,MH;Choi,YK;Kim,Y-I;Si,Y-J;Yu,K-M;Choi,Y-K |
| EPI_ISL_290251 | A/duck/Vietnam/QuangBinh/LBM0908/2016(H5N6) | 2016-09-08 | Not listed | Other Database Import | Pham,HM;Pham,HK;NguyenVK |
| EPI_ISL_290250 | A/duck/Vietnam/QuangBinh/LBM0818/2016(H5N6) | 2016-08-18 | Not listed | Other Database Import | Pham,HM;Pham,HK;NguyenVK |
| EPI_ISL_284793 | A/duck/Bangladesh/31299/2016 | 2016-11-25 | Not listed | Other Database Import | Barman,S;Turner,JC;Hasan,MK;Akhtar,S;Franks,J;El-Shesheny,R;Walker,D;Seiler,P;Friedman,K;Kercher,L;Kayali,G;Jones-Engel,L;McKenzie,P;Krauss,S;Webby,RJ;Feeroz,MM;Webster,RG |
| EPI_ISL_284781 | A/duck/Bangladesh/31549/2016 | 2016-12-21 | Not listed | Other Database Import | Barman,S;Turner,JC;Hasan,MK;Akhtar,S;Franks,J;El-Shesheny,R;Walker,D;Seiler,P;Friedman,K;Kercher,L;Kayali,G;Jones-Engel,L;McKenzie,P;Krauss,S;Webby,RJ;Feeroz,MM;Webster,RG |
| EPI_ISL_284773 | A/duck/Bangladesh/31295/2016 | 2016-11-25 | Not listed | Other Database Import | Barman,S;Turner,JC;Hasan,MK;Akhtar,S;Franks,J;El-Shesheny,R;Walker,D;Seiler,P;Friedman,K;Kercher,L;Kayali,G;Jones-Engel,L;McKenzie,P;Krauss,S;Webby,RJ;Feeroz,MM;Webster,RG |
| EPI_ISL_269692 | A/Mallard/Netherlands/3/2017 | 2017-01-11 | Erasmus Medical Center | Erasmus Medical Center | Poen,MJ;VanDerJeugd,HP;Vuong,O;Scheuer,RD;Kleyheeg,E;Bestebroer,TM;Kuiken,T;Fouchier,RAM |
| EPI_ISL_268990 | A/mallard/Czech Republic/2822-17_1/2017 (H5N8) | 2017-02-27 | State Veterinary Institute Prague | State Veterinary Institute Prague | Nagy,A |
| EPI_ISL_268988 | A/mallard/Czech Republic/2821-17_1/2017 (H5N8) | 2017-02-27 | State Veterinary Institute Prague | State Veterinary Institute Prague | Nagy,A |
| EPI_ISL_268986 | A/mallard/Czech Republic/2820-17_1/2017 (H5N8) | 2017-02-27 | State Veterinary Institute Prague | State Veterinary Institute Prague | Nagy,A |
| EPI_ISL_268948 | A/mallard/Czech Republic/1226-17/2017 (H5N8) | 2017-01-21 | State Veterinary Institute Prague | State Veterinary Institute Prague | Nagy,A |
| EPI_ISL_268947 | A/mallard/Czech Republic/1219-17_1/2017 (H5N8) | 2017-01-25 | State Veterinary Institute Prague | State Veterinary Institute Prague | Nagy,A |
| EPI_ISL_268931 | A/mallard/Czech Republic/136-17_2/2017 (H5N8) | 2017-01-04 | State Veterinary Institute Prague | State Veterinary Institute Prague | Nagy,A |
| EPI_ISL_268664 | A/Mal/NL-IJsselmuiden/16015448-002/2016 | 2016-12-06 | Wageningen Bioveterinary Research | Wageningen Bioveterinary Research | Beerens,N;Heutink,R;Harders,F;Verschuren-Pritz,S;Bossers,A;Koch,G;Bergervoet,S |
| EPI_ISL_257703 | A/mallard/Czech Republic/2705-17/2017 (H5N8) | 2017-02-22 | Not listed | State Veterinary Institute Prague | Nagy,A |
| EPI_ISL_255913 | A/Mallard/Netherlands/2/2017 | 2017-01-07 | Erasmus Medical Center | Erasmus Medical Center | Poen,MJ;VanDerJeugd,HP;Vuong,O;Scheuer,RD;Kleyheeg,E;Lexmond,P;Eggink,WD;Müskens,GJDM;Bestebroer,TM;Koopmans,MPG;Kuiken,T;Fouchier,RAM |
| EPI_ISL_255220 | A/Mallard/Hungary/1574a/2017 | 2017-01-14 | Central Agricultural Office Veterinary Diagnostic Directorate | Central Agricultural Office Veterinary Diagnostic Directorate | Dan,A |
| EPI_ISL_255218 | A/Mallard/Hungary/1574b/2017 | 2017-01-14 | Central Agricultural Office Veterinary Diagnostic Directorate | Central Agricultural Office Veterinary Diagnostic Directorate | Dan,A |
| EPI_ISL_254745 | A/mallard duck/Korea/WA137/2017 | 2017-01-24 | Animal and Plant Quarantine Agency (O-2015) | Animal and Plant Quarantine Agency (S-2026) | Lee,YJ;Lee,EK;Song,BM;Lee,YN |
| EPI_ISL_250917 | A/duck/Czech Republic/1467-17/2017 (H5N8) | 2017-01-30 | Not listed | State Veterinary Institute Prague | Nagy,Alexander |
| EPI_ISL_239263 | A/duck/Korea/H15/2016(H5N6) | 2016-11-20 | Not listed | Animal and Plant Quarantine Agency (S-2026) | Lee,YJ |
| EPI_ISL_237966 | A/duck/Hungary/55191/2016 | 2016-11-11 | National Food Chain Safety Office Veterinary Diagnostic Directorate Laboratory for Molecular Biology | Central Agricultural Office Veterinary Diagnostic Directorate | Ronai,Z;Ursu,K;Balint,A;Szalay,A;Thuma,A;Gyuris,E;Erdelyi,K;Dan,A |
| EPI_ISL_235740 | A/mallard/Alaska/AH0088535/2016 | 2016-08-12 | Not listed | Other Database Import | Killian,ML |
| EPI_ISL_294074 | A/black duck/Tennessee/17OS0306/2017 | 2017-01-13 | Not listed | Other Database Import | Killian,M;Franzen,K;Camp,P;Stuber,T;Robbe-Austerman,S;Lauterbach,S;Nolting,J;Bowman,A |
| EPI_ISL_284813 | A/duck/Bangladesh/31103/2016 | 2016-10-24 | Not listed | Other Database Import | Barman,S;Turner,JC;Hasan,MK;Akhtar,S;Franks,J;El-Shesheny,R;Walker,D;Seiler,P;Friedman,K;Kercher,L;Kayali,G;Jones-Engel,L;McKenzie,P;Krauss,S;Webby,RJ;Feeroz,MM;Webster,RG |
| EPI_ISL_284802 | A/duck/Bangladesh/31022/2016 | 2016-10-24 | Not listed | Other Database Import | Barman,S;Turner,JC;Hasan,MK;Akhtar,S;Franks,J;El-Shesheny,R;Walker,D;Seiler,P;Friedman,K;Kercher,L;Kayali,G;Jones-Engel,L;McKenzie,P;Krauss,S;Webby,RJ;Feeroz,MM;Webster,RG |
| EPI_ISL_284801 | A/duck/Bangladesh/31097/2016 | 2016-10-24 | Not listed | Other Database Import | Barman,S;Turner,JC;Hasan,MK;Akhtar,S;Franks,J;El-Shesheny,R;Walker,D;Seiler,P;Friedman,K;Kercher,L;Kayali,G;Jones-Engel,L;McKenzie,P;Krauss,S;Webby,RJ;Feeroz,MM;Webster,RG |
| EPI_ISL_284794 | A/duck/Bangladesh/30884/2016 | 2016-09-27 | Not listed | Other Database Import | Barman,S;Turner,JC;Hasan,MK;Akhtar,S;Franks,J;El-Shesheny,R;Walker,D;Seiler,P;Friedman,K;Kercher,L;Kayali,G;Jones-Engel,L;McKenzie,P;Krauss,S;Webby,RJ;Feeroz,MM;Webster,RG |
| EPI_ISL_284792 | A/duck/Bangladesh/30682/2016 | 2016-08-21 | Not listed | Other Database Import | Barman,S;Turner,JC;Hasan,MK;Akhtar,S;Franks,J;El-Shesheny,R;Walker,D;Seiler,P;Friedman,K;Kercher,L;Kayali,G;Jones-Engel,L;McKenzie,P;Krauss,S;Webby,RJ;Feeroz,MM;Webster,RG |
| EPI_ISL_284782 | A/duck/Bangladesh/30818/2016 | 2016-09-27 | Not listed | Other Database Import | Barman,S;Turner,JC;Hasan,MK;Akhtar,S;Franks,J;El-Shesheny,R;Walker,D;Seiler,P;Friedman,K;Kercher,L;Kayali,G;Jones-Engel,L;McKenzie,P;Krauss,S;Webby,RJ;Feeroz,MM;Webster,RG |
| EPI_ISL_284768 | A/duck/Bangladesh/31096/2016 | 2016-10-24 | Not listed | Other Database Import | Barman,S;Turner,JC;Hasan,MK;Akhtar,S;Franks,J;El-Shesheny,R;Walker,D;Seiler,P;Friedman,K;Kercher,L;Kayali,G;Jones-Engel,L;McKenzie,P;Krauss,S;Webby,RJ;Feeroz,MM;Webster,RG |
| EPI_ISL_237964 | A/Mulard_duck/Hungary/54494/2016 | 2016-11-08 | National Food Chain Safety Office Veterinary Diagnostic Directorate Laboratory for Molecular Biology | Central Agricultural Office Veterinary Diagnostic Directorate | Ronai,Z;Ursu,K;Balint,A;Szalay,A;Thuma,A;Gyuris,E;Erdelyi,K;Dan,A |
| EPI_ISL_268667 | A/Sea_eagle/NL-Assen/16015398-002/2016 | 2016-12-05 | Wageningen Bioveterinary Research | Wageningen Bioveterinary Research | Beerens,N;Heutink,R;Harders,F;Verschuren-Pritz,S;Bossers,A;Koch,G;Bergervoet,S |
| EPI_ISL_259074 | A/common buzzard/Germany-SN/R1117/2017 | 2017-02-06 | Not listed | Friedrich-Loeffler-Institut | Starick,E |
| EPI_ISL_290072 | A/eastern buzzard/Oita/4401F005/2017 | 2017-01-12 | Not listed | Other Database Import | Soda,K;Usui,T;Ito,H;Ozaki,H;Yamaguchi,T;Ito,T |
| EPI_ISL_308783 | A/jungle crow/Hyogo/2803E022/2018 | 2018-03-06 | Not listed | Other Database Import | Soda,K;Ito,H;Ozaki,H;Usui,T;Murase,T;Yamaguchi,T;Ito,T |
| EPI_ISL_308782 | A/jungle crow/Hyogo/2803E011/2018 | 2018-03-03 | Not listed | Other Database Import | Soda,K;Ito,H;Ozaki,H;Usui,T;Murase,T;Yamaguchi,T;Ito,T |
| EPI_ISL_308781 | A/jungle crow/Hyogo/2803A002/2018 | 2018-03-01 | Not listed | Other Database Import | Soda,K;Ito,H;Ozaki,H;Usui,T;Murase,T;Yamaguchi,T;Ito,T |
| EPI_ISL_293500 | A/tufted duck/Hyogo/2812A009/2016 | 2016-12-28 | Not listed | Other Database Import | Soda,K;Usui,T;Ito,H;Ozaki,H;Yamaguchi,T;Ito,T |
| EPI_ISL_268677 | A/T_Dk/NL-Zeewolde/16013976-004-006/2016 | 2016-11-09 | Wageningen Bioveterinary Research | Wageningen Bioveterinary Research | Beerens,N;Heutink,R;Harders,F;Verschuren-Pritz,S;Bossers,A;Koch,G;Bergervoet,S |
| EPI_ISL_268671 | A/T_Dk/NL-Rotterdam/16014155-001/2016 | 2016-11-14 | Wageningen Bioveterinary Research | Wageningen Bioveterinary Research | Beerens,N;Heutink,R;Harders,F;Verschuren-Pritz,S;Bossers,A;Koch,G;Bergervoet,S |
| EPI_ISL_268669 | A/T_Dk/NL-Monnickendam/16013865-006-008/2016 | 2016-11-08 | Wageningen Bioveterinary Research | Wageningen Bioveterinary Research | Beerens,N;Heutink,R;Harders,F;Verschuren-Pritz,S;Bossers,A;Koch,G;Bergervoet,S |
| EPI_ISL_237958 | A/tufted duck/Germany-SH/R8444/2016 | 2016-11-07 | Not listed | Friedrich-Loeffler-Institut | Starick,E |
| EPI_ISL_237944 | A/tufted_duck/Germany/AR8444-L01987/2016 | 2016-11-07 | Not listed | Friedrich-Loeffler-Institut | Pohlmann,A |
| EPI_ISL_237922 | A/tufted duck/Denmark/17740-1/2016 | 2016-11-08 | Technical University of Denmark | Technical University of Denmark | Hjulsager,CK;Krog,JS;Kvisgaard,LK;Larsen,LE |
| EPI_ISL_295751 | A/greater scaup/Aichi/2301N021/2017 | 2017-01-06 | Not listed | Other Database Import | Soda,K;Usui,T;Ito,H;Ozaki,H;Yamaguchi,T;Ito,T |
| EPI_ISL_260058 | A/grey heron/Germany-SN/R572/2017 | 2017-01-22 | Not listed | Friedrich-Loeffler-Institut | Starick,E |
| EPI_ISL_303837 | A/grey-headed gull/Uganda/MUWRP-538/2017 | 2017-01-04 | Not listed | Other Database Import | Byarugaba,DK;Erima,B;Millard,M;Kibuuka,H;Mimbe,D;Ukuli,QA;Atim,G;Tugume,T;Mworozi,EA;Danner,A;McKenzie,P;Webby,R;Ducatez,MF;Wabwire-Mangen,F;Krauss,S |
| EPI_ISL_295792 | A/muscovy duck/Vietnam/HU8-1751/2017 | 2017-08-21 | Not listed | Other Database Import | Nguyen,LT;Chu,DH;Okamatsu,M;Matsuno,K;Sakoda,Y;Jizou,M |
| EPI_ISL_303636 | A/Mandarin_duck/Korea/K17-1885/2017 | 2017-12-23 | Avian diaseases laboratory, College of Veterinary Medicine, Konkuk University | Konkuk University | Kwon,JH;Jeong,S;Kim,YJ;Lee,SH;Song,CS |
| EPI_ISL_301787 | A/Mandarin_duck/Korea/K17-1826/2017 | 2017-12-22 | Avian diaseases laboratory, College of Veterinary Medicine, Konkuk University | Konkuk University | Kwon,JH;Jeong,S;Kim,YJ;Lee,SH;Song,CS |
| EPI_ISL_301785 | A/Mandarin_duck/Korea/K17-1815/2017 | 2017-12-22 | Avian diaseases laboratory, College of Veterinary Medicine, Konkuk University | Konkuk University | Kwon,JH;Jeong,S;Kim,YJ;Lee,SH;Song,CS |
| EPI_ISL_239269 | A/mandarin duck/Korea/WB246/2016(H5N6) | 2016-11-17 | Not listed | Animal and Plant Quarantine Agency (S-2026) | Lee,YJ |
| EPI_ISL_289501 | A/Black-headed Gull/Netherlands/29/2017 | 2017-12-18 | Erasmus Medical Center | Erasmus Medical Center | Poen,MJ;Bestebroer,TM;Kelder,L;Scheuer,RD;Bestebroer,TM;Koopmans,MPG;VanDerJeugd,HP;Fouchier,RAM |
| EPI_ISL_268866 | A/Back-headed_Gull/Netherlands/9/2016 | 2016-12-20 | Erasmus Medical Center | Erasmus Medical Center | Poen,MJ;VanDerJeugd,HP;Vuong,O;Scheuer,RD;Kleyheeg,E;Bestebroer,TM;Begeman,L;vandenBrand,JMA;Kuiken,T;Fouchier,RAM |
| EPI_ISL_302825 | A/Chicken/Netherlands/EMC-1/2018 | 2018-02-26 | Erasmus Medical Center | Erasmus Medical Center | Poen,MJ;Bestebroer,TM;DeMeulder,D;Vuong,O;Scheuer,RD;NetherlandsFoodandConsumerProductSafetyAuthority,NVWA;Koopmans,MPG;Fouchier,RAM |
| EPI_ISL_297388 | A/chicken/Republic of Macedonia/466/2017 | 2017-01-28 | Faculty of Veterinary Medicine Skopje | Croatian Veterinary Institute | Dodovski,A;Savic,V |
| EPI_ISL_297234 | A/chicken/Rostov-on-Don/1321/2017 | 2017-10-18 | Not listed | State Research Center of Virology and Biotechnology (VECTOR) | Susloparov,I;Goncharova,N;Kolosova,N;Marchenko,V;Ryzhikov,A |
| EPI_ISL_293482 | A/chicken/Miyazaki/21/2016 | 2016-12-20 | Not listed | Other Database Import | Soda,K;Usui,T;Ito,H;Ozaki,H;Yamaguchi,T;Ito,T |
| EPI_ISL_285916 | A/Chicken/South_Africa/S2017/08_0581_P2/2017 | 2017-08-30 | Western Cape Provincial Veterinary Laboratory | National Institute of Communicable Diseases | Treurnicht,FK |
| EPI_ISL_285657 | A/Chicken/South_Africa/S2017/08_0561_P2/2017 | 2017-08-29 | Western Cape Provincial Veterinary Laboratory | National Institute of Communicable Diseases | Treurnicht,FK |
| EPI_ISL_285649 | A/Chicken/South_Africa/S2017/09_0184_63/2017 | 2017-09-08 | Western Cape Provincial Veterinary Laboratory | National Institute of Communicable Diseases | Treurnicht,FK |
| EPI_ISL_285512 | A/Chicken/South_Africa/S2017/08_0336_P1/2017 | 2017-08-21 | Western Cape Provincial Veterinary Laboratory | National Institute of Communicable Diseases | Treurnicht,FK |
| EPI_ISL_309628 | A/ruddy turnstone/New Jersey/UGAI17-2914/2017 | 2017-05-24 | Not listed | Other Database Import | Tan,G;Pickett,B;Fedorova,N;Amedeo,P;Isom,R;Hu,L;Christensen,J;Durbin,A;Williams,T;Arumemi,F;Poulson,R;Bao,Y;Sanders,R;Zhdanov,S;Kiryutin,B;Lipman,DJ;Tatusova,T;Hatcher,E;Stallknecht,D |
| EPI_ISL_309617 | A/ruddy turnstone/New Jersey/UGAI17-2783/2017 | 2017-05-22 | Not listed | Other Database Import | Tan,G;Pickett,B;Fedorova,N;Amedeo,P;Isom,R;Hu,L;Christensen,J;Durbin,A;Williams,T;Arumemi,F;Poulson,R;Bao,Y;Sanders,R;Zhdanov,S;Kiryutin,B;Lipman,DJ;Tatusova,T;Hatcher,E;Stallknecht,D |
| EPI_ISL_309584 | A/ruddy turnstone/New Jersey/UGAI17-2072/2017 | 2017-05-13 | Not listed | Other Database Import | Tan,G;Pickett,B;Fedorova,N;Amedeo,P;Isom,R;Hu,L;Christensen,J;Durbin,A;Williams,T;Arumemi,F;Poulson,R;Bao,Y;Sanders,R;Zhdanov,S;Kiryutin,B;Lipman,DJ;Tatusova,T;Hatcher,E;Stallknecht,D |
| EPI_ISL_309581 | A/ruddy turnstone/New Jersey/UGAI17-1991/2017 | 2017-05-13 | Not listed | Other Database Import | Tan,G;Pickett,B;Fedorova,N;Amedeo,P;Isom,R;Hu,L;Christensen,J;Durbin,A;Williams,T;Arumemi,F;Poulson,R;Bao,Y;Sanders,R;Zhdanov,S;Kiryutin,B;Lipman,DJ;Tatusova,T;Hatcher,E;Stallknecht,D |
| EPI_ISL_307640 | A/ruddy turnstone/New Jersey/UGAI17-1963/2017 | 2017-05-12 | Not listed | Other Database Import | Tan,G;Pickett,B;Fedorova,N;Amedeo,P;Isom,R;Hu,L;Christensen,J;Durbin,A;Williams,T;Arumemi,F;Poulson,R;Bao,Y;Sanders,R;Zhdanov,S;Kiryutin,B;Lipman,DJ;Tatusova,T;Hatcher,E;Stallknecht,D |
| EPI_ISL_303854 | A/ruddy turnstone/Delaware Bay/218/2017 | 2017-05-21 | Not listed | Other Database Import | Not listed |
| EPI_ISL_303849 | A/ruddy turnstone/Delaware Bay/285/2017 | 2017-05-21 | Not listed | Other Database Import | Not listed |
| EPI_ISL_268929 | A/Common_Eider/Netherlands/2/2016 | 2016-12-20 | Erasmus Medical Center | Erasmus Medical Center | Poen,MJ;VanDerJeugd,HP;Vuong,O;Scheuer,RD;Kleyheeg,E;Bestebroer,TM;Begeman,L;vandenBrand,JMA;Kuiken,T;Fouchier,RAM |
| EPI_ISL_293502 | A/common porchard/Aichi/2301N017/2017 | 2017-01-04 | Not listed | Other Database Import | Soda,K;Usui,T;Ito,H;Ozaki,H;Yamaguchi,T;Ito,T |
| EPI_ISL_292224 | A/pochard_duck/England/AVP_18_003254/2018 | 2018-01-10 | Animal and Plant Health Agency (APHA) | Animal and Plant Health Agency (APHA) | Seekings,James;Ellis,Richard;Brookes,SharonM;Reid,Scott;Essen,Stephen;Lewis,Nicola;Brown,IanH |
