# Supplementary material for: Co-circulation of genetically distinct highly pathogenic avian influenza A clade 2.3.4.4 (H5N6) viruses in wild waterfowl and poultry in Europe and East Asia, 2017–18
Source: Virus Evol. 2019 Apr 22;5(1):vez004. doi: 10.1093/ve/vez004 (PMC6476160; doi:10.1093/ve/vez004)
Supplement: Supplementary Data [file vez004_supp.zip › Supplemental_Table_1_Legends.docx]

**Supplemental Table 1.** Acknowledgements of authors, and originating and submitting laboratories providing the sequences to Gisaid used for phylogenetic analysis.
