# Supplementary material for: Co-circulation of genetically distinct highly pathogenic avian influenza A clade 2.3.4.4 (H5N6) viruses in wild waterfowl and poultry in Europe and East Asia, 2017–18
Source: Virus Evol. 2019 Apr 22;5(1):vez004. doi: 10.1093/ve/vez004 (PMC6476160; doi:10.1093/ve/vez004)
Supplement: Supplementary Data [file vez004_supp.zip › Supplemental_Figure_1_Legends.docx]

**Supplemental Figure 1.** Maximum likelihood tree for all 8 gene segments namely HA, NA, MP, NP, NS, PA, PB1, PB2 (A-H respectively). Tips are coloured according to HA clade: The Europe-SOI (blue), Asia-SOI (red), Chinese (orange) and human (black) HPAI H5N6 viruses, 2016-17 H5N8 viruses (green), other HPAI H5 viruses (purple) and LPAI viruses (grey). Node symbols (♦) are shown only where support value (alrt – approximate likelihood ratio test) is above 85%.
